# Evolutionary origin of vertebrate OCT4/POU5 functions in supporting pluripotency

**DOI:** 10.1038/s41467-022-32481-z

**Published:** 2022-09-21

**Authors:** Woranop Sukparangsi, Elena Morganti, Molly Lowndes, Hélène Mayeur, Melanie Weisser, Fella Hammachi, Hanna Peradziryi, Fabian Roske, Jurriaan Hölzenspies, Alessandra Livigni, Benoit Gilbert Godard, Fumiaki Sugahara, Shigeru Kuratani, Guillermo Montoya, Stephen R. Frankenberg, Sylvie Mazan, Joshua M. Brickman

**Affiliations:** 1 Novo Nordisk Foundation Center for Stem Cell Medicine (reNEW), University of Copenhagen, 3B Blegdamsvej, 2200 Copenhagen, Denmark; 2 Department of Biology, Faculty of Science, Burapha University, Chon Buri, Thailand; 3 CNRS, Sorbonne Université, Biologie Intégrative des Organismes Marins, UMR7232, F-66650 Banyuls sur Mer, France; 4 Structural Molecular Biology Group, Novo Nordisk Foundation Center for Protein Research, University of Copenhagen, 3B Blegdamsvej, 2200 Copenhagen, Denmark; 5 MRC Centre for Regenerative Medicine, Institute for Stem Cell Research, School of Biological Sciences, 5 Little France Drive, University of Edinburgh, Edinburgh, EH16 4UU UK; 6 CNRS, Sorbonne Université, UPMC Univ Paris 06, FR2424, Development and Evolution of Vertebrates Group, Station Biologique, F-29688 Roscoff, France; 7 Division of Biology, Hyogo College of Medicine, Nishinomiya, Hyogo Japan; 8 Laboratory for Evolutionary Morphology, RIKEN Center for Biosystems Dynamics Research (BDR), Kobe, Japan; 9 Department of Zoology, University of Melbourne, Melbourne, VIC 3010 Australia; 10 Present Address: CNRS, Sorbonne Université, Laboratoire de Biologie du Développement de Villefranche, UMR7009, F-06234 Villefranche sur Mer, France

**Keywords:** Embryonic stem cells, Evolutionary developmental biology, Molecular evolution, Molecular modelling, Pluripotency

## Abstract

The support of pluripotent cells over time is an essential feature of development. In eutherian embryos, pluripotency is maintained from naïve states in peri-implantation to primed pluripotency at gastrulation. To understand how these states emerged, we reconstruct the evolutionary trajectory of the *Pou5* gene family, which contains the central pluripotency factor OCT4. By coupling evolutionary sequence analysis with functional studies in mouse embryonic stem cells, we find that the ability of POU5 proteins to support pluripotency originated in the gnathostome lineage, prior to the generation of two paralogues, *Pou5f1* and *Pou5f3* via gene duplication. In osteichthyans, retaining both genes, the paralogues differ in their support of naïve and primed pluripotency. The specialization of these duplicates enables the diversification of function in self-renewal and differentiation. By integrating sequence evolution, cell phenotypes, developmental contexts and structural modelling, we pinpoint OCT4 regions sufficient for naïve pluripotency and describe their adaptation over evolutionary time.

## Abstract (web summary)

By constructing an evolutionary trajectory of the cyclostome-gnathostome Pou5 gene family and comparing the structural and phenotypic protein variations, the authors uncover the origin of functional characteristics for the pluripotency factor Oct4.

## Introduction

Pluripotency refers to the capacity of a cell to give rise to all lineages of the adult body, including the germ line. This functional property was historically defined based on the advent of mouse Embryonic Stem Cells (ESCs), which made the mouse the reference model to define and explore the molecular basis for pluripotency. As the number of culture models expanded, it became clear that pluripotent cells exist across a range of cell states and developmental windows. In mammals, pluripotent cells can be found throughout distinct developmental stages in vivo, transitioning from an initial naïve state to a lineage primed one as development progresses from pre-implantation stages to gastrulation (reviewed in ref. 1). In mouse, these two states can be captured and cells can be expanded ex vivo in well-defined culture conditions. Mouse ESCs represent a naïve pluripotent state and their gene expression pattern approximates that of the Inner Cell Mass (ICM) of pre-implantation embryos. Mouse Epiblast Stem Cells (EpiSCs) represent a primed pluripotent state, which is more reminiscent of later pre-gastrulation stages of development^2,3^. The regulation of these pluripotent states has been extensively investigated and involves the input of extrinsic signals into a complex network of transcription factors. While naïve and primed cells share expression of a number of transcription factors, including OCT4 (POU5F1), SOX2 and NANOG, the transition from a naïve to primed state involves major changes in embryonic environment, transcriptomic profile (with the downregulation and upregulation of state or stage specific pluripotency regulators such as *Esrrb*, *Prdm15, or Klf4*) and enhancer or chromatin landscapes^4–12^. These molecular changes parallel a remodelling of embryo architecture, including epithelialisation and generation of the amniotic cavity^13,14^.

While the functional definition of pluripotency is unique to mammals, the concept of pluripotent populations is central to all developmental biology. Even with a plethora of mechanistic information characterising pluripotent states in the mouse, there is a scarcity of data on their evolutionary origin and conservation across vertebrates. ESCs exhibiting either naïve or primed pluripotency have been obtained in humans and other primates^15–20^, but a clear set of distinct cell types has yet to be defined in marsupials and monotremes^21,22^. Similarly, the existence of a naïve pluripotent state in the finch embryo, based on early expression dynamics of a selection of factors exhibiting homology to pluripotency markers, remains hypothetical in the absence of established cell lines exhibiting ESC properties^23^. Altogether, the existence of naïve and primed pluripotent states, as extensively described in the mouse, remains unclear outside eutherians. An alternative approach to investigate the origin of these states is to deconstruct their evolutionary trajectory, analysing when the capacity of key members of the pluripotency network to support these states emerged during evolution. We have used this approach, focusing on class V POU domain (POU5) transcription factors (OCT4 in the mouse) at key nodes of the vertebrate tree. This small multigene family comprises two orthology classes, *Pou5f1* and *Pou5f3*, in jawed vertebrates (gnathostomes)^24,25^. While key nodes in gnathostome evolution retain both genes, why only one paralogue is retained in many vertebrate species remains a mystery. In eutherians, *Oct4*, which belongs to the *Pou5f1* class, is the only representative of the gene family and is a central regulator of pluripotency both in vivo and in vitro. It is absolutely required to establish and maintain pluripotency in all contexts, but depending on expression levels, it can also mediate differentiation into distinct embryonic lineages^26–29^. This functional complexity is confirmed by in vivo analyses, with distinct roles for this factor depending on both stage and cellular context. Prior to implantation, from early to late blastocyst stages, OCT4 is first required to maintain the ICM and inhibit trophoblast differentiation, then for specification of both primitive endoderm (PrE) and epiblast^30–32^. At later stages, the loss of OCT4 from post-implantation epiblast results in multiple abnormalities, including a general disorganisation of germ layers, impaired expansion of the primitive streak and apoptosis of Primordial Germ Cells (PGCs)^26,33,34^. In primed pluripotent cells, in vitro, the immediate phenotype in response to inducible removal of OCT4 is a loss of E-cadherin (CDH1) and impaired adhesion^35^. Thus, mouse OCT4 is required to regulate pluripotency by both supporting self-renewal and establishing competence for differentiation. It is also at the heart of both primed and naïve pluripotency networks, although it acts to regulate different sets of enhancers in these distinct pluripotent states^5^. While naïve pluripotency concerns pre-implantation and appears specific to eutherian mammals, there is support for a conserved POU5 dependent network regulating aspects of pluripotency in other species. Evidence for a conserved role of POU5s in the control of pluripotency has been obtained in frog (*Xenopus*), chick, axolotl and teleosts^36–41^. Similarly, the knock-down of OCT4 homologues in *Xenopus* and zebrafish leads to gastrulation phenotypes reminiscent of those observed in the mouse, related to impaired cell adhesion^35,42^. In these species, which have lost the *Pou5f1* class, all pluripotency-related functions are fulfilled by POU5F3 rather than POU5F1.

A phenotypic complementation, or rescue assay, for OCT4 has been developed in mouse ESCs, providing a means to evaluate the ability of heterologous POU5 proteins to substitute for OCT4 in the support of naïve pluripotency and in the control of the balance between self-renewal and differentiation^43^. POU5 proteins from different species exhibit varying abilities to rescue in this assay, irrespective of the orthology class. For instance, human, platypus and axolotl POU5F1s, as well as *Xenopus* XlPOU91 (XlPOU5F3.1), one of the three POU5F3 forms identified in this species, are endowed with a similar rescue capacity, indicating that they harbour essential structural determinants required to support naïve pluripotency in mouse ESCs. In contrast, moderate or undetectable rescue ability was observed for chick and zebrafish POU5F3, respectively^36,38^. The existence of homologues with varying OCT4-like activity suggests that the role of this factor in pluripotency has undergone functional diversification across vertebrates.

In this work, we take advantage of the OCT4 complementation system to explore when POU5 proteins acquired the capacity to fulfil mouse OCT4 functions and how they evolved in the context of the duplication that gave rise to the POU5F1 and POU5F3 forms. Our results indicate that the capacity of POU5 proteins to support naïve pluripotency is a gnathostome characteristic, which emerged prior to the duplication giving rise to the *Pou5f1* and *Pou5f3* orthology classes and was elaborated in the sarcopterygian lineage. This was a result of a stepwise process, involving specialisations of the two paralogues impacting the structural orientation of two regions of the protein that allowed neo-functionalisation and reversion. Altogether, these data unveil an ancient evolutionary history for pluripotency that suggests that the states, extensively analysed in eutherians, existed long before the advent of placental development.

## Results

### Evolutionary dynamics of the *Pou5* gene family in vertebrates

**Fig. 1:**
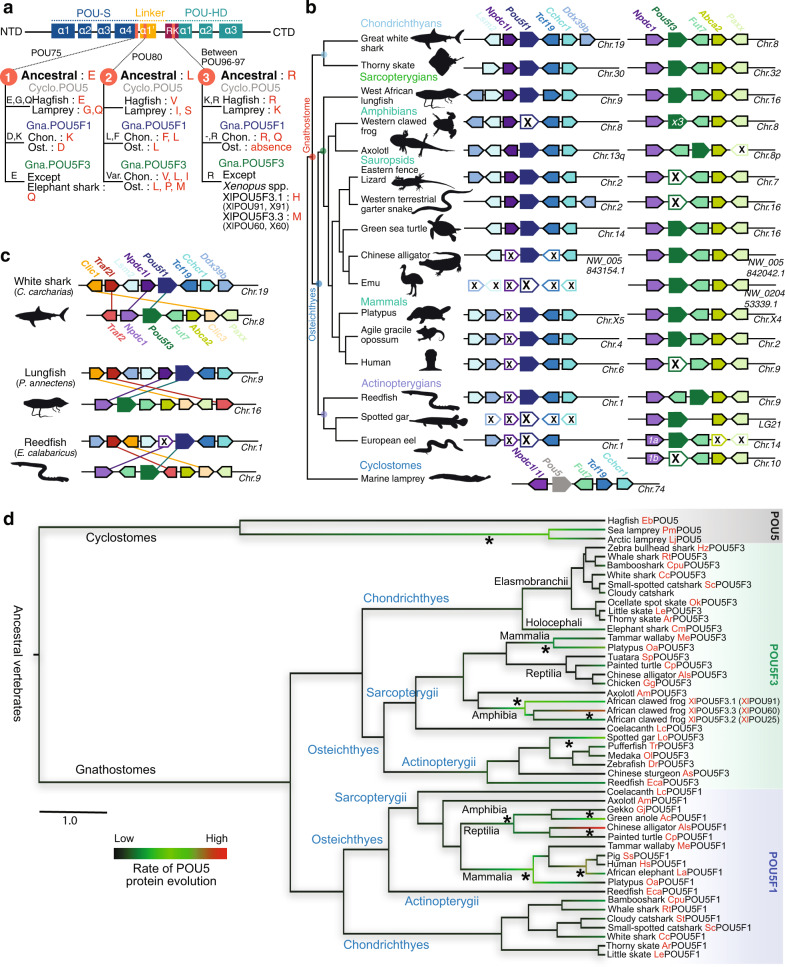
Evolution of the POU5 family in vertebrates. **a** Differences between gnathostome POU5F1 and POU5F3 proteins in the POU domain. Ancestral residues prior to the duplication generating the gnathostome paralogues are shown in bold for positions POU75, POU80 and POU96/97 of POU5F1 (Gna.POU5F1, gnathostome POU5F1, blue) and POU5F3 (Gna.POU5F3, gnathostome POU5F3, green). Residues found in cyclostomes (Cyclo.POU5, cyclostome POU5, grey) at these positions are also shown. NTD, CTD, N/C-terminal domains. **b** *Pou5* synteny conservation, genes are depicted by coloured arrows, with a single arrow for the three *Xenopus* spp. *Pou5f3* replicates. (see Supplementary Fig. 2 for additional synteny analysis). Black crosses indicate a missing gene in the genomic data analysed. In gnathostomes, genes showing conserved syntenies with *Pou5f1* (in blue) and *Pou5f3* (in green) are shown in left and right panels. In lampreys, the single homologue found for *Pou5* is shown in grey, below its gnathostome counterparts with conserved syntenies to the gnathostome *Pou5f1* (*Tcf19*, *Cchcr1*) and *Pou5f3* (*Fut7*) loci. **c** Conserved syntenies between pairs of paralogous genes (*Clic1*/*Clic3*, *Traf2l*/*Traf2* and *Npdc1l/Npdc1* respectively in yellow, red and purple arrows) found in the vicinity of gnathostome *Pou51* and *Pou53* genes in the great white shark, lungfish and reedfish. These syntenies are broadly conserved in chondrichthyans, sarcopterygians and actinopterygians (see Supplementary Fig. 2). **d** Phylogenetic tree showing the evolutionary rates of cyclostome POU5, gnathostome POU5F1 and POU5F3 sequences in different vertebrate lineages. Evolutionary rates were calculated from the alignment of POU-specific domain, linker and homeodomain (Supplementary Data 1–2) using BEAST (Supplementary Note 1), by imposing the monophyly of gnathostome POU5F1 and POU5F3 and the species phylogeny within these groups (POU5F1, *n* = 47 orthologues and POU5F3, *n* = 37 orthologues; Supplementary Data 2). They are represented along tree branches in black, green to red from low, moderate to high. Asterisks show branches in which accelerations of evolutionary rates have taken place. The scale bar corresponds to the average number of amino acid changes per site. Abbreviations used Chon, Chondrichthyes, Ost, Osteichthyes and spp, multiple species. Species name abbreviations are listed in Supplementary Data 2.

Previous characterisation of *Pou5f1* and *Pou5f3* has highlighted multiple losses of either one of the two paralogues in many osteichthyan (including tetrapod) lineages^24^. To explore the evolutionary dynamics of the *Pou5* gene family across vertebrates, we performed a comprehensive survey of these genes in a broad sampling of vertebrates, taking advantage of available genomic databases (Supplementary Data 1–2). Deduced amino acid sequences were submitted to sequence comparisons, phylogenetic and synteny analyses (Fig. 1a–c). All vertebrate full-length coding sequences predicted from our genomic searches exhibited a very similar organisation into five coding exons, with conserved locations of intron-exon boundaries, albeit with a reverse order between exons 4–5 and 2–3 in *E. burgeri*, possibly related to a genome assembly error (Supplementary Data 2). Their assignment to the *Pou5* gene family is supported by the high level of conservation of the POU-specific domain and POU homeodomain with residues identified as POU5 synapomorphies (L146^(POU16)^, K149^(POU19)^, C245^(POU115)^, Supplementary Fig. 1; ref. 44) and the presence of a N-terminal motif shared by all POU5 proteins (Supplementary Fig. 1). While sequence comparisons highlight a few signature residues of osteichthyan POU5F1 and POU5F3 in the POU-specific domain and homeodomain (residue D/E at D205^(POU75)^ and residue -/R between K226^(POU96)^ – R227^(POU97)^; Fig. 1a; ref. 24), these candidate class hallmarks are not maintained in orthologous chondrichthyan sequences, suggesting the fixation of novel selective constraints in the osteichthyan lineage (Fig. 1a). Lamprey and hagfish POU5 share several residues not found in their gnathostome counterparts, supporting the monophyly of cyclostome POU5 (Supplementary Fig. 1).

In osteichthyans, *Pou5f1* and/or *Pou5f3*-related genes can be unambiguously identified in all species analysed. Furthermore, this analysis confirmed a complex pattern of paralogue loss/retention: (i) the presence of both forms in the last common ancestor of sarcopterygians (e.g. lungfish), (ii) independent *Pou5f1* losses/*Pou5f3* retention in actinopterygians (except reedfish), anurans (e.g. frog) and birds (e.g. emu) and (iii) independent *Pou5f3* losses/*Pou5f1* retention in eutherians (e.g. human and mouse) and squamates (e.g. lizard and snake) (Fig. 1b; Supplementary Fig. 2). It also resolves the timing of paralogue loss/retention events with an increased resolution. For instance, we identified an unambiguous *Pou5f3* coding sequence in the tuatara *Sphenodon punctatus* (Supplementary Data 1–2), which implies that the loss of this paralogue in squamates followed their split from sphenodonts. Similarly, both *Pou5f1* and *Pou5f3* can be identified in the genome of *Alligator sinensis* (Fig. 1b), in line with a retention of both paralogues not only in turtles as previously documented^24^, but also in archosaurs, their sister group, prior to the loss of *Pou5f1* in birds. In actinopterygians, both paralogues are present in the reedfish *Erpechtoichtys calabaricus*, implying that the loss of *Pou5f1* previously documented in this group followed the split between cladistians and actinopteri (Fig. 1b). In all chondrichthyans (cartilaginous fishes) analysed, we obtained robust evidence for the presence of both paralogues with full-length coding sequences found in elasmobranchs, including sharks (small-spotted catshark *Scyliorhinus canicula*, white shark *Carcharodon carcharias*, brownbanded bamboo shark *Chiloscyllium punctatum*, whale shark *Rhincodon typus*) and skates (little skate *Leucoraja erinacea* and thorny skate *Amblyraja radiata*), as well as full-length *Pou5f3* and partial *Pou5f1* sequences in the holocephalan *Callorhinchus milii* (Supplementary Data 1–2). Finally, searches in the genomes of two lampreys, *Lethenteron reissneri* and *Petromyzon marinus*, and the hagfish *Eptatretus burgeri*, indicated the presence of only one *Pou5*-related coding sequence in cyclostomes. These coding sequences could not be assigned to either one of the gnathostome POU5F1 or POU5F3 classes based on amino acid sequence comparisons or phylogenetic analysis (Supplementary Data 1–2).

Synteny analyses show that gnathostome *Pou5f1* and *Pou5f3* are both located in conserved chromosomal environments. Orthologues of *Lsm2*, *Tcf19*, *Cchcr1*, and *Ddx39b* are found in the vicinity of *Pou5f1*, while *Fut7*, *Abca2*, and *Paxx* flank *Pou5f3* (Fig. 1b; Supplementary Data 2; Supplementary Fig. 2). Three pairs of paralogues are also shared between the *Pou5f1* and *Pou5f3* loci (*Clic1/Clic3*; *Traf2l/Traf2*; *Npdc1l/Npdc1*; Fig. 1c) and retained in chondrichthyans, actinopterygians and sarcopterygians, but these are detected at higher chromosomal distances, suggesting their presence in the ancestral locus prior to the duplication generating both POU5 orthologues (Fig. 1c; Supplementary Fig. 3). The chromosomal environment of the unique *Pou5* gene identified in lamprey shares characteristics of both gnathostome *Pou5f1* and *Pou5f3* loci, including conserved linkages with *Tcf19*/*Cchcr1* and *Fut7* homologues (Fig. 1b). Taken together, these data highlight the fixation of significant differences between the gnathostome *Pou5f1* and *Pou5f3* genes following the duplication which generated them.

### Heterogeneous evolutionary rates of POU5 across vertebrates

To gain insight into the molecular constraints acting on POU5 protein sequences (Supplementary Data 1-2), we characterised variations in their evolutionary rate using a bayesian Markov chain Monte Carlo algorithm (Supplementary Note 1). We first focused on the POU domain (containing the POU-specific, linker and homeodomain) in a broad sampling of vertebrates, containing all the cyclostome and chondrichthyan sequences available and a representative sampling of osteichthyans, including teleosts, amphibians, sauropsids and mammals (Fig. 1d). This analysis indicates the occurrence of the most pronounced evolutionary rate accelerations in the branches of lamprey POU5 (after their splitting from hagfish), both mammalian and reptilian POU5F1, mammalian POU5F3 (but not sauropsid POU5F3) and the three *Xenopus* POU5F3 proteins (but not their single copy counterpart in salamander). A remarkably high evolutionary rate is also observed in crocodiles for POU5F1, prior to its loss in birds (Fig. 1d). This analysis was refined for mammalian POU5F1 and actinopterygian POU5F3, using the C-terminus in combination with the POU domain with a more exhaustive species sampling in these taxa (Supplementary Fig. 4). In mammals, higher rates of POU5F1 evolution are observed in therians than in monotremes and in eutherians compared to marsupials. Heterogeneities are also detected across eutherians, with relatively high evolutionary rates in Murinae (mouse and rats) and most rodents, as well as in Chiroptera (bats). Acceleration in evolutionary rates of POU5F3 are also detected early in the actinopterygian lineage, with a higher rate of evolution in the actinopterygian versus sarcopterygian (represented by a crossopterygian, coelacanth POU5F3) branch, as well as in the neopterygian versus chondrostean lineage. The rapid pace of evolution observed in the actinopterygian lineage may explain the reduced capacity of zebrafish POU5F3 to support OCT4-null mouse ESCs^38^, with this heterogeneity in evolutionary rates observed in teleosts unlikely to be related to hidden paralogy in the context of the whole genome duplication, known to have occurred early in the teleost lineage^45^. Both copies generated by the teleost-specific duplication of *Traf2*, *Npdc1* and *Fut7* have been retained in the teleosts analysed and, in all cases, the unique *Pou5f3* gene lies in synteny with the same paralogues (*Traf2b*, *Npdc1a*, *Fut7a*) (Supplementary Figs. 2, 3). In summary, we recurrently observe significant increases in evolutionary rates associated with paralogue gains and losses, suggesting modifications of the functional constraints acting on coding sequences. However, analysis of non-synonymous to synonymous substitution failed to reveal evidence for protein positive evolution, possibly due to the globally very high conservation of the POU-specific domain and POU homeodomain.

### Functional differences between sarcopterygian POU5s in ESCs

**Fig. 2:**
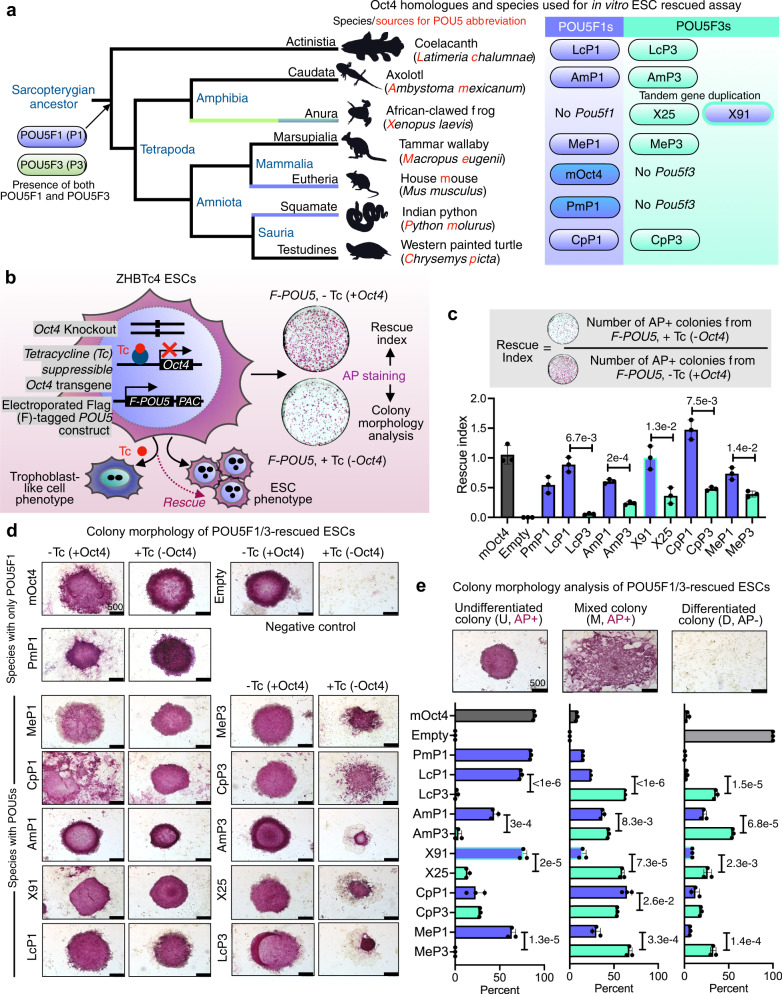
Sarcopterygian POU5F1 proteins have greater capacity to rescue mouse OCT4-null ESC cells than their POU5F3 paralogues. **a** Schematic illustration showing simplified phylogenetic tree of sarcopterygian species used for testing POU5 proteins activities and introduction to POU5 abbreviations used in this study (letters in red). **b** Experimental strategy (rescue assay) used to test the capacity of exogenous POU5 proteins (from different vertebrate species) to rescued ZHBTc4 ESCs pluripotency and self-renewal capacity, upon the addition of tetracycline (Tc). **c** Rescue indices indicating the capacity of different POU5 homologues to support ESC self-renewal. **d** Colony phenotypes obtained from POU5-transfected OCT4-null ESC cells grown in the presence or absence of mouse Oct4 (±Tc) at clonal density and stained for alkaline phosphatase (AP) activity (purple). **e** Classification and quantification of ESC colony phenotypes from rescue assay. Colonies were scored as undifferentiated (U), mixed (M), differentiated (D) and AP positive/negative (+/−) colonies (percent of each colony type/total number of colonies). Bar charts (**c**, **e**) show the mean of *n* = 3 biologically independent samples ± SD with exact *p*-values (95% confidence interval) comparing POU5F1 and POU5F3, determined by multiple unpaired (two-tailed) *t*-tests, with Welch correction. Scale bars: 500 μm.

To explore the functional evolution of POU5F1 and POU5F3 when both genes are retained, we asked whether both paralogous proteins were able to support naïve pluripotency in a heterologous mouse OCT4-rescue assay. We first focused on sarcopterygians and examined the activities of POU5 proteins from a representative sampling of species that carry both paralogues: the coelacanth (*Latimeria chalumnae*), the axolotl (*Ambystoma mexicanum*), the turtle (*Chrysemys picta bellii*) and the tammar wallaby (*Macropus eugenii*). To better visualize evolutionary trends of POU5 activity in sarcopterygians, POU5s from species that have lost either *Pou5f1* or *Pou5f3* were included, African-clawed frog (*Xenopus laevis*) and python (*Python molurus*) (Fig. 2a). Among the three *Pou5f3* paralogues produced by tandem gene duplications in the frog, only two (*XlPou5f3.1* and *XlPou5f3.2*, encoding for X91 and X25 proteins, respectively) were analysed, as the third one (*XlPou5f3.3; X60*) is dispensable for normal development (Fig. 2a). To assess POU5 activity in supporting pluripotency, we used an *Oct4*^−/−^ mouse ESC line carrying a tetracycline (Tc)-suppressible *Oct4* transgene (ZHBTc4)^27^. We introduced cDNAs encoding heterologous POU5 proteins (assession numbers of coding sequences listed in Supplementary Data 2) into ZHBTc4 cells (in the presence or absence of tetracycline) and determined the rescue potential relative to a mouse OCT4 (mOct4) cDNA control (Fig. 2b). Upon OCT4 loss, ESCs differentiate towards trophoblast, while OCT4 over-expression (when both heterologous cDNA and the *Oct4* transgene are expressed simultaneously) induces differentiation towards extra-embryonic mesoderm and endoderm^27^. With the OCT4-rescue assay we can assess the capacity of heterologous proteins to support an undifferentiated ESC phenotype in the absence of mOct4, as well as the capacity to induce differentiation when expressed in the presence of mOct4 (over-expression). The degree to which a particular POU5-rescued mOct4 activity was assessed based on a colony formation assay, comparing the number of alkaline phosphatase positive colonies (AP^+^; purple) in the presence versus the absence of tetracycline (rescue index) (Fig. 2c, upper panel).

We found that all POU5F1 orthologues from species with either one or two POU5 homologues could rescue OCT4-null ESCs, producing both high levels of undifferentiated colonies (AP^+^) and high rescue indices (Fig. 2c, d, Supplementary Fig. 5a–d). In contrast, the colonies produced by any of the POU5F3 orthologues, except X91, had varied morphologies and overall lower rescue indices (Fig. 2c, d, Supplementary Fig. 5a, b). The majority of POU5F3-rescued colonies retained an undifferentiated centre (AP^+^) surrounded by unstained differentiated cells (Fig. 2d). Quantification of the distinct morphologies produced by these POU5-rescued colonies shows that all POU5F1 proteins produced high percentages of undifferentiated colonies, while POU5F3 proteins supported high numbers of mixed and differentiated colonies (Fig. 2e). Taken together, these observations support the notion that sarcopterygian POU5 paralogues evolved distinct abilities to support pluripotency and self-renewal.

### POU5F1 and POU5F3 support distinct ESC phenotypes

**Fig. 3:**
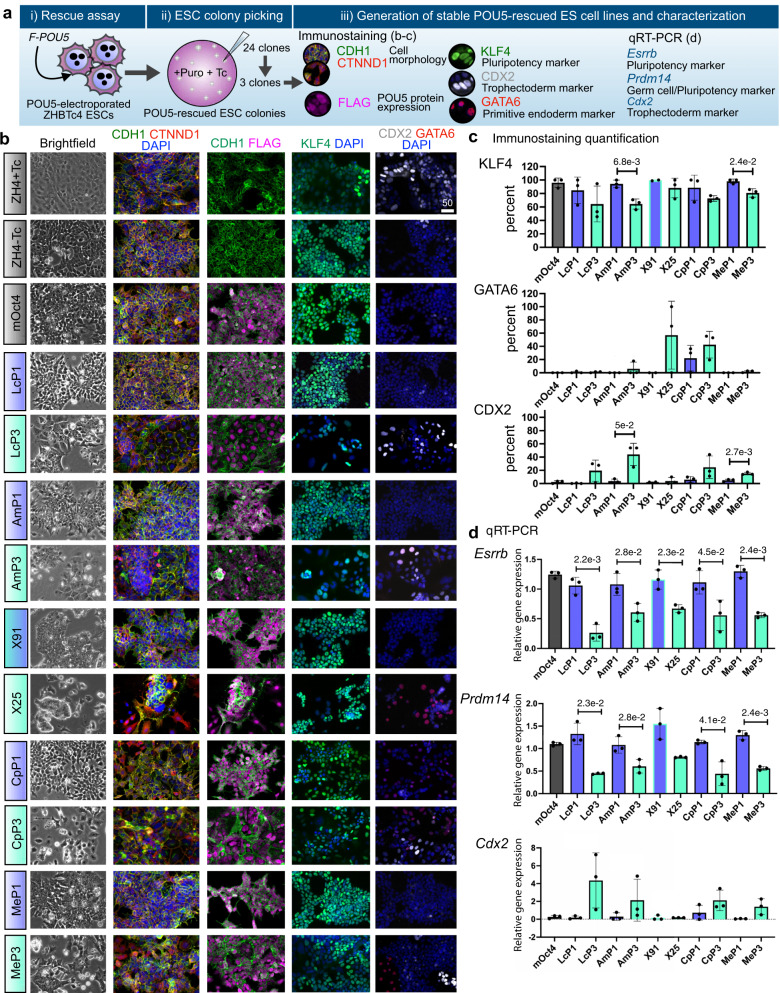
Phenotypes of ESC lines supported by sarcopterygian POU5 proteins. **a** Experimental strategy used to derive stable ZHBTc4 cell lines rescued by either POU5F1 (blue) or POU5F3 (teal) from coelacanth (Lc), axolotl (Am), frog (X), turtle (Cp) and wallaby (Me). **b** Representative immunofluorescence staining of ZHBTc4-rescued cell lines. Anti-FLAG antibodies were used to detect and localize FLAG-tagged POU5 proteins, anti-KLF4 to assess pluripotency, anti-CDX2, anti-GATA6 to assess differentiation and anti-CDH1 and anti-CTNND1 to assess cell morphology. **c** Quantification of biological replicates from immunofluorescence images showing the percentage of KLF4, CDX2, and GATA6 positive cells compared to DAPI (total nuclei). **d** Relative expression of pluripotency markers (*Esrrb* and *Prdm14*) and differentiation marker (*Cdx2*) in the rescued cell lines quantified by qRT-PCR. The abbreviations for POU5 proteins are the same as in Fig. 2a. Bar charts (**c**, **d**) show the mean of *n* = 3 biologically independent samples ± SD with exact *p*-values (95% confidence interval) comparing POU5F1 and POU5F3, determined by multiple unpaired (two-tailed) *t*-tests, with Welch correction. Scale bars: 50 μm.

To understand the differences between ESCs supported by the different POU5 proteins, we generated stable cell lines from either POU5F1- or POU5F3-rescued colonies (strategy summarised in Fig. 3a) and confirmed that all cell lines were maintained solely by the heterologous POU5s (Fig. 3b, Supplementary Fig. 6a–d). After several passages, almost all clonal lines supported by POU5F1 showed sustained self-renewal and expanded better than those supported by POU5F3 (Supplementary Fig. 6e). POU5F1-rescued ESCs resembled mOct4-rescued controls with homogenous E-cadherin (CDH1) expression and the majority of cells KLF4-positive (Fig. 3b, c). In contrast, POU5F3-rescued ESCs showed mixed morphologies (except for frog X91), with cells expressing either trophectoderm (TE; CDX2^+^) or primitive endoderm (PrE; GATA6^+^) markers (Fig. 3b, c). Moreover, ESCs supported by coelacanth, axolotl or tammar wallaby POU5F3s were prone to differentiate toward TE, while frog X25-rescues differentiated toward PrE and turtle POU5F3-rescues toward both TE and PrE (Fig. 3b, c). Consistent with our previous observations^35,38^, frog X91-rescues were indistinguishable from those supported by mOct4 or the other POU5F1 proteins (Fig. 3b, c).

In agreement with the protein expression data, qRT-PCR showed that POU5F1-rescues expressed high levels of the naïve markers *Esrrb* and *Prdm14* and low levels of the TE marker *Cdx2*, while the reverse generally held true for POU5F3 homologues (with the exception of frog X91) (Fig. 3d). Python POU5F1-rescues expressed *Nanog, Prdm14, Klf4* and *Fgf4* to similar levels as mOct4-rescued cells, suggesting that POU5F1 from species that have lost POU5F3 have similar capacity to support naïve ESC self-renewal (Supplementary Fig. 6f).

### Self-renewal support correlates with pluripotency induction

**Fig. 4:**
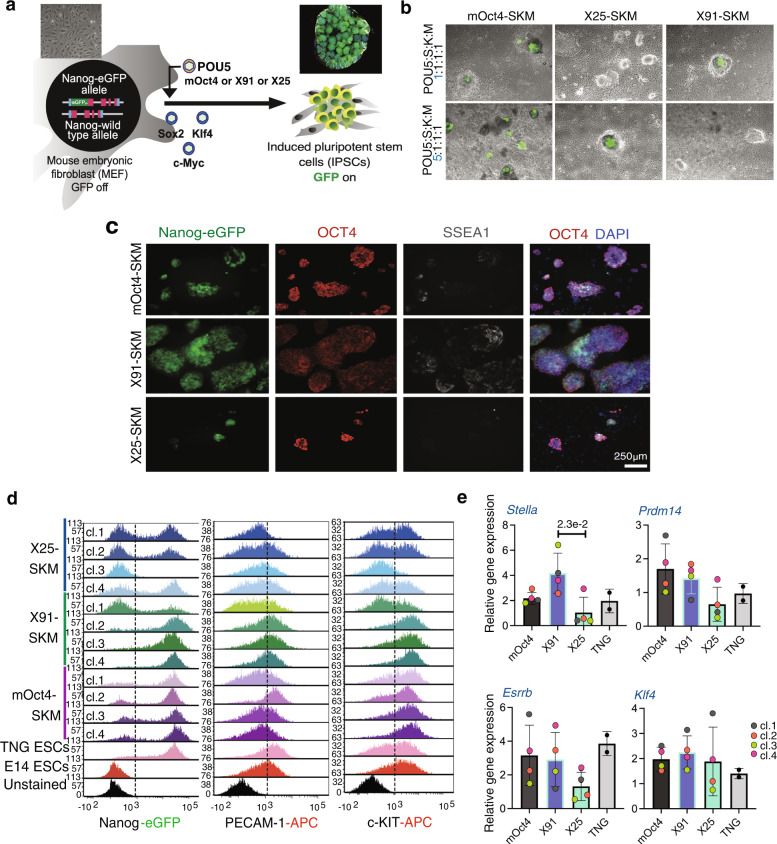
Duplicate POU5 homologues from frog display segregated functions in pluripotency establishment. **a** Experimental strategy used for murine iPSCs generation by retrovirus-based approach. Nanog-GFP mouse fibroblasts were used as a source of somatic cells to monitor the emergence of pluripotency (Nanog positive/green). *Oct4* in OSKM cocktail was replaced with either frog X25 or X91 and either low or high titre of viral infection (for *Oct4* homologues). **b** Merged images of brightfield and Nanog-GFP represent iPSC colonies at day 24 post-infection. **c** Characterisation of *Xenopus* POU5s and mOct4-derived iPSC clonal lines by immunofluorescence. Anti-Oct4 and anti-SSEA1 antibodies were used to detect a pluripotency and a germ cell marker, respectively. **d** Flow Cytometry histograms representing Nanog-GFP, PECAM-1 and c-KIT profiles of mOct4/X91/X25 SKM iPSC clonal lines. **e** Relative gene expression of germ cells (*Stella* and *Prdm14*) and naïve pluripotency (*Esrrb* and *Klf4*) markers was analysed by qRT-PCR. Data points of each clone (cl.) are also shown. Bar charts (**e**) show the mean of *n* = 4 biologically independent samples (clones) generated from three independent experiments ±SD with exact *p*-values (95% confidence interval) comparing X91 and X25 iPSCs, determined by multiple unpaired (two-tailed) *t*-tests, with Welch correction.

To test the functionality of the different POU5 homologues in another context, we compared their capacity to support ESC self-renewal with their ability to induce reprogramming. In frog embryos, X60 is expressed maternally and downregulated at gastrulation, both X91 and X25 are expressed in cells about to undergo germ layer induction^38^ and only X91 is expressed in PGCs^46^, correlating with its capacity to rescue OCT4-null ESCs. To explore the ability of these proteins to induce a pluripotent state, as well as monitor reprogramming dynamics, we used Mouse Embryonic Fibroblasts (MEFs) containing a green fluorescent protein expressed from the Nanog locus (Nanog-GFP; Fig. 4a). Reprogramming was performed using a stoichiometric ratio-based infection of equivalent amounts of retroviruses encoding a POU5 protein (mOct4, X91 or X25) and the three factors KLF4, SOX2, and c-MYC. While both mOct4 and X91 were able to induce Nanog-GFP^+^ colonies, X25 could not. (Fig. 4b, upper panel). However, Nanog-GFP^+^ colonies could be obtained when the dosage of X25 was increased to a 5:1:1:1 ratio with the viruses encoding the other factors (Fig. 4b, lower panel). When compared side by side, X91-iPSCs exhibited less spontaneous differentiation and higher levels of NANOG and SSEA1 (Fig. 4c, Supplementary Fig. 7a). Despite the induction of endogenous OCT4 (Fig. 4c), X25-iPSCs exhibited an extensive NANOG negative population (seen in only one X91-iPSC clone), similar to the spontaneous differentiation observed in X25-rescued ESCs (Fig. 3b, c). Additionally, we observed heterogeneous expression of the pluripotency markers c-KIT and PECAM-1, both within and across different iPSC clones (Fig. 4d), with the lowest number of completely reprogrammed cells, both Nanog-GFP^+^ and c-KIT^+^, in X25-iPSCs (Supplementary Fig. 7b). The enhanced capacity of X91 to induce naïve pluripotency was also observed in a higher naïve gene expression signature (Fig. 4e). Similarly, tammar wallaby POU5 proteins (MeP1 and MeP3) could induce AP^+^ iPSCs, although MeP1 was significantly more efficient, correlating with their distinct rescue indices (Supplementary Fig. 7c, d; Fig. 2). Taken together, the difference in reprogramming ability of POU5 proteins validates the functional divergence with regard to pluripotency, as seen in the OCT4-rescue assay.

### Functional segregation of naïve versus primed pluripotency

The functional analyses discussed above suggest that in sarcopterygians retaining both POU5F1 and POU5F3, the former has an enhanced ability to support naïve pluripotency while the latter supports a less stable pluripotent state, giving rise to higher levels of spontaneous differentiation. To characterise this functional divergence and generate a more comprehensive picture of the cell states supported by POU5F1 or POU5F3, we analysed the transcriptome of OCT4-null ESCs rescued by each paralogue. For this analysis, we focused on the coelacanth POU5F1 (LcP1) and POU5F3 (LcP3) forms, which diverged from their tetrapod counterparts around 400 million years ago and exhibit slow rates of evolution (Figs. 1d and 2a).

**Fig. 5:**
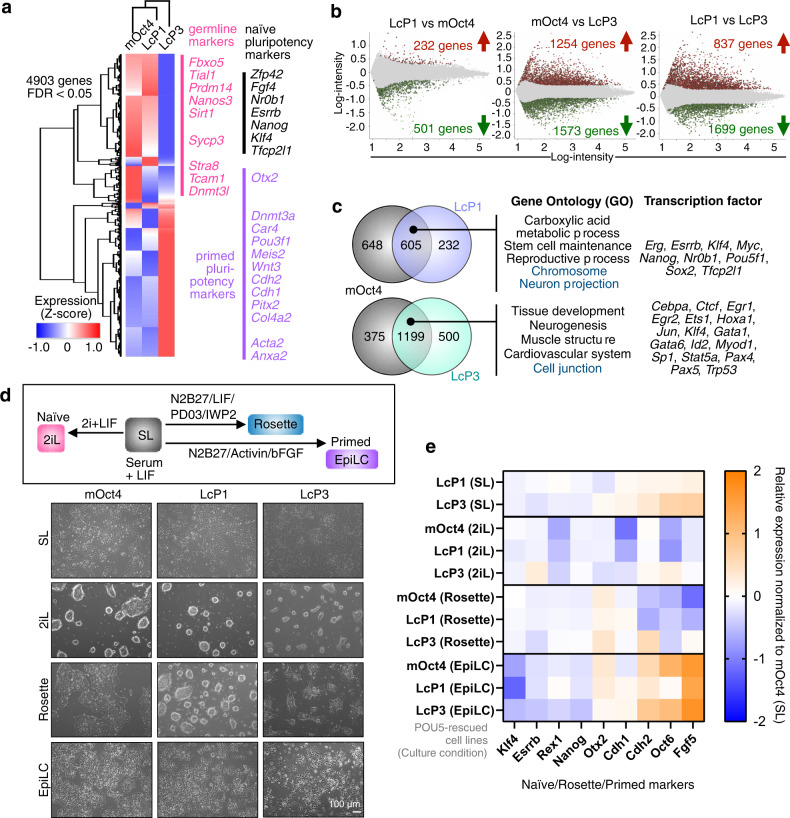
Distinct naïve-primed pluripotency phenotypes are supported by sarcopterygian POU5F1 and POU5F3 proteins. **a** Heatmap illustrating hierarchical clustering of 4903 differentially expressed genes between control (mOct4) and coelacanth POU5 (LcP1 and LcP3) rescued ESC cell lines (fold-change threshold ≥ 2 and FDR ≤ 0.05; *n* = 3 independent biological samples). The normalised expression level (z-score) of each gene is shown in three-colour format where red, white, blue indicate high, medium and low gene expression levels, respectively. **b** Log-ratio plots showing significantly over-expressed (red) and under-expressed (green) probes, based on the indicated pairwise comparisons (FDR ≤ 0.05). These gene lists were further filtered based on probes corresponding to uniquely annotated genes and with an absolute fold-change cut-off of 2. **c** Venn diagrams show a common signature in LcP1, LcP3 and mOct4 supported cells. In particular, we find a set of 605 over-expressed genes (a significant majority of both comparisons) and 1199 under-expressed genes common to both LcP1 and mOct4 supported cells, compared to cells supported by LcP3. GO-term analysis (ShinyGO v0.66: Biological Process and Cellular Component, FDR ≤ 0.05) and transcription factor targets (ShinyGO v0.66: TF.Target.RegNetwork, FDR ≤ 0.05) of these gene lists is shown on the right side of the Venn diagram. Full gene list is shown in Supplementary Data 3. **d**, **e** Naïve-primed conversion of coelacanth LcP1 and LcP3-rescued ESCs. Cell morphology of the LcP1, LcP3 and mOct4-rescued cells in naïve-primed conversion is shown in bright-field images in panel **d**. The rescued ESC lines, originally cultured in ESC medium (Serum + LIF, SL), were driven toward either naïve or primed states: (1) 2i + LIF (2iL) medium, representing the naïve state; (2) Rosette-like stem cells media, representing the intermediate state between naïve and primed; (3) Epiblast-stem-Cell-Like cells (EpiLC) medium, representing primed state. **e** Heatmap representing relative gene expression profiles (analysed by qRT-PCR) of naïve, rosette and primed pluripotency markers as well as cell adhesion markers. Averages of *n* = 3 independent biological samples in the different conditions were normalized to the mOct4 average in the SL condition (corresponding data points shown in Supplementary Fig. 8c).

Global gene expression analysis of LcP1-, LcP3- and mOct4-rescued cells identified 4903 differentially expressed genes (ANOVA with 2-fold change and False Discovery Rate (FDR) ≤ 0.05), with hierarchically clustering suggesting LcP1-rescued cells were more similar to mOct4-rescued cells (Fig. 5a). Naïve pluripotency markers, including germ cell markers, were highly expressed in both LcP1- and mOct4-rescued cells while primed pluripotency markers were highly expressed in LcP3-rescued cells. Furthermore, pairwise comparisons showed a similar pattern of up- and downregulated genes between mOct4- and LcP1-rescued cells (Fig. 5b left panel). GO enrichment analysis of genes upregulated in both mOct4- and LcP1-rescued cells when compared to LcP3-rescued cells (605 genes) identified naïve state-related categories, e.g. stem cell population maintenance and reproductive process (Fig. 5c top panel), with genes in the reproductive category most related to germ cell development, such as spermatogenesis and female gamete generation (Supplementary Fig. 8a). We next looked at genes expressed specifically in LcP3-rescued cells (1199 genes), which showed enrichment for GO terms including tissue development and cell junction (Fig. 5c lower panel, Supplementary Data 3), like E-cadherin (*Cdh1*) and N-cadherin (*Cdh2*), as well as other adhesion markers (Supplementary Fig. 8b). This link between POU5F3 proteins and positive regulators of adhesion is consistent with what we have previously described^35^ for POU5 protein function as safeguarding epithelial integrity at gastrulation and blocking differentiation as a consequence of Epithelial to Mesenchymal Transition (EMT). Furthermore, among genes common to LcP3- and mOct4-rescued cells, 31 genes were EpiSCs specific (compared to ESCs^47^) and were associated with cell adhesion and extracellular matrix (Supplementary Fig. 8b). In summary, the distinct transcriptomic profiles of ESCs supported by LcP1 and LcP3 suggest alternative roles for these paralogues in naïve versus primed pluripotency, respectively.

To test the hypothesis that paralogous POU5 proteins have specialized to support either naïve or primed pluripotency, we assessed the ability of both LcP1 and LcP3 to sustain different pluripotent states. Thus, we adapted POU5-rescued cells to either a defined naïve culture with inhibitors of MEK and GSK3 plus LIF (2iL), a culture condition that approximates an intermediate pluripotency state, known as rosette-like^13^ or a primed culture Epiblast-Like Cells (EpiLC)^47^ (Fig. 5d). In line with the transcriptome analysis (Fig. 5a–c), LcP3-rescued cells showed higher levels of primed gene expression in standard Serum/LIF (SL) culture as shown in the heatmap in Fig. 5e and Supplementary Fig. 8c. While all rescued cells appeared to eventually adopt a naïve state in 2iL conditions, LcP1 and mOct4-rescued cells adapted faster and showed normal 2iL morphology (Fig. 5d, e). In rosette medium, LcP3-rescued cells showed the highest level of *Otx2*, an early transcription factor involved in progression from pluripotency naïve towards primed states. Finally, when differentiated to EpiLCs, mOct4 and LcP3-rescued cells more effectively upregulated primed pluripotency markers *Cdh2*, *Oct6* and *Fgf5* (Fig. 5e). Taken together, our data suggest a functional segregation of the sarcopterygian POU5s, with POU5F1 supporting naïve pluripotency and POU5F3 supporting a primed pluripotency gene regulatory network associated with later stages of development, multi-lineage differentiation and gastrulation.

### Emergence of POU5-mediated mammalian pluripotency

**Fig. 6:**
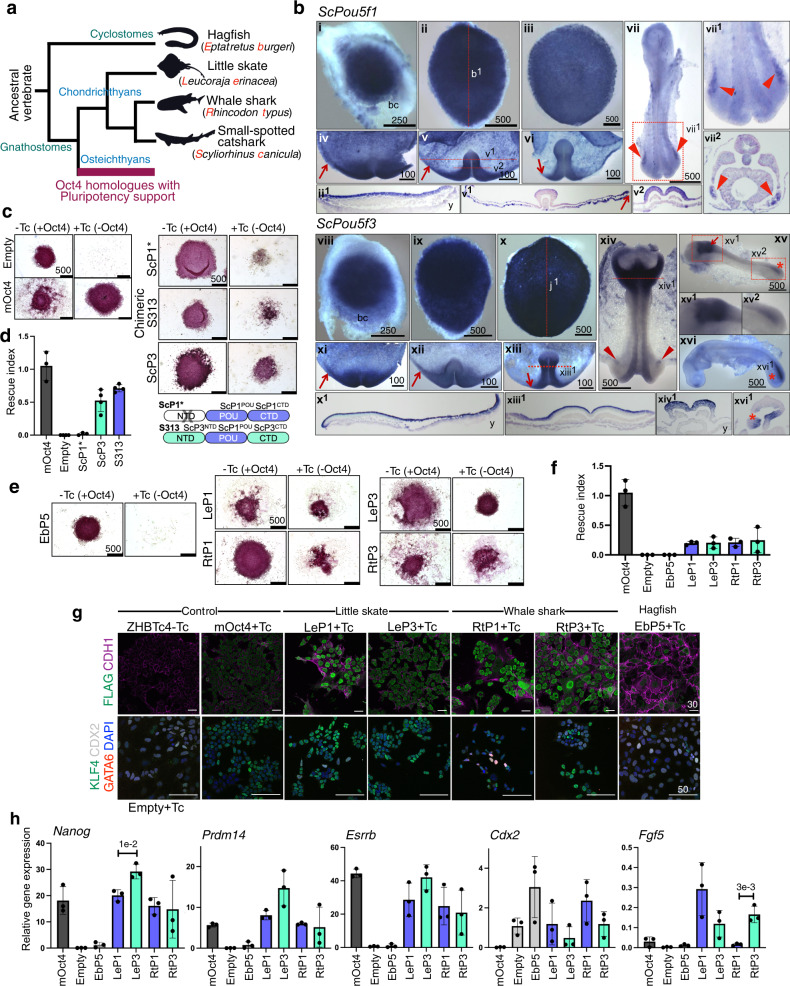
Chondrichthyan but not cyclostome POU5 proteins have the capacity to support pluripotency. **a** Schematic illustration showing simplified phylogenetic tree of cyclostome and chondrichthyan species used for testing POU5 protein activities. Abbreviation used in this study are shown in red. **b** Whole-mount views of catshark embryos following in situ hybridisations with probes for *Pou5f1* (*ScP1*) (i–vii) or *Pou5f3* (*ScP3*) (viii–xvi). Description of each panel is noted in Supplementary Note 2. **c** AP staining of ZHBTc4 ESC colonies supported by ScP1 and ScP3, cultured in the presence or absence of mOct4 (±Tc). Due to missing N-terminal domain sequence data of ScP1 (ScP1*), a chimeric form of the protein (named S313) was also tested and a cartoon of the swapping construct is shown on the bottom-right. **d** Rescue indices indicating the capacity of catshark ScP1 and ScP3 to support ESC self-renewal. **e** AP staining of ZHBTc4 ESC colonies supported by different POU5 proteins including whale shark (Rt), little skate (Le) from chondrichthyans and hagfish (Eb) from cyclostomes. **f** Rescue indices indicating the capacity of different POU5s to support ESC self-renewal. **g**, **h** Phenotypes of rescued ESC lines supported by chondrichthyan POU5 proteins compared to non-rescued cells supported by cyclostome POU5 protein. As Hagfish POU5 (EbP5) cannot rescue, EbP5 expressing colonies were picked and expanded in the absence of Tc, then treated with Tc for 4 days to remove mOct4 prior to further analysis. **g** Immunofluorescence staining of POU5-rescued cells using antibodies directed against FLAG-tagged POU5 and CDH1 (E-cadherin) (top panel) and ESC (KLF4)/differentiation (GATA6 and CDX2) markers with DAPI stained nuclei (bottom panel). **h** Relative expression of naïve pluripotency (*Nanog, Prdm14* and *Esrrb*), primed pluripotency (*Fgf5*) and trophectoderm markers (*Cdx2*) in POU5-rescued cells, quantified by qRT-PCR. Bar charts (**d**, **f**, **h**) show the mean of *n* = 3 biologically independent samples ±SD with exact *p*-values (95% confidence interval) comparing POU5F1 and POU5F3, determined by multiple unpaired (two-tailed) *t*-tests, with Welch correction. All scale bars are in μm.

To gain insight into the origin of the ability of POU5 factors to support pluripotency in vertebrates and the timing of its functional partition between the gnathostome POU5F1 and POU5F3 paralogues, we analysed the expression pattern of chondrichthyan *Pou5* genes and assessed functionality with the OCT4-rescue assay. To obtain functional data, we focused on paralogues from one batoid (little skate *Leucoraja erinacea*), and two selachians (whale shark *Rhincodon typus* and small-spotted catshark *Scyliorhinus canicula*). We also included the only POU5 identified in the cyclostome hagfish *Eptatretus burgeri*, which harbours a deduced protein sequence that is slower evolving than its counterpart in lampreys (Fig. 1d) and is therefore more likely to retain ancestral activities. A simplified phylogenetic tree of the species tested for their POU5 function is depicted in Fig. 6a. First, we analysed the expression of catshark *Pou5f1* (*ScPou5f1)* and *Pou5f3 (ScPou5f3)* from blastocoel formation to neural tube closure (Fig. 6b and Supplementary Note 2). These data show a very similar expression profile for *ScPou5f1* and *ScPou5f3*, with both being broadly expressed in the early embryo, prior to the establishment of the major embryonic lineages (Fig. 6b, i-vi and viii-xiii). At later stages of development, their territories segregate and each paralogue exhibits expression specificities, such as developing PGCs selectively expressing *ScPou5f1* (Fig. 6b, vii) or the anterior hindbrain and tailbud expressing *ScPou5f3* only (Fig. 6b, xiv–xvi).

We then tested the ability of catshark POU5 proteins (ScP1 and ScP3) to support pluripotency using the OCT4-rescue assay (Fig. 2b). Due to a missing N-terminal domain sequence in *ScPou5f1* and based on our finding that the POU domains from frog X91 sufficiently converted the activity of X25 into a POU5F1-like function in the OCT4-rescue assay (Supplementary Fig. 9a, b), we assessed the functionality of ScP1 using a chimeric protein containing the POU domains of ScP1 and the N- and C-terminal domains of ScP3 (named S313) (Fig. 6c). While the chimeric construct was able to support ESC colony formation, differences between the chimeric catshark POU5F1- and POU5F3-supported colonies were hard to distinguish (Fig. 6c, d).

Next, we assessed POU5 homologues from the other chondrichthyans (whale shark *R. typus* and little skate *L. erinacea*) and a cyclostome species (hagfish *E. burgeri*). The number of AP^+^ colonies generated in this OCT4-rescue assay showed that both POU5F1 and POU5F3 proteins from whale shark and little skate (respectively RtP1, RtP3, LeP1 and LeP3) were able to partially support ESC self-renewal in the absence of OCT4, with variable colony morphologies (Fig. 6e). In contrast, hagfish POU5 (EbP5) completely lacked rescue capacity. Unlike sarcopterygians, the average rescue indices obtained with the chondrichthyan paralogues were comparable and generally lower than those obtained with the mOct4 control (Fig. 6f).

To better characterize the functionality of chondrichthyan POU5s, we expanded rescued ESC colonies (cultured in SL + Tc) to generate stable clones and analysed the expression of pluripotency and differentiation markers. As the hagfish POU5 was unable to support any colony formation, clonal lines were generated in the presence of *Oct4* transgene (SL-Tc) and later characterized following subsequent OCT4 removal (Supplementary Fig. 9c). We confirmed that all rescued lines expressed similar levels of both heterologous cDNAs (Supplementary Fig. 9d) and exogenous POU5 proteins (Fig. 6g; Supplementary Fig. 9e). Any variations in the expression of these POU5 proteins did not correlate with their ability to rescue OCT4 activity in ESCs (Supplementary Fig. 9f).

Differences in cellular phenotypes between chondrichthyan POU5F1/3-rescued cells were assessed by immunostaining and qRT-PCR. All rescued lines exhibited a modest level of undifferentiated cells (KLF4^+^) with the exception of EbP5-rescued cells. EbP5-rescues, fixed 5 days after Tc addition, exhibited similar levels of CDX2 expression as un-rescued control ZHBTc4 cells (Empty) (Fig. 6g). The capacity of chondrichthyan POU5s to rescue pluripotency was confirmed by qRT-PCR, with robust, but variable expression of *Nanog*, *Prdm14* and *Esrrb* (Fig. 6h). Even though chondrichthyan POU5s appeared to support expression of pluripotency genes, they all exhibited low expression of differentiation markers, such as *Cdx2* and *Fgf5* (Fig. 6g, h). Taken together, these data show that all tested chondrichthyan POU5s have some capacity to support mouse ESC self-renewal, with roughly equivalent activities between paralogues, while this capacity is totally absent in the hagfish POU5. This suggests that the determinants underlying specialized POU5 pluripotency-related activities emerged in the gnathostome lineage, after the cyclostome-gnathostome split.

### Conserved structural elements of POU5s across vertebrates

**Fig. 7:**
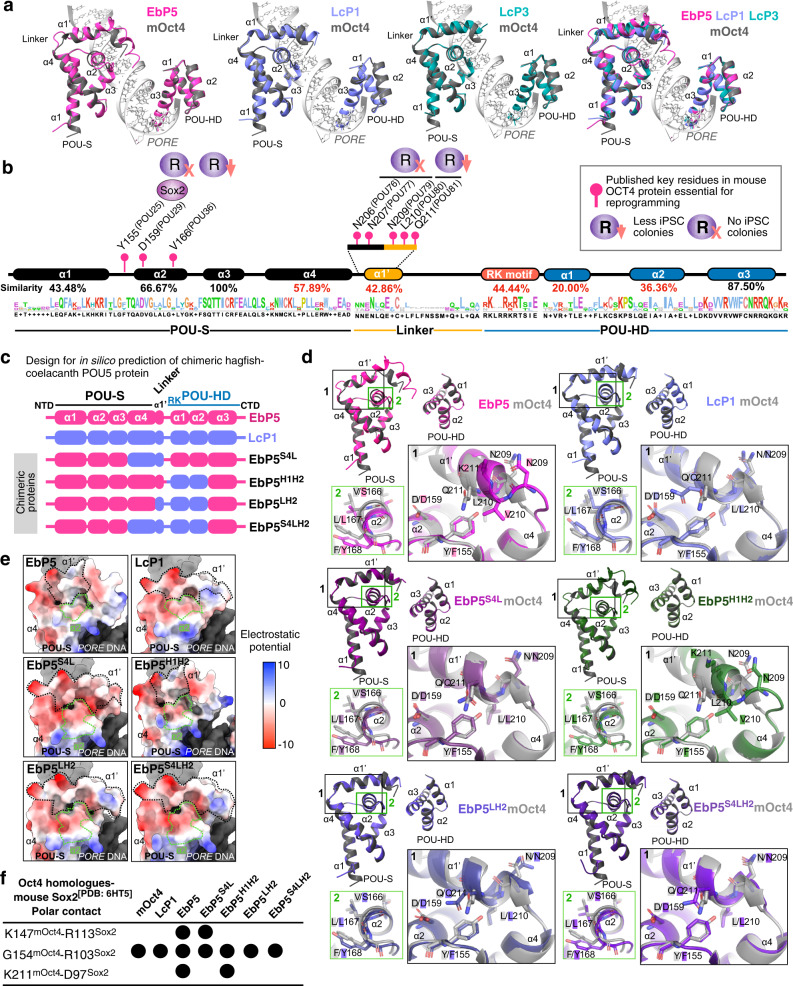
AlphaFold2-based structural models of POU5 homologues predict unique orientations for specific α-helices. **a** AlphaFold2-based structural prediction models for ordered regions (POU-S-Lα1' and POU-HD) of coelacanth (Lc) and hagfish (Eb) POU5 proteins visualized by ChimeraX with superimposition to mOct4 (grey) on the *PORE* DNA element (3L1P [https://www.ncbi.nlm.nih.gov/Structure/pdb/3L1P]). **b** Degree of conservation from the alignment of EbP5, LcP1 and mOct4 protein sequences and key residues of mOct4 (see Supplementary Fig. 10 for more details). **c** Design of EbP5-LcP1 chimeric proteins. We replaced different combinations of un-conserved regions of hagfish EbP5 (pink) with coelacanth LcP1 (lilac). **d** AlphaFold2-based structural prediction models of chimeric hagfish-coelacanth (Eb-Lc) POU5 proteins, with insets highlighting α2 from POU-S domain (Sα2) and α1' from Linker (Lα1'). Residues are also marked according to mOct4 numbering in Supplementary Fig. 10. **e** Predicted electrostatic surface potentials for chimeric proteins with focus on the POU-S-Linker region. Surface charges were determined by ChimeraX, with negatively charged areas shown in red and positively charged in blue. **f** Table summarizing prediction of polar contact interactions between POU5 homologues/chimeric Eb-Lc POU5s and mouse Sox2 using PyMol. Structural models of Eb-Lc chimeric POU5s were generated by AlphaFold2 and the POU-S domain was superimposed onto Oct4 as part of the Oct4-Sox2:UTF1 structure, retrieved from PDB (6HT5). Number of specific residues indicated in the table are related to mouse Oct4 (as shown in Supplementary Fig. 10) and mouse Sox2. Black dots represent the presence of polar contact interactions.

As the POU5s exhibit variable OCT4 rescue capacity and the POU domains in different homologues, including cyclostomes, have both highly conserved and less conserved regions at the amino acid level (Supplementary Fig. 10), we asked if putative protein structure could explain the functional differences. For this purpose, we calculated structural predictions for all POU5 homologues (Supplementary Table 1) using AlphaFold2, an AI system developed by DeepMind to predict three-dimensional protein structures based on their amino acid sequences^48^. In all POU5 models, helices were predicted in the POU-specific (POU-S; α-helices 1–4) and POU homeodomain (POU-HD; α-helices 1–3). In addition, the beginning of the linker between the POU-S and POU-HD was predicted as a helix (Linker α1'), but with lower certainty. No structural elements were predicted for the region between linker α1' and POU-HD or the N- and C-terminal tails (Supplementary Fig. 11–12). To compare the structures from different species, we asked how the two POU domains, the POU-S (including the linker α1') and the POU-HD, could interact with DNA. As a basis for this, we exploited an existing crystal structure of mOct4 bound to the *PORE* (Palindromic Oct factor Recognition Element) DNA element (3L1P, ref. 49) and created POU5-*PORE* DNA three-dimensional alignments for each POU5 homologue. Geometry validation and minimization of the resulting POU5-*PORE* DNA models was used to prevent geometrical clashes and verify the isolated structures (clash score < 10), ensuring that the analysed residues were Ramachandran favoured (Supplementary Fig. 13; Supplementary Table 2–3). From the predicted models, we determined how the DNA-bound protein structures were altered in specific paralogues and how this influenced hydrogen bonding patterns and electrostatic interactions. The superimposition of all POU5-*PORE* DNA models with mOct4-*PORE* showed similar positioning of all helices, except linker α1' (Lα1'), with hagfish (EbP5) having the greatest shift in position, suggesting a correlation with its inability to rescue mOct4 activity (Supplementary Fig. 14a). Furthermore, we observed a shift in the orientation of the second helix of the POU-S domain (Sα2) when comparing coelacanth (Lc) and hagfish proteins (Fig. 7a). We then examined the predicted hydrogen bonding (H-bond) interactions between the POU-S-L/POU-HD residues and the *PORE* DNA element for all homologues (Supplementary Fig. 14b). Generally, the predicted protein:DNA H-bonds involved residues located in helices previously reported to interact with DNA^49^ and residues conserved across all species (Supplementary Figs. 10 and 14b). Specifically, predicted H-bonds observed in all species, involved Q157^(POU27)^ and residues in the fully conserved third helix of the POU-S domain (Q174^(POU44)^ and T175^(POU45)^), in addition to the mostly conserved third helix of the POU-HD (N273^(POU143)^); of note, Q174^(POU44)^ and N273^(POU143)^ have been reported to be essential for iPSCs generation^49^ (Supplementary Figs. 10 and 14b). The greatest variation in H-bonds between homologues was predicted for POU-HD residues, showing both species and paralogue-specificity, but not correlating with naïve versus primed POU5 activity.

As the structural changes observed in different POU5 proteins occurred in the corresponding mOct4 regions identified as essential for reprogramming and support of pluripotency^49–54^ (Fig. 7b and Supplementary Fig. 10), we sought to investigate a possible correlation between the structural shifts and the lack of rescue ability for the hagfish POU5. For this purpose, we chose to generate a series of in silico predictions for chimeras of the hagfish POU5 containing elements of the coelacanth POU5F1 (LcP1), chosen because its slow evolutionary rate makes it the closest gnathostome POU5F1 to the cyclostome protein and at the same time it possesses a similar rescue index to mOct4. We focused on the least conserved domains (Fig. 7b, red percentages), with the largest swap containing the full region from Sα4 to second helix of the POU-HD (Hα2), and the others containing sections of this region (Fig. 7c). The resulting structures showed that only the EbP5^S4LH2^ and EbP5^LH2^ chimeras repositioned the Lα1' and Sα2, which were shifted in the hagfish POU5, as compared to mOct4 (Fig. 7d and Supplementary 7c). In particular, the linker together with Hα1-2 from LcP1 were required to bring Sα2 of EbP5 back in close proximity with Lα1', making the interaction of key residues (the interface formed by L210^(POU80)^ and Q211^(POU81)^ with Y/F155^(POU25)^) more favourable (Fig. 7d, box 1, Supplementary Fig. 14c). Furthermore, we investigated the electrostatic surface potentials of the POU5-*PORE* structural models, specifically focusing on the solvent-exposed surface areas with low amino acid sequence conservation, Sα2, Sα4, Lα1' and Hα1-2 (Supplementary Fig. 15). While we observed general differences in surface charge distribution between homologues, the hagfish POU5 solvent-exposed surfaces appeared to be the most neutral. Specifically, the surface charge distribution observed in the region of Sα2 and Lα1' was rescued by chimeric proteins EbP5^S4LH2^ and EbP5^LH2^, but not by EbP5^S4L^ or EbP5^H1H2^ (Fig. 7e). Similarly, when mOct4-mSox2 polar contacts were predicted, we found that EbP5 had two additional interactions that were not in mOct4 and were rescued in EbP5^S4LH2^ and EbP5^LH2^ (Fig. 7f). Taken together, our in silico modelling suggests that the region including the linker and the first two helices of the homeodomain play a key role in orienting the structure, resulting in specific helix-helix and protein-protein interactions.

**Fig. 8:**
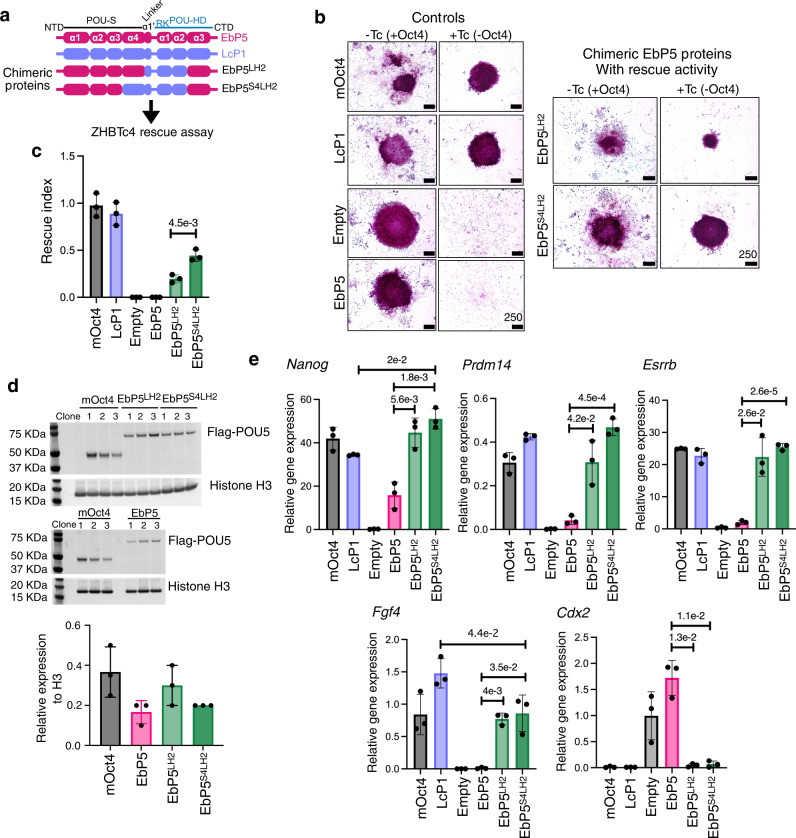
Replacing specific regions of hagfish POU5 with their coelacanth POU5F1 counterparts is sufficient to rescue pluripotency in OCT4-null mouse ESCs. **a** Design of the hagfish-coelacanth (EbP5-LcP1) chimeras used to test rescue capacity in OCT4-null mouse ESCs. **b** Colony phenotypes of ZHBTc4 cells transfected with different chimeric POU5 proteins grown in the presence or absence of mouse Oct4 (±Tc) and stained for alkaline phosphatase (AP) activity (purple). **c** Rescue index of OCT4-null ESCs rescued by chimeric hagfish POU5 proteins. **d** Western blot showing protein expression of 3xflag-tagged chimeric Eb-Lc POU5 proteins from three rescued clones per species, with quantification below. **e** Relative expression of naïve pluripotency (*Nanog, Prdm14, Esrrb* and *Fgf4*) and trophectoderm markers (*Cdx2*) in chimeric hagfish POU5-rescued ESC clonal cell lines, quantified by qRT-PCR. Bar charts (**c**, **d**, **e**) show the mean of *n* = 3 biologically independent samples ± SD with exact *p*-values (95% confidence interval) comparing chimeras, determined by multiple unpaired (two-tailed) *t*-tests, with Welch correction.

To test whether the re-orientation of Lα1' and Sα2 was sufficient to support pluripotency in vitro, we engineered two hagfish-coelacanth chimeric proteins, EbP5^S4LH2^ and EbP5^LH2^, and evaluated their functionality using the OCT4-rescue assay (Figs. 2b, 8a). Both chimeras supported the formation of undifferentiated colony, but showed differences in their proliferative ability, as seen by the reduced size of the EbP5^LH2^-rescued colonies (Fig. 8b, c). To understand the phenotypic differences between EbP5^S4LH2^ and EbP5^LH2^-rescued cells, we established clonal cell lines with stable chimeric protein expression (Fig. 8d) and compared their gene expression profiles by qRT-PCR (Fig. 8e). Both chimeras supported the expression of key pluripotency markers, such as *Nanog*, *Prdm14*, *Esrrb* and *Fgf4* and efficiently suppressed *Cdx2* expression, similarly to mOct4 and LcP1.

In conclusion, with a combination of sequence alignments, structural modelling and domain swapping, we pinpointed the region of gnathostome POU5F1 that is sufficient to inhibit differentiation and support naïve ESC self-renewal in the absence of mOct4.

## Discussion

**Fig. 9:**
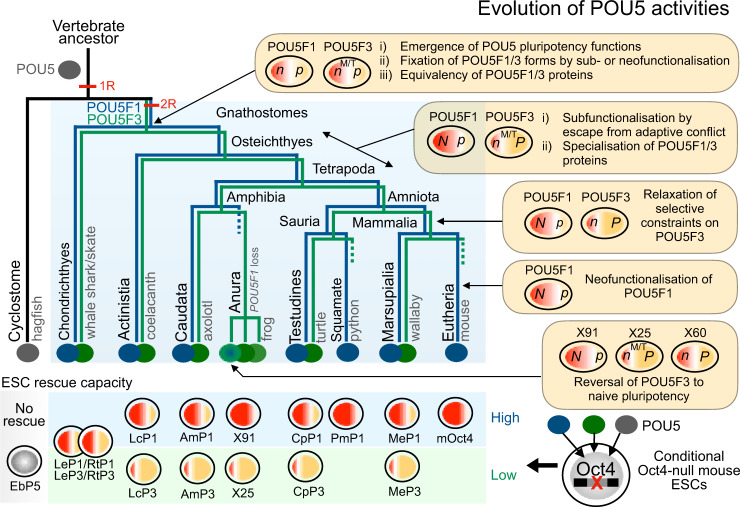
Summary of the evolution of POU5 activities in vertebrates. A simplified phylogenetic tree summarises the evolution of the *Pou5* gene family in vertebrates as inferred from genomic searches and sequence analysis. Most salient events were (1) the emergence of the family in the vertebrate lineage (black branch), (2) the maintenance of one copy in cyclostomes (grey branch) and (3) a duplication giving rise to the two gnathostome *Pou5f1* and *Pou5f3* paralogues (blue and green branches respectively), which may have been part of the 1 R/2 R whole genome duplications that took place in vertebrates. Dotted lines indicate lineages in which one paralogue was lost. Right panels point at key nodes of the tree and indicate major milestones in the functional evolution of the gene family, as inferred from expression and functional analyses of POU5F1 and POU5F3 proteins from selected species (box at the bottom of the figure shows a summary of the activities observed in the Oct4 rescue assay). These nodes include (1) the emergence of the capacity of POU5 proteins to support pluripotency, which predated the duplication generating the *Pou5f1* and *Pou5f3* genes, but is not shared by cyclostome POU5 proteins, (2) the preservation of both gnathostome paralogues possibly related to expression specificities, fixed for each form prior to the gnathostome radiation, (3) functional specialisation of paralogous proteins that took place early in the sarcopterygian lineage and could have paved the way to elaborations of naïve and primed pluripotency states of eutherians. Additional evolutionary changes, including reversals or innovations, have paralleled losses of one paralogue in anurans and in eutherians (*Pou5f1* and *Pou5f3* respectively). N/n and P/p refers to the capacity of POU5 paralogous proteins to support naïve and primed pluripotency in OCT4 rescue assays (“N” and “P” refer to a strong activity, “n” and “p” to a low one). M/T refers to specific expression traits of *Pou5f3* at the neural tube, anterior hindbrain and tailbud, conserved across gnathostomes, including chondrichthyans, which may have contributed to the preservation of this paralogue following the *Pou5f1/Pou5f3* gene duplication.

Here we show that since their emergence in vertebrates, POU5 proteins have undergone a complex stepwise evolution, enabling the eventual emergence of the naïve and primed pluripotency states of mammals. This evolutionary history involves the segregation and integration of multiple spatial and temporal inputs into a core network safe-guarding cell potency, which can be traced back to the origin of gnathostomes (Fig. 9).

Pinpointing the timing of gene losses and duplications is an essential stepping stone in understanding functional evolution of a multigene family. The combination of sequence comparisons, phylogenetic and synteny analyses reported here indicates that gnathostome *Pou5f1*/*Pou5f3*, as well as lamprey and hagfish *Pou5* genes form monophyletic groups. Together with the monophyly of cyclostomes^55^ and the recently proposed timing for the two rounds of Whole Genome Duplications (WGDs) that took place early during vertebrate evolution^56–58^, these data suggest that the *Pou5* family emerged in vertebrates and that the *Pou5f1* and *Pou5f3* classes were generated by a large-scale duplication event, possibly corresponding to the second round of vertebrate WGD (Fig. 9, Supplementary Fig. 16a–c). The finding that the hagfish POU5 protein is unable to support pluripotency, while all gnathostome POU5F1 or POU5F3 proteins tested, including chondrichthyan forms (albeit to variable degrees), exhibit some capacity to do so, indicates that the origin of the structural determinants, which underlie the regulation of the OCT4-centric pluripotency network in ESCs, can be traced back to the origin of gnathostomes, prior to the *Pou5f1*/*Pou5f3* gene duplication. Together with the expression profiles reported in all major gnathostome taxa, including chondrichthyans (this study), these data suggest that *Pou5* roles in germ cell and gastrulation stage pluripotency were fixed early in the gnathostome lineage.

Despite multiple losses of either paralogue during gnathostome evolution (ref. 24; this study), we find that both *Pou5f1* and *Pou5f3* were retained at the base of the chondrichthyan, actinopterygian and sarcopterygian lineages, as well as in the last common ancestor of actinistians, amphibians, sauropsids and mammals (Fig. 9). This suggests that distinct selective forces acted to preserve both paralogues shortly after duplication, in agreement with evolutionary models for maintenance of duplicate genes^59^. A dosage selection effect may also have been involved, consistent with overlapping early expressions of the two catshark paralogues and the dose sensitivity of OCT4 in ESCs^27^. An early specialization of each form in gnathostomes may also have been a driving force in this process. In line with this hypothesis, chondrichthyan POU5F1 and POU5F3 display unique expression characteristics, selectively maintained for each class in osteichthyans. For instance, in the catshark, the anterior hindbrain expresses *Pou5f3*, similar to chick, frog and zebrafish^36,38,60,61^ while the developing yolk sac endoderm exhibits a *Pou5f1* expression reminiscent of *Oct4* in the primitive endoderm of mammals^30,62^. These territories may reflect ancient class-specific expression features, fixed prior to gnathostome radiation, which contributed to the initial preservation of both paralogues shortly after duplication, either by neo-functionalisation, or duplication-degeneration-complementation. This expression diversification of the two classes at the regulatory level may have paved the way to subsequent specializations at the protein level, further contributing to their maintenance. Accordingly, our analysis shows that in sarcopterygians, POU5F1 orthologues from species harbouring both paralogues, were significantly more able to support naïve pluripotency, while POU5F3s showed a higher capacity to support primed pluripotency, a difference not observed in chondrichthyans. These findings suggest that the dual functionality observed for mOct4, has an alternative resolution in sarcopterygians that retain both genes, through the segregation of either naïve or primed pluripotency functions between the two paralogues. Such a specialisation of duplicates is consistent with escape from adaptive conflict evolutionary mode, whereby the duplication of an ancestral bi-functional gene results in the specialisation of each paralogue, optimising its capacity to fulfil one function, while impeding its capacity to perform the other^59^. We propose that this process led to a functional diversification of POU5F1 and POU5F3 proteins early in the osteichthyan lineage, such that POU5F1 orchestrated the preservation of the germ line, insulating it from extrinsic differentiation signals, while POU5F3 specialised to manage gastrulation specific signals, through the regulation of adhesion, migration and differentiation.

Can sequence or structure determinants of POU5 proteins, related to the complex evolutionary history and gene retention/loss pattern of the gene family, be identified? Perhaps evolutionary innovation focused on the region that was responsible for the emergence of the POU5-centric pluripotency network. Supporting this idea, we found a coding sequence in LcP1 that influences key structural elements in POU5 proteins and conveys POU5 activity to the hagfish protein, not only endowing this protein with chondrichthyan-like POU5 activity, but with POU5F1-like capacity to support naïve pluripotency. Central to these structural elements are a number of residues that are crucial for the support or induction of pluripotency by mOct4 (Fig. 7b and Supplementary Fig. 10), such as the POU-S domain residues D159^(POU29)^ required for the mOct4-mSox2 interaction and iPSC formation^53^, V166^(POU36)^ required for optimal reprogramming^49^ and a gain-of-function mutation (T152R^(POU22)^) identified in an enhanced POU (ePOU)^63^. In addition, multiple positions in the first helix of the linker region have been identified as important for reprogramming^49^, including positions N206^(POU76)^, N207^(POU77)^, N209^(POU79)^, L210^(POU80)^ and Q211^(POU81)^ and another gain-of-function mutation (E208P^(POU78)^)^63^. Simultaneous mutation of N206^(POU76)^, N207^(POU77)^, N209^(POU79)^ and L210^(POU80)^ abolishes OCT4-rescue activity^49^. However, all of these amino acids are ultimately conserved in both POU5F1 and POU5F3, and as result they cannot explain the differences in naïve versus primed pluripotency observed here. Therefore, we looked for residues that were unique to the specific paralogues. Although we identified variations within the linker, no obvious naïve motif was apparent. While position D205^(POU75)^ in mOct4 is conserved in LcP1, but is an E in LcP3, the RK motif in LcP3 contains an extra R and there is homeodomain position, L250^(POU120)^, that is conserved in mOct4 and LcP1, but is a S in LcP3. However, these specific differences are not found in X91, have not been identified via mutational screens and have no clear assigned function. Therefore, it is not our contention that these residues give POU5F1 its capacity to support naïve pluripotency. Instead, we favour the hypothesis that the coevolution of multiple changes preserved the structural integrity of protein-protein interaction surfaces, including the influence of positions in the homeodomain on the structure of the linker and the POU-S domain. In *Xenopus*, where loss of *Pou5f1* was followed by gene duplication, one of the three POU5F3 proteins evolved the ability to support a naïve-like pluripotency. Sequence comparisons highlighted a rapid rate of evolution and extensive divergence of the POU domain relative to other POU5F3 proteins, suggesting multiple compensatory interactions that could re-orient the two key structural motifs discussed here.

Whether the loss of one paralogue may have favoured the rise of innovations is another intriguing question. In such cases, higher concentrations of the remaining POU5 protein form could restore interactions with any co-evolved binding partner, thus compensating for a possible loss of interaction specificity and potentially also resulting in diversifications of developmental strategies. For instance, the timing and mechanism whereby PGCs segregate from somatic cells extensively vary across metazoans, and two radically different modes have been identified: pre-formation and epigenesis. The first relying on an early specification by maternal determinants, while the second depends on a later induction from surrounding tissues^64^. Intriguingly, all osteichthyans that have lost *Pou5f1* employ pre-determination, a derived trait in vertebrates (chick, *Xenopus*, sturgeon, zebrafish^64,65^), while closely related species that have retained this paralogue, such as the axolotl in amphibians, or the turtle in amniotes^66,67^, use induction. This correlation suggests that an epigenesis strategy for PGCs specification was a driving force to preserve *Pou5f1* in osteichthyans, in line with the specialisation of the protein into naïve pluripotency. This selective constraint was relaxed upon the transition to a pre-formation mode, involving an early determination of the germ line. Supporting this hypothesis, a remarkably high evolutionary rate of POU5F1 is observed in crocodilians, while the gene is lost in the bird lineage. The biological significance of the *Pou5f3* losses observed in eutherians and squamates is less clear. While mouse and human OCT4 have robust capacity to support primed and naïve pluripotency, we predict the snake POU5F1, that has robust naïve activity, would also support primed pluripotency. However, in addition to their support of primed pluripotency, the *Pou5f3* classes are expressed in the anterior hindbrain and tailbud, suggesting that in these species *Pou5f1* factors adapt to fulfil a range of developmental roles. All sarcopterygian POU5F1 proteins tested were selectively endowed with the capacity to repress spontaneous trophoblast differentiation, tracked by Cdx2 expression in ESC cultures, a property which could be mapped to the region spanning the POU-S-L and POU-HD domains. These data suggest an early emergence of the corresponding structural determinants of POU5 proteins in gnathostomes, followed by an elaboration phase taking place selectively in the POU5F1 lineage, after the gnathostome radiation. In line with this hypothesis, repression of a *Cdx* family member by POU5 proteins has been reported in *Xenopus*^38^, and *Cdx2, Pou5f1* and *Pou5f3* expression at the level of elongating posterior arms in the catshark are consistent with an ancient origin of this regulatory node (this study; ref. 68). A key innovation of mammals may have been its co-option into the developmental context of the blastocyst, regulating the trophoblast lineage commitment, as observed in the mouse^30,32^.

Pluripotency is a specific functional definition that was initially coined to describe the capacity of mammalian cells to differentiate in response to experimental manipulation and evolved to become a developmental concept describing the state or the potential of early embryonic progenitors, as compared to immortal cell lines derived from early mammalian embryos. While underlying gene regulatory networks, or more specifically pluripotency networks, have been extensively analysed in eutherian mammals, attempts to extend this notion to species outside mammals have been plagued by ambiguous sequence comparisons or non-conservation of functional activities. Despite the fundamental importance of preserving potency in early development, the extent to which key regulators of the pluripotency network have shifted during evolution has been surprising. By exploring the functional evolution of one of the fundamental regulators in the pluripotency network, we have traced the origins of an OCT4- centric network to the emergence of gnathostomes and showed that its evolution is intimately linked to the strategy used to preserve the germ line from extrinsic differentiation signals. Our work sheds light on the evolutionary forces, which drive the extensive diversification of pluripotency networks across gnathostomes, including developmental contexts, the mode of germ line specification and variations in early embryonic architecture. In conclusion, we present a highly nuanced story describing the evolution of POU5 family and suggest that phenotypic studies restricted to a single model organism can only provide a snapshot of the pluripotency network linked to this pivotal component.

## Methods

### Plasmid construction

Expression plasmids carrying *Pou5* coding sequences (CDS) were generated for ZHBTc4 ESC rescue experiment by inserting the triple flag-tagged (3xflag) *Pou5* coding sequences into pCAGIP vector^43,69^ between the CAG promoter and the *IRES-PAC* (Puromycin resistant gene encoding puromycin N-acetyl-transferase). The sources of *Pou5* genes used for the rescue assay are listed in Supplementary Data 2. *Pou5* CDS for *CpP1*, *CpP3*, *EbP5*, *LcP1*, *LcP3*, *LeP1*, *LeP3*, *MeP1*, *MeP3*, *RtP1*, *RtP3*, *ScP3* and chimeric constructs *S313*, *EbP5*^*LH2*^ and *EbP5*^*S4LH2*^ were synthesised by gBlock (IDT) and Gene synthesis (Invitrogen) services. *Xho*I/*Not*I sites were used to insert *Pou5* fragments into the pCAG 3xflag mOct4 vector in replace of the mouse *Oct4* CDS. For *LcP1*, *AmP1*, *AmP3* with *Xho*I sites present in the CDS, GeneArt® Seamless Cloning & Assembly (Invitrogen) was used to subclone the *Pou5* CDS into pUCL19 carrying a 3xflag sequence. The *3xflag Pou5* CDS were then inserted by transfer a *Xba*I/*Not*I fragment into the same sites in the pCAG vector. DNA sequencing was performed by GATC Biotech.

### Mouse ESC culture

Mouse ESCs were routinely cultured as described by ref. 38. Briefly, complete mouse ESC medium was composed of Glasgow Minimum Essential Medium (GMEM) containing 0.1 mM non-essential amino acids, 2 mM L-glutamine, 1.0 mM sodium pyruvate, 0.1 mM β–mercaptoethanol, 10% Fetal Bovine Serum (FBS) and murine LIF (homemade). The flasks/dishes (Corning) for ESC culture were coated with 0.1% gelatin in PBS. Reagents used for 2iL (N2B27, 1 μM PD0325901, 3 μM CHIR99021 and LIF on gelatin), Rosette (N2B27, 2 μM IWP2, 1 μM PD0325901 and LIF on gelatin; ref. 13) and EpiLC (N2B27, 20 ng/mL Activin 12 ng/mL bFGF and KSR (1%) on FN (16.7 μg/mL); ref. 47) culture conditions were provided in Supplementary Table 4. 2iL and Rosette cells were passaged for three times before analysis. EpiLC cells were collected after 48 h. Cell lines used include, ZHBTc4 ESCs, Oct4 null mouse embryonic stem cells carrying a tetracycline (Tc)-suppressible Oct4 transgene (ref. 27) and E14Tg2A or E14Ju (Control murine ESC lines, ref. 70 and derived in house at the Institute for Stem Cell Research, University of Edinburgh respectively). ZHBTc4 ESC cell line was gifted by Hitoshi Niwa (Institute of Molecular Embryology and Genetics, Kumamoto University).

### ZHBTc4 ESC rescue experiment

pCAGIP-POU5 expression vectors were linearised with *Sca*I or *Pvu*I. ZHBTc4 ESCs (1 × 10^7^) were electroporated with 100 μg of linearised pCAG-IP-POU5 plasmid (Gene Pulser Xcell™ Electroporation Systems at 0.8 kV, 10 μF, 0.4 mm cuvette). Electroporated cells (1 × 10^6^) were then plated onto gelatinised 100 mm culture dishes containing ESC medium with and without tetracycline (Tc, 2 μg/mL). At day 2 post electroporation, the medium was replaced with ESC medium supplemented with 1 μg/mL puromycin (with or without Tc) to select the cells expressing transfected *Pou5* genes and the medium was changed every other day thereafter. At day 9 post electroporation, several ESC colonies were big enough to be picked for expansion and used to generate stable ESC lines from both plus and minus Oct4 conditions (without and with Tc). The ESC colonies were also fixed and stained for alkaline phosphatase activity. To better elucidate the phenotypes of stable POU5-rescued lines, three clonal cell lines were characterised at passage 6, for each POU5-rescue experiment.

### iPSCs generation

To produce retrovirus particles for infecting Nanog-GFP MEF cells, packaging cell lines Plat-E were transiently transfected using Lipofectamine LTX (Invitrogen) with two expression vectors: pMXs-vector carrying gene of interest (ref. 71) and pCL-ECO containing modified gene encoding retroviral components. Retrovirus supernatant or medium containing virus particles was harvested at day 2 post transfection and concentrated by Retro-Concentrator (Clontech) solution. The titre of retrovirus was measure by Retro-X qRT-PCR Titration Kit (Clontech). For iPSC generation, transgenic mouse embryos at embryonic stage 13.5 were collected for MEF derivation. The embryos originated from the cross of male Nanog-GFP mice (a kind gift from Ian Chambers, University of Edinburgh) (age 6–10 months old) with females 129S2/ScPasCrl (Charles Reiver) (age 8 weeks old). For ethical approval, mice were maintained, bred, and manipulated at University of Copenhagen, SUND transgenic core facility authorized by the Danish National Animal Experiments Inspectorate (Dyreforsøgstilsynet, license nos. 2012-15-2934-00142 and 2013-15-2934-00935). Animal work in the Brickman lab was also authorized by the Danish National Animal Experiments Inspectorate (Dyreforsøgstilsynet, license no. 2018-15-0201-01520) and performed according to national guidelines. Nanog-GFP MEFs and feeder cells for iPSC generation were cultured in MEF medium composed of DMEM high glucose (ThermoFisher), 10% FBS (ThermoFisher), 0.1 mM non-essential amino acids (Sigma), 2 mM L-glutamine (ThermoFisher) and 0.1 mM β –mercaptoethanol (Sigma). For iPSCs induction, Nanog-GFP MEF cells were infected with ectopic retroviruses carrying Oct4 or POU5 homologue genes (X25 or X91) together with other retrovirus carrying Sox2, Klf4 and c-Myc. The infection was done at day 0 and day 1 under MEF medium. On day 3, MEF medium was replaced with defined iPSCs induction medium. On day 4, induced cells were seeded onto irradiated feeders. Medium was changed daily from day 6 to day 10 and every 2 day from day 12 onward. Infected cells and iPSCs were cultured on the irradiated feeders and in defined iPSCs induction medium composed of DMEM high glucose (ThermoFisher), 20% KnockOut Serum Replacement (ThermoFisher), 0.1 mM non-essential amino acids (Sigma), 2 mM L-glutamine (ThermoFisher), 0.1 mM β –mercaptoethanol (Sigma), LIF (homemade), 20 µg/mL Vitamin C (L-ascorbic acid, Sigma), 0.5 µM Alk5 inhibitor (A83-01, Tocris).

### Alkaline phosphatase (AP) staining

The Leucocyte alkaline phosphatase kit (Sigma-Aldrich 86R-1KT) was used for AP staining according to the manufacturer’s instructions. Briefly, cells were fixed with a fresh mixture of acetone, citrate solution and 37% formaldehyde with a ratio (8:3:1). Fixed cells were then washed twice with tap water and stained with fresh AP solution, which was generated by mixing water, FRV alkaline phosphatase solution, sodium nitrate and naphthol with a ratio (45:1:1:1). Water and naphthol were added after a 2 min incubation of FRV and sodium nitrate in the dark. About 6 mL of the staining mixture was immediately added to the 10 cm dishes with fixed cells, followed by a ~30 min incubation in the dark at room temperature. The stained cells were washed twice with tap water and air dried overnight. Images of AP colonies were acquired using a Leica-5500B microscope and then processed using Fiji ImageJ (v2.3.0/1.53 f)^72^. The stained colonies were categorised into 3 classes, undifferentiated, mixed and differentiated, based on the intensity of AP staining. The rescue index was calculated by dividing (1) the number of rescued AP positive ESC colonies obtained in the absence of endogenous Oct4 with (2) the number of colonies obtained in the presence of endogenous Oct4 for a given transfection.

### Immunofluorescence

Passage 6 POU5-rescued ESCs were seeded onto 8-well 15μ-Slide (Ibidi) at a density 20,000 cells/well. The cells were grown for 2 days and then fixed with 4% paraformaldehyde (PFA) and blocked in blocking buffer (PBS, 0.3% Triton-X and 5% donkey serum). The list of antibodies and details of their application is provided in Supplementary Table 5. Primary antibodies were diluted in antibody solution (containing PBS, 0.3% Triton-X and 1% BSA) and used to stain cells overnight at 4 °C. Cells were then stained with secondary antibodies diluted 1:800 in antibody solution for 2 h at room temperature in the dark. Cells were washed three times with PBS after each antibody incubation. Samples were imaged on a Leica AP6000 microscope and within each experiment, all images were acquired using identical acquisition settings and analysed by Fiji ImageJ (v2.3.0/1.53 f)^72^. E-cadherin (CDH1) and p120 catenin (CTNND1) were chosen as membrane-associated marker to observe cell morphology. KLF4, CDX2 and GATA6 were chosen as markers for undifferentiated naïve ESCs, trophectoderm and PrE, respectively. Immunofluorescence quantification was performed using CellProfiler v4.2.1^73^. Briefly, fluorescent images for KLF4, GATA6, CDX2 or DAPI staining of POU5-rescued cells were uploaded and run on CellProfiler software using a revised pipeline (Supplementary Note 3). The output showing the number of accepted objects indicates the number of cells with specific signals. The number of KLF4-, GATA6- or CDX2-positive cells against DAPI-positive cells (total cells in fluorescent image) were calculated as a percentage to compare between different POU5-rescued lines. Data points in the bar charts are the percentage of each biological clone.

### Western blots

Cells were washed once with PBS and then lysed directly on the plate by addition of 2x Laemmli buffer (4% (w/v) SDS, 20% (v/v) glycerol, 120 mM Tris-HCl pH 7.4). Samples were heated for 5 min at 70 °C, sonicated for 10 s at 40% power using a Sonopuls mini20 (Bandelin) and centrifuged for 10 min at 14,000 x *g* to clear the lysates. Protein concentration was determined using NanoDrop 2000 (Thermo Scientific). A sample volume of 20 µl containing 40 µg of protein, supplemented with 2 µl of 1 M DTT and 1 µl of bromophenol blue, was loaded per lane on NuPAGE 4–12% Bis-Tris Protein Gels (Invitrogen). Electrophoresis was performed in 1x NuPAGE MES SDS running buffer (Invitrogen) at 190 V for 45 min. Proteins were transferred to Nitrocellulose blotting membranes (GE Healthcare) at 400 mA for 70 min on ice in cold transfer buffer (25 mM Tris base, 190 mM Glycine, 20% Methanol). After washing in TBST (20 mM Tris (pH 7.5), 150 mM NaCl, 0.1% Tween 20), membranes were blocked for ~1 h at RT in TBST containing 10% Skim milk powder. All primary antibody incubations (overnight at 4 °C) were performed in TBST containing 5% BSA, followed by three washes in TBST and secondary antibody incubations (2 h at RT) were performed in TBST containing 5% Skim milk powder. Blots were imaged on a Chemidoc MP (Bio-Rad) and ImageLab software (version 6.1), and then quantified using Fiji ImageJ (v2.3.0/1.53 f). Loading controls were measured by cutting the membrane and blotting separately. Membranes with the same antibody were imaged together. The list of antibodies is provided in Supplementary Table 5. Uncropped and unprocessed scans are shown in Supplementary Fig. 17–18.

### Quantitative RT-PCR (qRT-PCR)

RNA and cDNA preparations were performed using the RNeasy^TM^ Mini Kit and SuperScript® III Reverse Transcriptase, respectively, according to manufacturer’s instructions. Quantitative RT-PCR was performed using the Roche Universal ProbeLibrary (UPL) System and UPL primers were designed using the Roche Assay Design Centre. All UPL primers and probes used in this study are listed in Supplementary Table 6. PCR reactions were performed using the LightCycler® 480 Probes Master Mix. Briefly, a 10 µl reaction of UPL qRT-PCR was composed of 5 μL of Probes Master Mix, 0.45 μL of 10 μM forward/left primer, 0.45 μL of 10 μM reverse/right primer, 0.1 μL of specific probe, 2 μL of diluted first strand cDNA, and 2 μL of RNase-free water. qRT-PCR data were obtained using LightCycler 480 II (Roche) and the concentration of transcripts of each gene was calculated in LightCycler 480 software (version 1.5.162 SP3) based on the cDNA pool-derived standard curve. Concentration value for each gene of interest were normalised to that of the housekeeping genes (*Tbp* and *Gapdh*) to obtain the relative transcript level.

### Microarray processing and analysis

Global gene expression profiles of POU5-rescued ESC lines were obtained using Agilent one-colour microarray-based gene expression analysis according to the manufacturer’s instructions. High quality total RNA (RNA integrity number = 10) was labelled with Cyanine 3 CTP using the Low Input Quick Amp Labelling Kit (Agilent Technologies- 5190-2305) and purified using Qiagen’s RNeasy Mini Spin Columns. The quantity of purified Cy3 labelled cRNA was measured using a Nanodrop spectrophotometer. Fragmentation was performed on 600 ng of cRNA from each sample and the fragmented cRNA was then hybridised to Agilent Mouse 8X60K slides (Grid_GenomicBuild, mm9, NCBI37, Jul2007) for 17 h at 65 °C. Hybridised slides were then washed with Agilent wash buffers and scanned on an Agilent Scanner (Agilent Technologies, G2600D SG12524268) and probe intensities were obtained by taking the gProcessedSignal from the output of Agilent feature extraction software using default settings (Agilent Feature Extraction (FE) version: 11.0.1.1). Probe annotation and statistical testing was performed using the NIA Array Analysis Tool as described in ref. 74. Significant genes were clustered and heatmap analysis was performed using Morpheus (https://software.broadinstitute.org/morpheus, ref. 75). Gene lists in each cluster were analysed for enriched Gene annotation (GO)-term for Biological Process and Cellular Components using ShinyGO v0.61^76^ and PANTHER v.16^77^ to generate lists of functional enrichment.

### Flow cytometry

ESCs were collected and stained with the indicated primary antibody dilutions Supplementary Table 5 in FACS buffer (10% FBS in PBS) for 15 min on ice. The cells were washed three times with FACS buffer and re-suspended in cold FACS buffer containing DAPI (1 μg/mL). If secondary antibodies were required, the cells were further stained with a dilution 1:800 of secondary antibodies for 15 min on ice, washed three times with PBS and re-suspended in cold FACS buffer containing DAPI. All experiments included unstained E14Tg2A or E14Ju ESCs as a non-fluorescent control that was used to establish appropriate gates. Flow cytometry was carried out on a BD LSRFortessa (BD Bioscience) with BD FACSDiva Software v6.1.3 and data analysis was performed in FCS Express v3.0 (De Novo Software). Gating strategy is described in Supplementary Fig. 19.

### Statistics and reproducibility

All POU5-rescued ESC experimental data were replicated in at least three independent experiments. We can confirm that replications of the rescue experiments were successful. For iPSCs generation, at least three independent iPSCs reprogramming experiments (different infections from the same batch of virus production) were performed. We could confirm replications of iPSCs generation were successful based on our homemade retrovirus production. At least four iPSC clonal lines from different independent iPSC inductions were analysed. At least three clones (three independent biological samples) from one or more POU5-rescue ESC experiments were used for qRT-PCR and Western blot analysis. Unpaired *t*-tests (Two-tailed) with Welch correction were used to compare independent experiments (rescue assays) and independent biological samples (qRT-PCR and Western blot analysis).

### In situ hybridisation

Catshark females were purchased from local fishermen, transported to the Banyuls sur Mer Oceanological Observatory in oxygenated sea water at 16 °C (transport authorisation n°66082) and housed in the Observatory dedicated infrastructures during the spawning season (agreement n°A6601602). They were then released in the wild by their site of collection. Whole-mount in situ hybridisations (ISH) and sections of catshark embryos were conducted using standard protocols, as described in ref. 78. Briefly, embryos were dissected from the yolk at desired stages, fixed and permeabilized prior to hybridization with digoxigenin-labelled antisense RNA probes. Hybrids were detected by immunohistochemistry using an alkaline phosphatase-conjugated antibody directed against digoxigenin in the presence of a chromogenic substrate. RNA probe sequences to detect *Pou5f1* and *Pou5f3* are provided in Supplementary Table 7.

### Structural model prediction by AlphaFold2

Protein sequences of POU5 homologues used for AlphaFold2 structural prediction^48^ are listed in Supplementary Table 1. We performed AlphaFold2 with Colab notebook (Link is noted in Supplementary Table 4). We obtained 3D coordinates, per-residue confidence metric called pLDDT and Predicted Aligned Error from each POU5 structure (shown in Supplementary Figs. 11–12). AlphaFold2 outputs include measurements of confidence per residue, termed pLDDT, on a scale from 0-100. In all POU5 models, AlphaFold2 predicted the presence of helices in the POU-specific domain (POU-S; α-helices 1–4) and in the POU homeodomain (POU-HD; α-helices 1–3), with folds and most positions being predicted with “very high” confidence (pLDDT > 90). In addition, the beginning of the linker between the POU-S and POU-HD was predicted as a helix (Linker α1'), but with variable degrees of confidence, from “confident” (90 > pLDDT > 70) to “low” confidence (70 > pLDDT > 50). The region between linker α1' and POU-HD as well as the N- and C-terminal tails were predicted with “low” to “very low” (pLDDT < 50) model confidence, suggesting that the latter are unstructured (Supplementary Figs. 11–12). From AlphaFold2 output, non-structural regions including N-/C-terminal domains and a region between α1'-helix of the linker and α1 helix of POU-HD were removed by PyMol^79^ to obtain isolated POU-S-Linker (POU-S-L) and isolated POU-HD. In PyMol, isolated domains were also superimposed to each corresponding domain in mOct4 on *PORE* sequence from the protein data bank (PDB) (3L1P, ref. 49). Isolated domains of POU5 protein and *PORE* sequence were saved to obtain new structural model on *PORE* DNA (POU5-*PORE* structure). This combined POU5-*PORE* structures were verified for the clash score (steric clashes) by Phenix^80^ using MolProbity^81^ (Supplementary Table 2). The structures with low clash score (<10) were further analysed for H-bonding interaction to *PORE* DNA using ChimeraX^82^ H-bonding prediction parameters included distance tolerance at 0.750 Å and angle tolerance at 20.000°. To compare mOct4-mSox2 polar contact predictions, an Oct4/Sox2:UTF1 structure was used (PDB 6HT5 [https://www.ncbi.nlm.nih.gov/Structure/pdb/6HT5]).

### Reporting summary

Further information on research design is available in the Nature Research Reporting Summary linked to this article.

## Supplementary information

Supplementary Information

Description of Additional Supplementary Files

Supplementary Data 1

Supplementary Data 2

Supplementary Data 3

Reporting Summary

## Data Availability

Source data for POU5 and other syntenic genes for phylogenetic analysis and evolutionary rate analysis are provided in Supplementary Data 1–2. Other published resources including Squalomix database, SkateBase, GenomeArk, Stowers Institute were used to obtain Chondrichthyes and cyclostome POU5 gene sequences. Global transcriptome data that support the findings of this study as shown in Figs. 4, 5, Supplementary Fig. 8 and Supplementary Data 3 have been deposited in GSE148167 (DNA microarray data of LcPOU5F1, LcPOU5F3 and mOct4-rescued ESCs) and GSE183049 (DNA microarray data of X91 SKM iPSCs, X25 SKM iPSCs and mOct4 SKM iPSCs). AlphaFold2-generated structural models of POU5 proteins are available from ModelArchive, individual links in Supplementary Table 1. The authors declare that all other data supporting the findings of this study are available within the paper and its supplementary information files.
