# Supplementary material for: Evolutionary origin of vertebrate OCT4/POU5 functions in supporting pluripotency
Source: Nat Commun. 2022 Sep 21;13:5537. doi: 10.1038/s41467-022-32481-z (PMC9492771; doi:10.1038/s41467-022-32481-z)
Supplement: Supplementary file 1 — Supplementary Information [file 41467_2022_32481_MOESM1_ESM.pdf]

Supplementary Information

Supplementary Figures

a

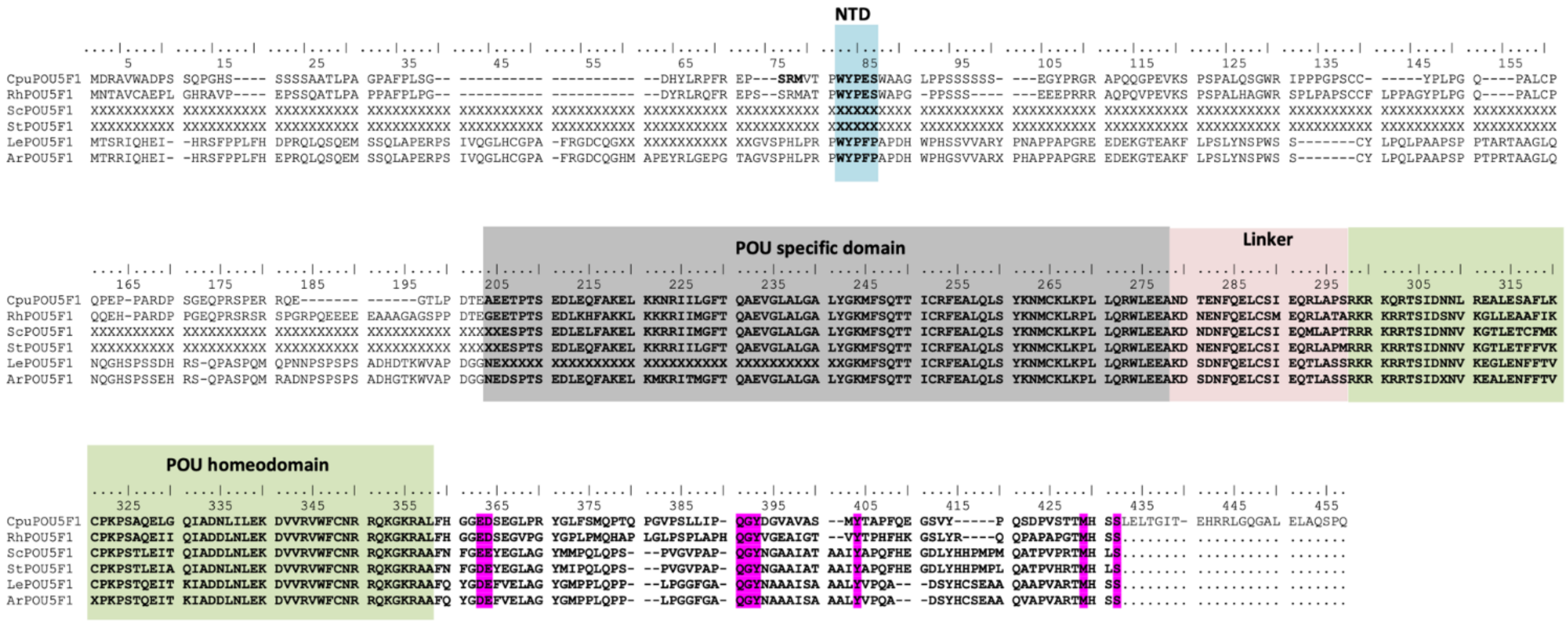

b

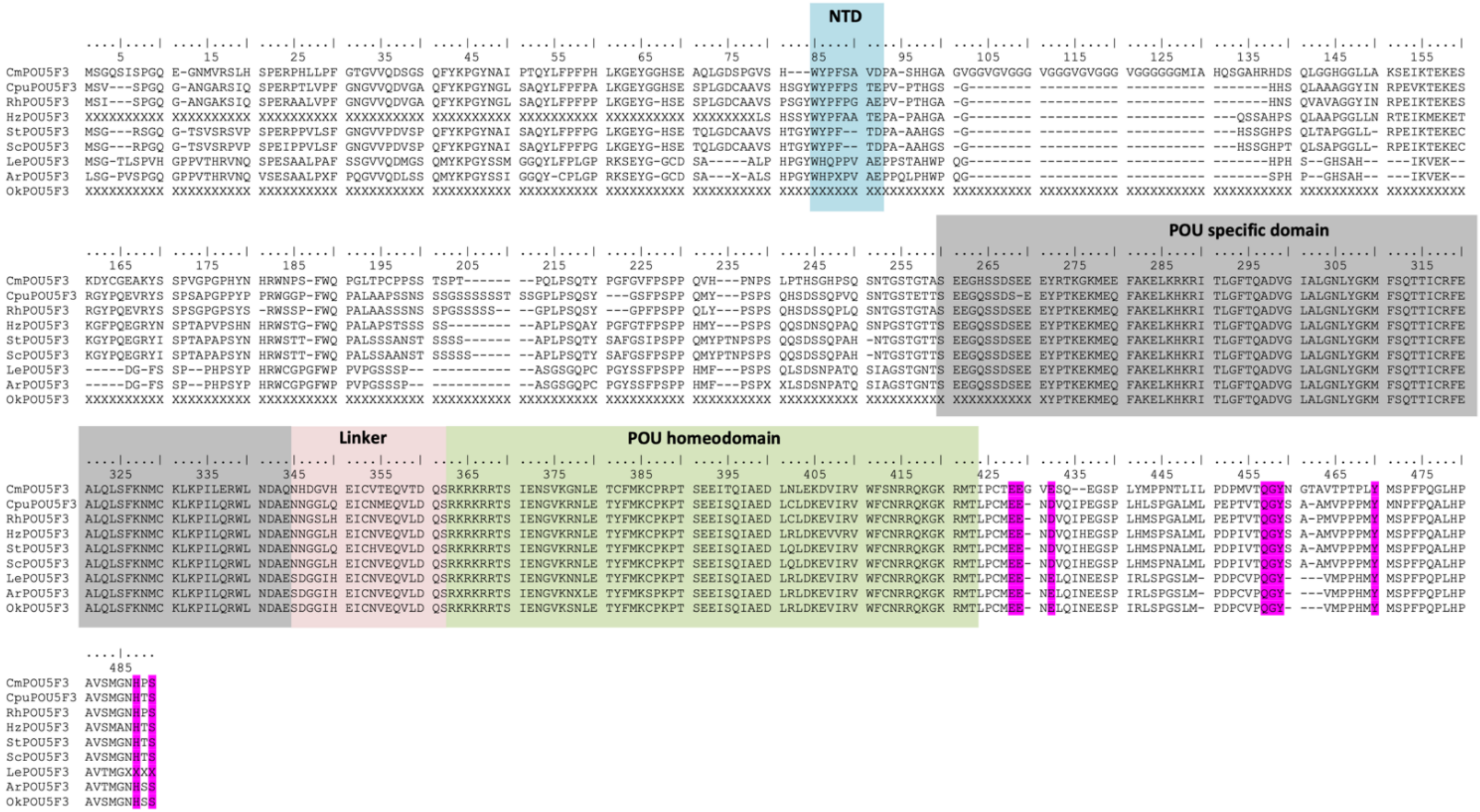
NTD
POU specific domain
Linker
POU homeodomain
5 15 25 35 45 55 65 75 85 95 105 115 125 135 145 155
CmPOU5F3 MSGQSISPGQ E-GNMVRSLH SPERPHLLPF GTGVVQDSGS QFYKPGYNAI PTQYLFPFPH LKGEYGGHSE AQLGDSPGVS H---WYPFSA VDPA-SHHGA GVGGVGVGGG VGGGVGVGGG VGGGGGGMIA HQSGAHRHDS QLGGHGGLLA KSEIKTEKES
CpuPOU5F3 MSV---SPGQ G-ANGARSIQ SPERPTLVPF GNGVVQDVGA QFYKPGYNGL SAQYLFPFPA LKGEYGGHSE SPLGDCAAVS HSGYWYPFPS TEPV-PTHGS -G-------- ---------- ---------- -------HHS QLAAAGGYIN RPEVKTEKES
RhPOU5F3 MSI---SPGQ G-ANGAKSIQ SPERAALVPF GNGVVQDVGA QFYKPGYNGL SAQYLFPFPP LKGEYG-HSE SPLGDCAAVS PSGYWYPFPG AEPV-PTHGA -G-------- ---------- ---------- -------HNS QVAVAGGYIN RPEIKTEKES
HzPOU5F3 XXXXXXXXXX XXXXXXXXXX XXXXXXXXXX XXXXXXXXXX XXXXXXXXXX XXXXXXXXXX XXXXXXXXXX XXXXXXXXLS HSSYWYPFAA TEPA-PAHGA -G-------- ---------- ---------- ---QSSAHPS QLAAPGGLLN RTEIKMEKET
StPOU5F3 MSG---RSGQ G-TSVSRSVP SPERPPVLSF GNGVVPDVSP QFYKPGYNAI SAQYLFPFPG LKGEYG-HSE TQLGDCAAVS HTGYWYPF-- TDPA-AAHGS -G-------- ---------- ---------- ---HSSGHPS QLTAPGGLL- RPEIKTEKEC
ScPOU5F3 MSG---RPGQ G-TSVSRPVP SPEIPPVLSF GNGVVPDVSP QFYKPGYNAI SAQYLFPFPG LKGEYG-HSE TQLGDCAAVS HTGYWYPF-- TDPA-AAHGS -G-------- ---------- ---------- ---HSSGHPT QLSAPGGLL- RPEIKTEKEC
LePOU5F3 MSG-TLSPVH GPPVTHRVNQ SPESAALPAF SSGVVQDMGS QMYKPGYSSM GGQYLFPLGP RKSEYG-GCD SA-----ALP HPGYWHQPPV AEPPSTAHWP QG-------- ---------- ---------- -------HPH S--GHSAH-- ---IKVEK--
ArPOU5F3 LSG-PVSPGQ GPPVTHRVNQ VSESAALPXF PQGVVQDLSS QMYKPGYSSI GGQY-CPLGP RKSEYG-GCD SA---X-ALS HPGYWHPXPV AEPPQLPHWP QG-------- ---------- ---------- -------SPH P--GHSAH-- ---IKVEK--
OkPOU5F3 XXXXXXXXXX XXXXXXXXXX XXXXXXXXXX XXXXXXXXXX XXXXXXXXXX XXXXXXXXXX XXXXXXXXXX XXXXXXXXXX XXXXXXXXXX XXXXXXXXXX XXXXXXXXXX XXXXXXXXXX XXXXXXXXXX XXXXXXXXXX XXXXXXXXXX XXXXXXXXXX
165 175 185 195 205 215 225 235 245 255 265 275 285 295 305 315
CmPOU5F3 KDYCGEAKYS SPVGPGPHYN HRWNPS-FWQ PGLTPCPPSS TSPT------ --PQLPSQTY PGFGVFPSPP QVH---PNPS LPTHSGHPSQ SNTGSTGTAS EEGHSSDSEE EYRTKGKMEE FAKELKRKRI TLGFTQADVG IALGNLYGKM FSQTTICRFE
CpuPOU5F3 RGYPQEVRYS SPSAPGPPYP PRWGGP-FWQ PALAAPSSNS SSGSSSSSST SSGPLPSQSY ---GSFPSPP QMY---PSPS QHSDSSQPVQ SNTGSTETTS EEGQSSDS-E EYPTKEKMEQ FAKELKHKRI TLGFTQADVG LALGNLYGKM FSQTTICRFE
RhPOU5F3 RGYPQEVRYS SPSGPGPSYS -RWSSP-FWQ PALAASSSNS SPGSSSSS-- --GPLPSQSY ---GPFPSPP QLY---PSPS QHSDSSQPLQ SNTGSTGTAS EEGQSSDSEE EYPTKEKMEQ FAKELKHKRI TLGFTQADVG LALGNLYGKM FSQTTICRFE
HzPOU5F3 KGFPQEGRYN SPTAPVPSHN HRWSTG-FWQ PALAPSTSSS SS-------- --APLPSQAY PGFGTFPSPP HMY---PSPS QQSDNSQPAQ SNPGSTGTTS EEGQSSDSEE EYPTKEKMEQ FAKELKHKRI TLGFTQADVG LALGNLYGKM FSQTTICRFE
StPOU5F3 KGYPQEGRYI SPTAPAPSYN HRWSTT-FWQ PALSSSANST SSSS------ --APLPSQTY SAFGSIPSPP QMYPTNPSPS QQSDSSQPAH -NTGSTGTTS EEGQSSDSEE EYPTKEKMEQ FAKELKHKRI TLGFTQADVG LALGNLYGKM FSQTTICRFE
ScPOU5F3 KGYPQEGRYI SPTAPAPSYN HRWSTT-FWQ PALSSAANST SSSSS----- --APLPSQTY SAFGSFPSPP QMYPTNPSPS QQSDSSQPAH -NTGSTGTTS EEGQSSDSEE EYPTKEKMEQ FAKELKHKRI TLGFTQADVG LALGNLYGKM FSQTTICRFE
LePOU5F3 -----DG-FS SP--PHPSYP HRWCGPGFWP PVPGSSSP-- ---------- --ASGSGQPC PGYSSFPSPP HMF---PSPS QLSDSNPATQ SIAGSTGNTS EEGQSSDSEE EYPTKEKMEQ FAKELKHKRI TLGFTQADVG LALGNLYGKM FSQTTICRFE
ArPOU5F3 -----DG-FS SP--PHPSYP HRWCGPGFWP PVPGSSSP-- ---------- --ASGSGQPC PGYSSFPSPP HMF---PSPX XLSDSNPATQ SIAGSTGNTS EEGQSSDSEE EYPTKEKMEQ FAKELKHKRI TLGFTQADVG LALGNLYGKM FSQTTICRFE
OkPOU5F3 XXXXXXXXXX XXXXXXXXXX XXXXXXXXXX XXXXXXXXXX XXXXXXXXXX XXXXXXXXXX XXXXXXXXXX XXXXXXXXXX XXXXXXXXXX XXXXXXXXXX XXXXXXXXXX XYPTKEKMEQ FAKELKHKRI TLGFTQADVG LALGNLYGKM FSQTTICRFE
325 335 345 355 365 375 385 395 405 415 425 435 445 455 465 475
CmPOU5F3 ALQLSFKNMC KLKPILERWL NDAQNHDGVH EICVTEQVTD QSRKRKRRTS IENSVKGNLE TCFMKCPRPT SEEITQIAED LNLEKDVIRV WFSNRRQKGK RMTIPCTEEG VESQ--EGSP LYMPPNTLIL PDPMVTQGYN GTAVTPTPLY MSPFPQGLHP
CpuPOU5F3 ALQLSFKNMC KLKPILQRWL NDAENNGGLQ EICNMEQVLD QSRKRKRRTS IENGVKRNLE TYFMKCPKPT SEEISQIAED LCLDKEVIRV WFCNRRQKGK RMTLPCMEE- NDVQIPEGSP LHLSPGALML PEPTVTQGYS A-AMVPPPMY MNPFPQALHP
RhPOU5F3 ALQLSFKNMC KLKPILQRWL NDAENNGSLH EICNVEQVLD QSRKRKRRTS IENGVKRNLE TYFMKCPKPT SEEISQIAED LCLDKEVIRV WFCNRRQKGK RMTLPCMEE- NDVQIPEGSP LHMSPGALML PEPTVTQGYS A-PMVPPPMY MSPFPQALHP
HzPOU5F3 ALQLSFKNMC KLKPILQRWL NDAENNGGLH EICNVEQVLD QSRKRKRRTS IENGVKRNLE TYFMKCPKPT SEEISQIAED LRLDKEVVRV WFCNRRQKGK RMTLPCMEE- NDVQIHEGSP LHMSPSALML PDPIVTQGYS A-AMVPPPMY MSPFPQALHP
StPOU5F3 ALQLSFKNMC KLKPILQRWL NDAENNGGLQ EICHVEQVLD QSRKRKRRTS IENGVKRNLE TYFMKCPKPT SEEISQIAED LQLDKEVIRV WFCNRRQKGK RMTLPCMEE- NDVQIHEGSP LHMSPNALML PDPIVTQGYS A-AMVPPPMY MSPFPQALHP
ScPOU5F3 ALQLSFKNMC KLKPILQRWL NDAENNGGLH EICNVEQVLD QSRKRKRRTS IENGVKRNLE TYFMKCPKPT SEEISQIAED LQLDKEVIRV WFCNRRQKGK RMTLPCMEE- NDVQIHEGSP LHMSPNALML PDPIVTQGYS A-AMVPPPMY MSPFPQALHP
LePOU5F3 ALQLSFKNMC KLKPILQRWL NDAESDGGIH EICNVEQVLD QSRKRKRRTS IENGVKNNLE TYFMKCPKPT SEEISQIAED LRLDKEVIRV WFCNRRQKGK RMTLPCMEE- NELQINEESP IRLSPGSLM- PDPCVPQGY- ---VMPPHMY MSPFPQPLHP
ArPOU5F3 ALQLSFKNMC KLKPILQRWL NDAESDGGIH EICNVEQVLD QSRXRKRRTS IENGVKNXLE TYFMKSPKPT SEEISQIAED LRLDKEVIRV WFCNRRQKGK RMTLPCMEE- NELQINEESP IRLSPGSLM- PDPCVPQGY- ---VMPPHMY MSPFPQPLHP
OkPOU5F3 ALQLSFKNMC KLKPILQRWL NDAESDGGIH EICNVEQVLD QSRKRKRRTS IENGVKSNLE TYFMKCPKPT SEEISQIAED LRLDKEVIRV WFCNRRQKGK RMTLPCMEE- NELQINEESP IRLSPGSLM- PDPCVPQGY- ---VMPPHMY MSPFPQPLHP
485
CmPOU5F3 AVSMGNHPS
CpuPOU5F3 AVSMGNHTS
RhPOU5F3 AVSMGNHPS
HzPOU5F3 AVSMANHTS
StPOU5F3 AVSMGNHTS
ScPOU5F3 AVSMGNHTS
LePOU5F3 AVTMGXXXX
ArPOU5F3 AVTMGNHSS
OkPOU5F3 AVSMGNHSS

c

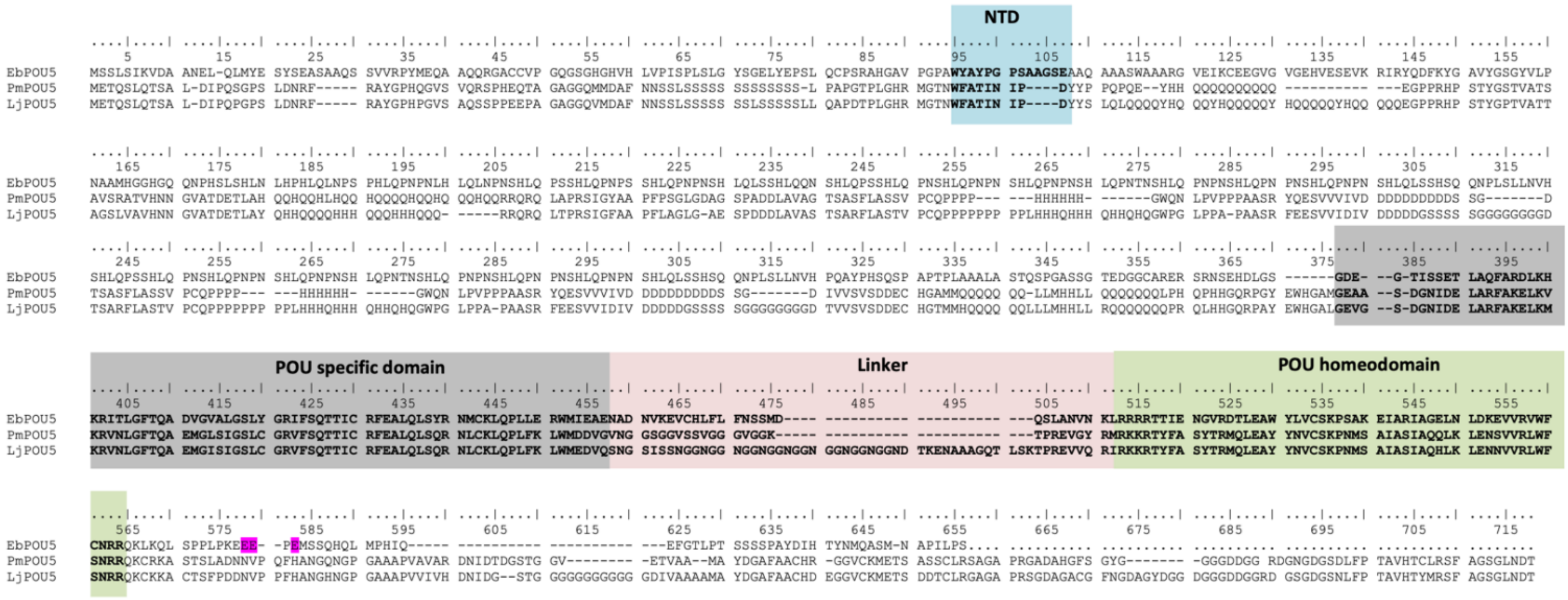
NTD
POU specific domain
Linker
POU homeodomain
EbPOU5
PmPOU5
LjPOU5

d

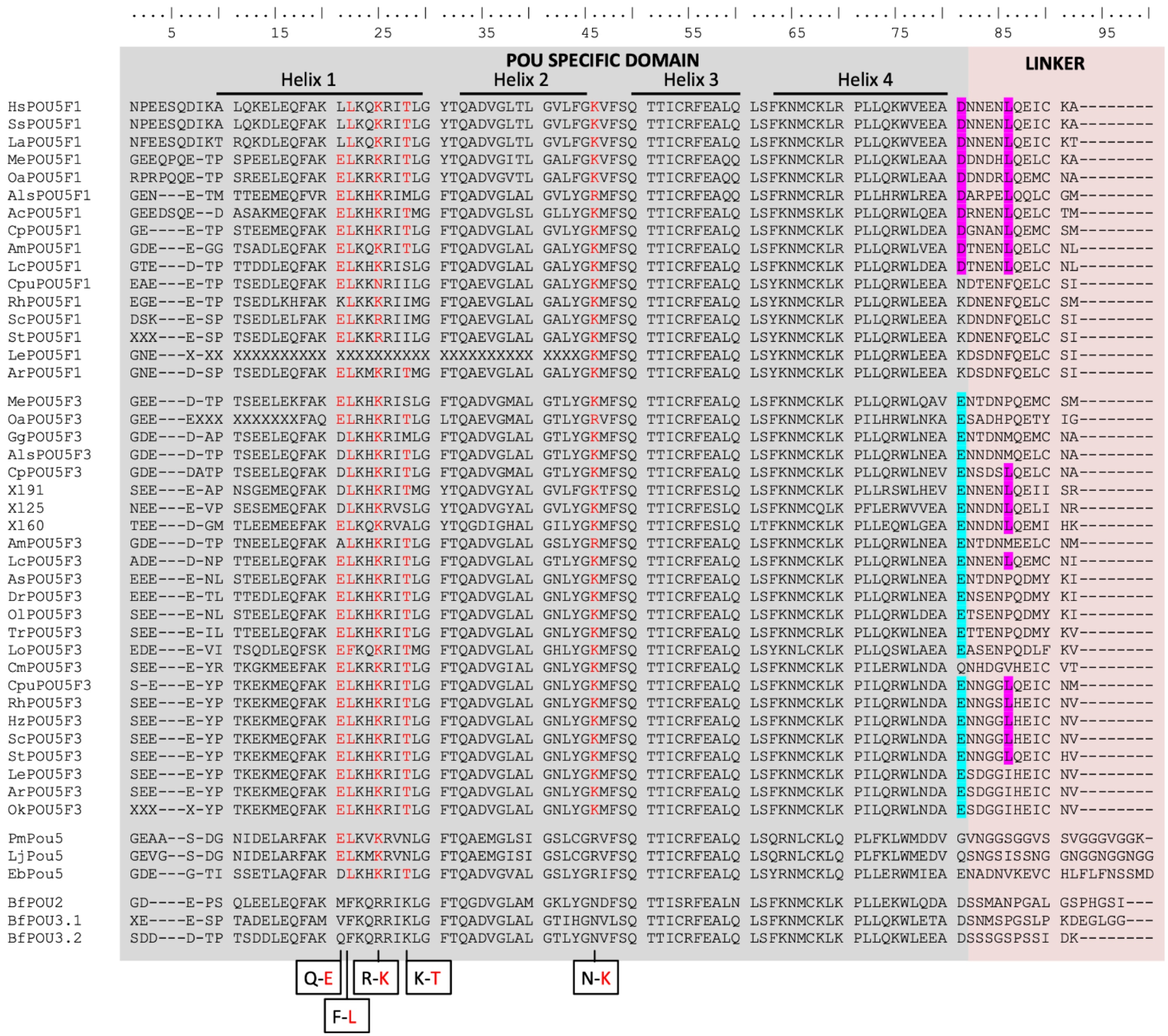
5 15 25 35 45 55 65 75 85 95
POU SPECIFIC DOMAIN
LINKER
Helix 1
Helix 2
Helix 3
Helix 4
HsPOU5F1 NPEESQDIKA LQKELEQFAK LLKQKRITLG YTQADVGLTL GVLFGKVFSQ TTICRFEALQ LSFKNMCKLR PLLQKWVEEA DNNENLQEIC KA--------
SsPOU5F1 NPEESQDIKA LQKDLEQFAK LLKQKRITLG YTQADVGLTL GVLFGKVFSQ TTICRFEALQ LSFKNMCKLR PLLQKWVEEA DNNENLQEIC KA--------
LaPOU5F1 NFEESQDIKT RQKDLEQFAK LLKQKRITLG YTQADVGLTL GVLFGKVFSQ TTICRFEALQ LSFKNMCKLR PLLQKWVEEA DNNENLQEIC KT--------
MePOU5F1 GEEQPQE-TP SPEELEQFAK ELKRKRITLG YTQADVGITL GALFGKVFSQ TTICRFEAQQ LSFKNMCKLR PLLQKWLEAA DDNDHLQELC KA--------
OaPOU5F1 RPRPQQE-TP SREELEQFAK ELKRKRITLG YTQADVGVTL GALFGKVFSQ TTICRFEAQQ LSFKNMCKLR PLLQRWLEAA DDNDRLQEMC NA--------
AlsPOU5F1 GEN---E-TM TTEEMEQFVR ELKHKRIMLG FTQADVGLAL GVLYGRMFSQ TTICRFEAQQ LSFRNMCRLR PLLHRWLREA DARPELQQLC GM--------
AcPOU5F1 GEEDSQE--D ASAKMEQFAK ELKHKRITMG FTQADVGLSL GLLYGKMFSQ TTICRFEALQ LSFKNMSKLK PLLQRWLQEA DRNENLQELC TM--------
CpPOU5F1 GE----E-TP STEEMEQFAK ELKHKRITLG FTQADVGLAL GVLYGKMFSQ TTICRFEALQ LSFKNMCKLK PLLQRWLDEA DGNANLQEMC SM--------
AmPOU5F1 GDE---E-GG TSADLEQFAK ELKQKRITLG FTQADVGLAL GALYGKMFSQ TTICRFEALQ LSFKNMCKLR PLLQRWLVEA DTNENLQELC NL--------
LcPOU5F1 GTE---D-TP TTDDLEQFAK ELKHKRISLG FTQADVGLAL GALYGKMFSQ TTICRFEALQ LSFKNMCKLK PLLQRWLDEA DTNENLQELC NL--------
CpuPOU5F1 EAE---E-TP TSEDLEQFAK ELKKNRIILG FTQAEVGLAL GALYGKMFSQ TTICRFEALQ LSYKNMCKLK PLLQRWLEEA NDTENFQELC SI--------
RhPOU5F1 EGE---E-TP TSEDLKHFAK KLKKKRIIMG FTQAEVGLAL GALYGKMFSQ TTICRFEALQ LSYKNMCKLK PLLQRWLEEA KDNENFQELC SM--------
ScPOU5F1 DSK---E-SP TSEDLELFAK ELKKRRIIMG FTQAEVGLAL GALYGKMFSQ TTICRFEALQ LSYKNMCKLK PLLQRWLEEA KDNDNFQELC SI--------
StPOU5F1 XXX---E-SP TSEDLEQFAK ELKKRRIILG FTQAEVGLAL GALYGKMFSQ TTICRFEALQ LSYKNMCKLK PLLQRWLEEA KDNENFQELC SI--------
LePOU5F1 GNE---X-XX XXXXXXXXXX XXXXXXXXXX XXXXXXXXXX XXXXGKMFSQ TTICRFEALQ LSYKNMCKLK PLLQRWLEEA KDSDNFQELC SI--------
ArPOU5F1 GNE---D-SP TSEDLEQFAK ELKMKRITMG FTQAEVGLAL GALYGKMFSQ TTICRFEALQ LSYKNMCKLK PLLQRWLEEA KDSDNFQELC SI--------
MePOU5F3 GEE---D-TP TSEELEKFAK ELKHKRISLG FTQADVGMAL GTLYGKMFSQ TTICRFEALQ LSFKNMCKLK PLLQRWLQAV ENTDNPQEMC SM--------
OaPOU5F3 GEE---EXXX XXXXXXXFAQ ELRHKRITLG LTQAEVGMAL GTLYGRVFSQ TTICRFEALQ LSFKNMCKLK PILHRWLNKA ESADHPQETY IG--------
GgPOU5F3 GDE---D-AP TSEELEQFAK DLKHKRIMLG FTQADVGLAL GTLYGKMFSQ TTICRFEALQ LSFKNMCKLK PLLQRWLNEA ENTDNMQEMC NA--------
AlsPOU5F3 GDE---D-TP TSEELEQFAK DLKHKRITLG FTQADVGLAL GTLYGKMFSQ TTICRFEALQ LSFKNMCKLK PLLQRWLNEA ENNDNMQELC NA--------
CpPOU5F3 GDE---DATP TSEELEQFAK DLKHKRITLG FTQADVGMAL GTLYGKMFSQ TTICRFEALQ LSFKNMCKLK PLLQRWLNEV ENSDSLQELC NA--------
X191 SEE---E-AP NSGEMEQFAK DLKHKRITMG YTQADVGYAL GVLFGKTFSQ TTICRFESLQ LSFKNMCKLK PLLRSWLHEV ENNENLQEII SR--------
X125 NEE---E-VP SESEMEQFAK DLKHKRVSLG YTQADVGYAL GVLYGKMFSQ TTICRFESLQ LSFKNMCQLK PFLERWVVEA ENNDNLQELI NR--------
X160 TEE---D-GM TLEEMEEFAK ELKQKRVALG YTQGDIGHAL GILYGKMFSQ TTICRFESLQ LTFKNMCKLK PLLEQWLGEA ENNDNLQEMI HK--------
AmPOU5F3 GDE---D-TP TNEELEQFAK ALKHKRITLG FTQADVGMAL GSLYGKMFSQ TTICRFEALQ LSFKNMCKLK PLLQRWLNEA ENTDNMEELC NM--------
LcPOU5F3 ADE---D-NP TTEELEQFAK ELKHKRITLG FTQADVGLAL GTLYGKMFSQ TTICRFEALQ LSFKNMCKLK PLLQRWLNEA ENNENLQEMC NI--------
AsPOU5F3 EEE---E-NL STEELEQFAK ELKHKRITLG FTQADVGLAL GNLYGKMFSQ TTICRFEALQ LSFKNMCKLK PLLQRWLNEA ENTDNPQDMY KI--------
DrPOU5F3 EEE---E-TL TTEDLEQFAK ELKHKRITLG FTQADVGLAL GNLYGKMFSQ TTICRFEALQ LSFKNMCKLK PLLQRWLNEA ENSENPQDMY KI--------
OlPOU5F3 SEE---E-NL STEELEQFAK ELKHKRITLG FTQADVGLAL GNLYGKMFSQ TTICRFEALQ LSFKNMCKLK PLLQRWLDEA ETSENPQDMY KI--------
TrPOU5F3 SEE---E-IL TTEELEQFAK ELKHKRITLG FTQADVGLAL GNLYGKMFSQ TTICRFEALQ LSFKNMCRLK PLLQKWLNEA ETTENPQDMY KV--------
LoPOU5F3 EDE---E-VI TSQDLEQFSK EFKQKRITMG FTQADVGLAL GHLYGKMFSQ TTICRFEALQ LSYKNLCKLK PLLQSWLAEA EASENPQDLF KV--------
CmPOU5F3 SEE---E-YR TKGKMEEFAK ELKRKRITLG FTQADVGIAL GNLYGKMFSQ TTICRFEALQ LSFKNMCKLK PILERWLNDA QNHDGVHEIC VT--------
CpuPOU5F3 S-E---E-YP TKEKMEQFAK ELKHKRITLG FTQADVGLAL GNLYGKMFSQ TTICRFEALQ LSFKNMCKLK PILQRWLNDA ENNGGLQEIC NM--------
RhPOU5F3 SEE---E-YP TKEKMEQFAK ELKHKRITLG FTQADVGLAL GNLYGKMFSQ TTICRFEALQ LSFKNMCKLK PILQRWLNDA ENNGSLHEIC NV--------
HzPOU5F3 SEE---E-YP TKEKMEQFAK ELKHKRITLG FTQADVGLAL GNLYGKMFSQ TTICRFEALQ LSFKNMCKLK PILQRWLNDA ENNGGLHEIC NV--------
ScPOU5F3 SEE---E-YP TKEKMEQFAK ELKHKRITLG FTQADVGLAL GNLYGKMFSQ TTICRFEALQ LSFKNMCKLK PILQRWLNDA ENNGGLHEIC NV--------
StPOU5F3 SEE---E-YP TKEKMEQFAK ELKHKRITLG FTQADVGLAL GNLYGKMFSQ TTICRFEALQ LSFKNMCKLK PILQRWLNDA ENNGGLQEIC HV--------
LePOU5F3 SEE---E-YP TKEKMEQFAK ELKHKRITLG FTQADVGLAL GNLYGKMFSQ TTICRFEALQ LSFKNMCKLK PILQRWLNDA ESDGGIHEIC NV--------
ArPOU5F3 SEE---E-YP TKEKMEQFAK ELKHKRITLG FTQADVGLAL GNLYGKMFSQ TTICRFEALQ LSFKNMCKLK PILQRWLNDA ESDGGIHEIC NV--------
OkPOU5F3 XXX---X-YP TKEKMEQFAK ELKHKRITLG FTQADVGLAL GNLYGKMFSQ TTICRFEALQ LSFKNMCKLK PILQRWLNDA ESDGGIHEIC NV--------
PmPou5 GEAA--S-DG NIDELARFAK ELKVKRVNLG FTQAEMGLSI GSLCGRVFSQ TTICRFEALQ LSQRNLCKLQ PLFKLWMDDV GVNGGSGGVS SVGGGVGGK-
LjPou5 GEVG--S-DG NIDELARFAK ELKMKRVNLG FTQAEMGISI GSLCGRVFSQ TTICRFEALQ LSQRNLCKLQ PLFKLWMEDV QSNGSISSNG GNGGNGGNGG
EbPou5 GDE---G-TI SSETLAQFAR DLKHKRITLG FTQADVGVAL GSLYGRIFSQ TTICRFEALQ LSYRNMCKLQ PLLERWMIEA ENADNVKEVC HLFLFNSSMD
BfPOU2 GD----E-PS QLEELEQFAK MFKQRRIKLG FTQGDVGLAM GKLYGNDFSQ TTISRFEALN LSFKNMCKLK PLLEKWLQDA DSSMANPGAL GSPHGSI---
BfPOU3.1 XE----E-SP TADELEQFAM VFKQRRIKLG FTQADVGLAL GTIHGNVLSQ TTICRFEALQ LSFKNMCKLK PLLQKWLETA DSNMSPGSLP KDEGLGG---
BfPOU3.2 SDD---D-TP TSDDLEQFAK QFKQRRIKLG FTQADVGLAL GTLYGNVFSQ TTICRFEALQ LSFKNMCKLK PLLQKWLEEA DSSSGSPSSI DK--------
Q-E
R-K
K-T
N-K
F-L

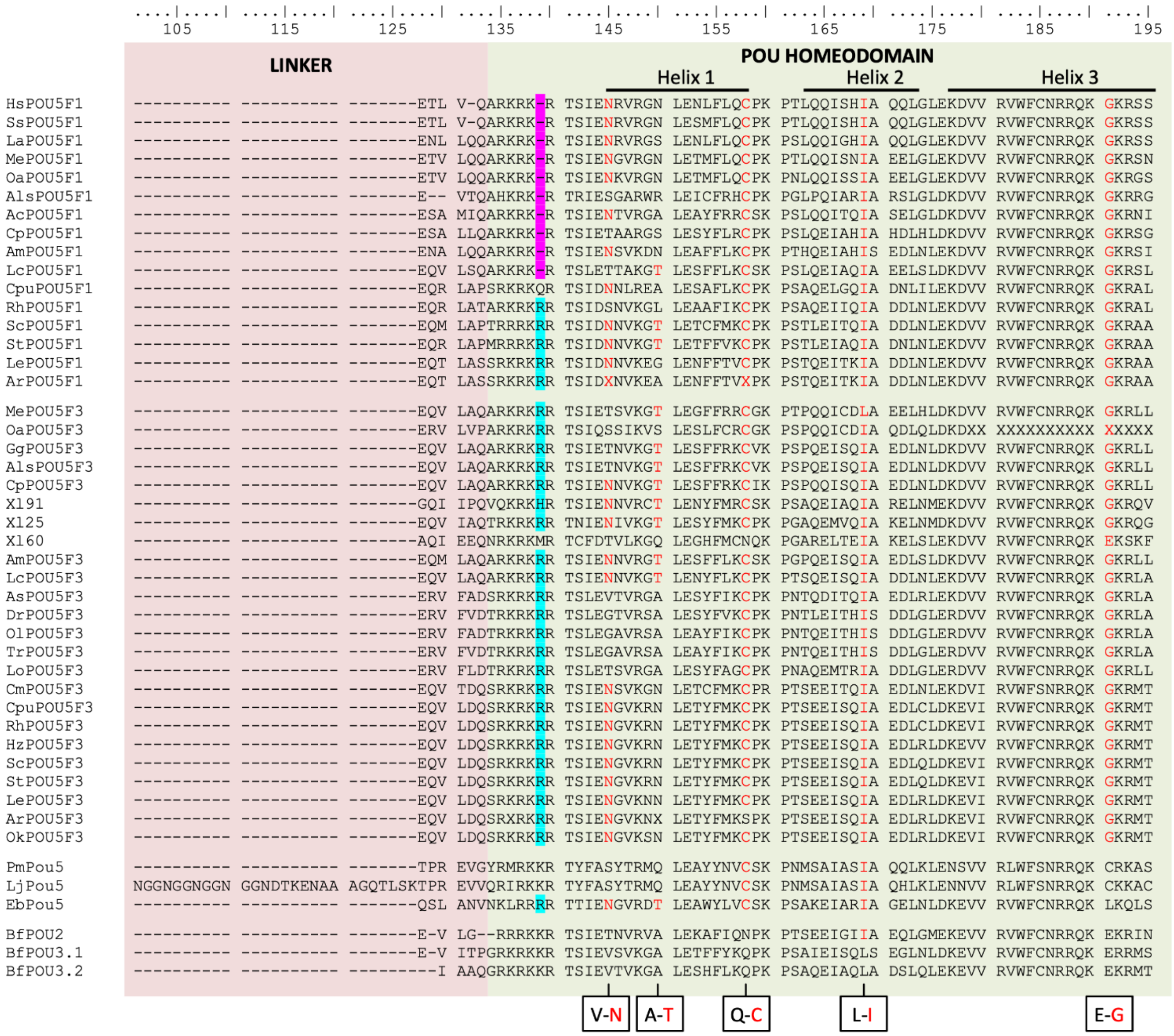

105 115 125 135 145 155 165 175 185 195
LINKER
POU HOMEODOMAIN
Helix 1
Helix 2
Helix 3
HsPOU5F1 ---------- ---------- -------ETL V-QARKRK-R TSIENRVRGN LENLFLQCPK PTLQQISHIA QQLGLEKDVV RVWFCNRRQK GKRSS
SsPOU5F1 ---------- ---------- -------ETL V-QARKRK-R TSIENRVRGN LESMFLQCPK PTLQQISHIA QQLGLEKDVV RVWFCNRRQK GKRSS
LaPOU5F1 ---------- ---------- -------ENL LQQARKRK-R TSIENRVRGS LENLFLQCPK PSLQQIGHIA QQLGLEKDVV RVWFCNRRQK GKRSS
MePOU5F1 ---------- ---------- -------ETV LQQARKRK-R TSIENGVRGN LETMFLQCPK PTLQQISNIA EELGLEKDVV RVWFCNRRQK GKRSN
OaPOU5F1 ---------- ---------- -------ETV LQQARKRK-R TSIENKVRGN LETMFLQCPK PNLQQISSIA EELGLEKDVV RVWFCNRRQK GKRGS
AlsPOU5F1 ---------- ---------- -------E-- VTQAHKRK-R TRIESGARWR LEICFRHCPK PGLPQIARIA RSLGLDKDVV RVWFCNRRQK GKRRG
AcPOU5F1 ---------- ---------- -------ESA MIQARKRK-R TSIENTVRGA LEAYFRRCSK PSLQQITQIA SELGLDKDVV RVWFCNRRQK GKRNI
CpPOU5F1 ---------- ---------- -------ESA LLQARKRK-R TSIETAARGS LESYFLRCPK PSLQEIAHIA HDLHLDKDVV RVWFCNRRQK GKRSG
AmPOU5F1 ---------- ---------- -------ENA LQQARKRK-R TSIENSVKDN LEAFFLKCPK PTHQEIAHIS EDLNLEKDVV RVWFCNRRQK GKRSI
LcPOU5F1 ---------- ---------- -------EQV LSQARKRK-R TSLETTAKGT LESFFLKCSK PSLQEIAQIA EELSLDKDVV RVWFCNRRQK GKRSL
CpuPOU5F1 ---------- ---------- -------EQR LAPSRKRKQR TSIDNNLREA LESAFLKCPK PSAQELGQIA DNLILEKDVV RVWFCNRRQK GKRAL
RhPOU5F1 ---------- ---------- -------EQR LATARKRKRR TSIDSNVKGL LEAAFIKCPK PSAQEIIQIA DDLNLEKDVV RVWFCNRRQK GKRAL
ScPOU5F1 ---------- ---------- -------EQM LAPTRRRKRR TSIDNNVKGT LETCFMKCPK PSTLEITQIA DDLNLEKDVV RVWFCNRRQK GKRAA
StPOU5F1 ---------- ---------- -------EQR LAPMRRRKRR TSIDNNVKGT LETFFVKCPK PSTLEIAQIA DNLNLEKDVV RVWFCNRRQK GKRAA
LePOU5F1 ---------- ---------- -------EQT LASSRKRKRR TSIDNNVKEG LENFFTVCPK PSTQEITKIA DDLNLEKDVV RVWFCNRRQK GKRAA
ArPOU5F1 ---------- ---------- -------EQT LASSRKRKRR TSIDXNVKEA LENFFTVXPK PSTQEITKIA DDLNLEKDVV RVWFCNRRQK GKRAA
MePOU5F3 ---------- ---------- -------EQV LAQARKRKRR TSIETSVKGT LEGFFRRCGK PTPQQICDLA EELHLDKDVV RVWFCNRRQK GKRLL
OaPOU5F3 ---------- ---------- -------ERV LVPARKRKRR TSIQSSIKVS LESLFCRCGK PSPQQICDIA QDLQLDKDXX XXXXXXXXXX XXXXX
GgPOU5F3 ---------- ---------- -------EQV LAQARKRKRR TSIETNVKGT LESFFRKCVK PSPQEISQIA EDLNLDKDVV RVWFCNRRQK GKRLL
AlsPOU5F3 ---------- ---------- -------EQV LAQARKRKRR TSIETNVKGT LESFFRKCVK PSPQEISQIA EDLNLDKDVV RVWFCNRRQK GKRLL
CpPOU5F3 ---------- ---------- -------EQV LAQARKRKRR TSIENNVKGT LESFFRKCIK PSPQQISQIA EDLNLDKDVV RVWFCNRRQK GKRLL
X191 ---------- ---------- -------GQI IPQVQKRKHR TSIENNVRCT LENYFMRCSK PSAQEIAQIA RELNMEKDVV RVWFCNRRQK GKRQV
X125 ---------- ---------- -------EQV IAQTRKRKRR TNIENIVKGT LESYFMKCPK PGAQEMVQIA KELNMDKDVV RVWFCNRRQK GKRQG
X160 ---------- ---------- -------AQI EEQNRKRKMR TCFDTVLKGQ LEGHFMCNQK PGARELTEIA KELSLEKDVV RVWFCNRRQK EKSKF
AmPOU5F3 ---------- ---------- -------EQM LAQARKRKRR TSIENNVRGT LESFFLKCSK PGPQEISQIA EDLSLDKDVV RVWFCNRRQK GKRLL
LcPOU5F3 ---------- ---------- -------EQV LAQARKRKRR TSIENNVKGT LENYFLKCPK PTSQEISQIA DDLNLEKDVV RVWFCNRRQK GKRLA
AsPOU5F3 ---------- ---------- -------ERV FADSRKRKRR TSLEVTVRGA LESYFIKCPK PNTQDITQIA EDLRLEKDVV RVWFCNRRQK GKRLA
DrPOU5F3 ---------- ---------- -------ERV FVDTRKRKRR TSLEGTVRSA LESYFVKCPK PNTLEITHIS DDLGLERDVV RVWFCNRRQK GKRLA
OlPOU5F3 ---------- ---------- -------ERV FADTRKRKRR TSLEGAVRSA LEAYFIKCPK PNTQEITHIS DDLGLERDVV RVWFCNRRQK GKRLA
TrPOU5F3 ---------- ---------- -------ERV FVDTRKRKRR TSLEGAVRSA LEAYFIKCPK PNTQEITHIS DDLGLERDVV RVWFCNRRQK GKRLA
LoPOU5F3 ---------- ---------- -------ERV FLDTRKRKRR TSLETSVRGA LESYFAGCPK PNAQEMTRIA DDLGLERDVV RVWFCNRRQK GKRLL
CmPOU5F3 ---------- ---------- -------EQV TDQSRKRKRR TSIENSVKGN LETCFMKCPR PTSEEITQIA EDLNLEKDVI RVWFSNRRQK GKRMT
CpuPOU5F3 ---------- ---------- -------EQV LDQSRKRKRR TSIENGVKRN LETYFMKCPK PTSEEISQIA EDLCLDKEVI RVWFCNRRQK GKRMT
RhPOU5F3 ---------- ---------- -------EQV LDQSRKRKRR TSIENGVKRN LETYFMKCPK PTSEEISQIA EDLCLDKEVI RVWFCNRRQK GKRMT
HzPOU5F3 ---------- ---------- -------EQV LDQSRKRKRR TSIENGVKRN LETYFMKCPK PTSEEISQIA EDLRLDKEVV RVWFCNRRQK GKRMT
ScPOU5F3 ---------- ---------- -------EQV LDQSRKRKRR TSIENGVKRN LETYFMKCPK PTSEEISQIA EDLQLDKEVI RVWFCNRRQK GKRMT
StPOU5F3 ---------- ---------- -------EQV LDQSRKRKRR TSIENGVKRN LETYFMKCPK PTSEEISQIA EDLQLDKEVI RVWFCNRRQK GKRMT
LePOU5F3 ---------- ---------- -------EQV LDQSRKRKRR TSIENGVKNN LETYFMKCPK PTSEEISQIA EDLRLDKEVI RVWFCNRRQK GKRMT
ArPOU5F3 ---------- ---------- -------EQV LDQSRXRKRR TSIENGVKNX LETYFMKSPK PTSEEISQIA EDLRLDKEVI RVWFCNRRQK GKRMT
OkPOU5F3 ---------- ---------- -------EQV LDQSRKRKRR TSIENGVKSN LETYFMKCPK PTSEEISQIA EDLRLDKEVI RVWFCNRRQK GKRMT
PmPou5 ---------- ---------- -------TPR EVGYRMRKKR TYFASYTRMQ LEAYYNVCSK PNMSAIASIA QQLKLENSVV RLWFSNRRQK CRKAS
LjPou5 NGGNGGNGGN GGNDTKENAA AGQTLSKTPR EVVQRIRKKR TYFASYTRMQ LEAYYNVCSK PNMSAIASIA QHLKLENNVV RLWFSNRRQK CKKAC
EbPou5 ---------- ---------- -------QSL ANVNKLRRRR TTIENGVRDT LEAWYLVCSK PSAKEIARIA GELNLDKEVV RVWFCNRRQK LKQLS
BfPOU2 ---------- ---------- -------E-V LG--RRRKKR TSIETNVRVA LEKAFIQNPK PTSEEIGIIA EQLGMEKEVV RVWFCNRRQK EKRIN
BfPOU3.1 ---------- ---------- -------E-V ITPGRKRKKR TSIEVSVKGA LETFFYKQPK PSAIEISQLS EGLNLDKEVV RVWFCNRRQK ERRMS
BfPOU3.2 ---------- ---------- ---------I AAQGRKRKKR TSIEVTVKGA LESHFLKQPK PSAQEIAQLA DSLQLEKEVV RVWFCNRRQK EKRMT
V-N
A-T
Q-C
L-I
E-G

Supplementary Fig. 1, related to Fig. 1a. Alignment of POU5 predicted sequences: (a) and (b) chondrichthyan full-length POU5F1 and POU5F3 respectively, (c) cyclostome full-length POU5 and (d) vertebrate POU5 over the POU domain, linker and homeodomain. Amphioxus (*Branchiostoma floridae*) POU2 and POU3 are included as outgroups for comparisons in (d). A dot denotes a missing amino acid, an X undetermined sequence. In (d), POU5 synapomorphies, as identified in ref.1, are boxed and shown in red, with the ancestral residue in black. The N-terminal conserved domain (NTD), POU specific domain and POU homeodomain are shown respectively in blue, grey and green. Residues conserved between gnathostome POU5F1 and POU5F3 but not with cyclostome POU5 in the C-terminal part of the protein are shaded in magenta in (a-c) and sequences, which can be aligned between both classes, are in bold characters. *Amblyraja radiata* sequences were retrieved from a genome assembly, which does not meet VGP quality standard and were edited to remove putative frame shifts in the sequence (see Supplementary Data 1). Species abbreviations: Ar, *Amblyraja radiata*; Cm, *Callorhinchus milii*; Cpu, *Chyloscyllium punctatum*; Hz, *Heterodontus zebra*; Le, *Leucoraja erinacea*; Ok, *Okamejei kenojei*; Rh/Rt, *Rhincodon typus*; Sc, *Scyliorhinus canicula*; St, *Scyliorhinus torazame*.

a Gnathostome *POU5F1*

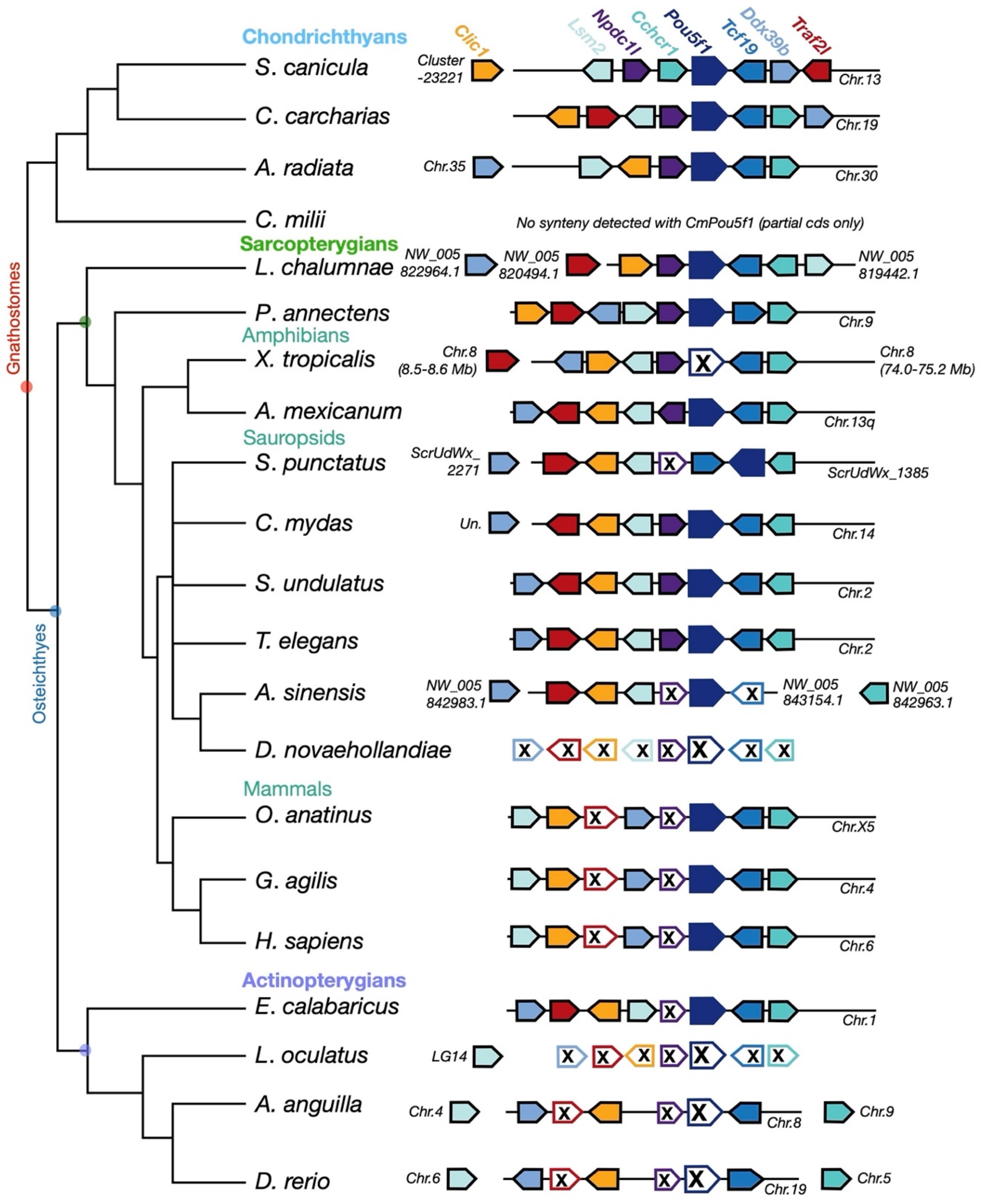

b **Gnathostome *POU5F3***

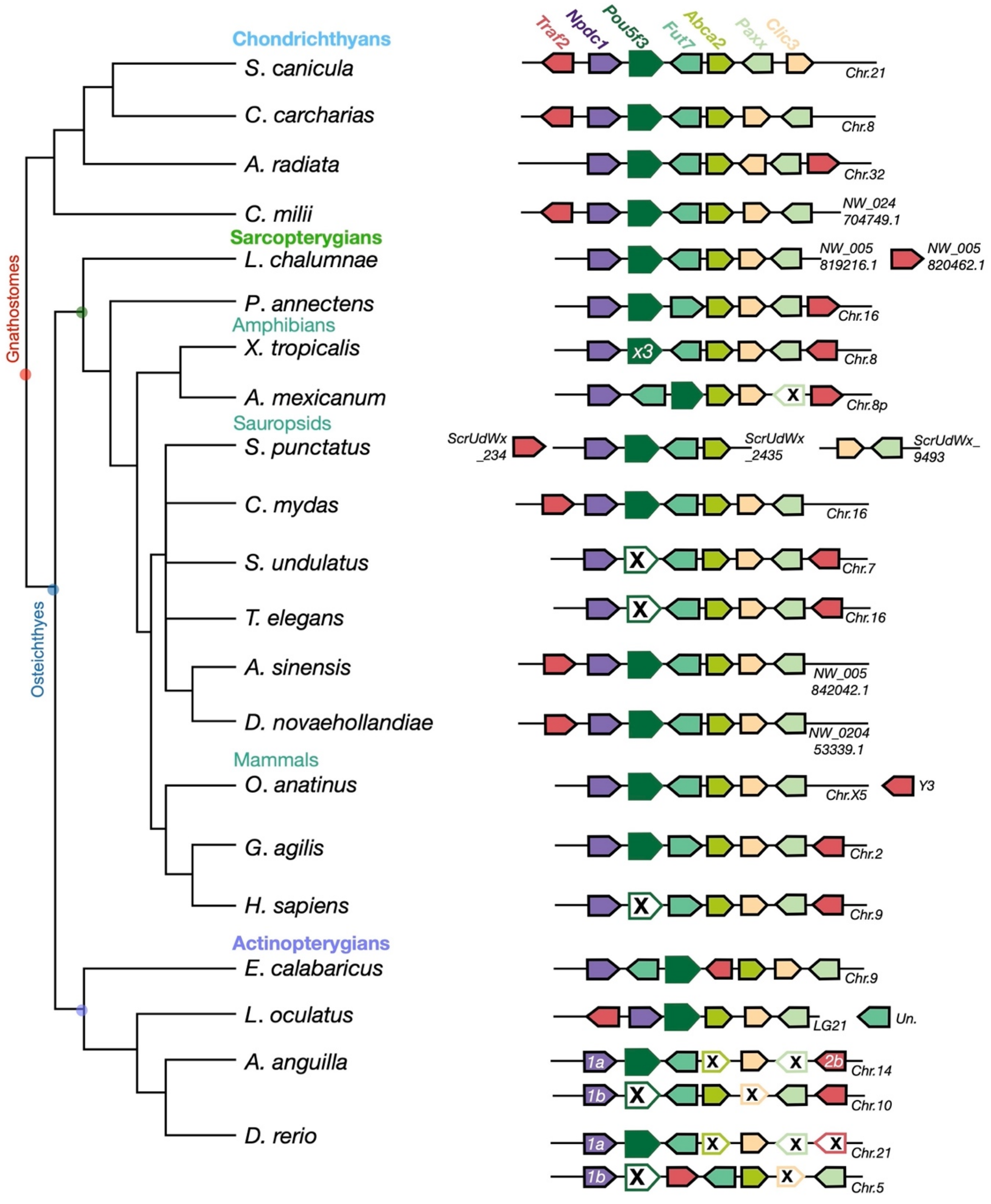

c **Cyclostome *POU5***

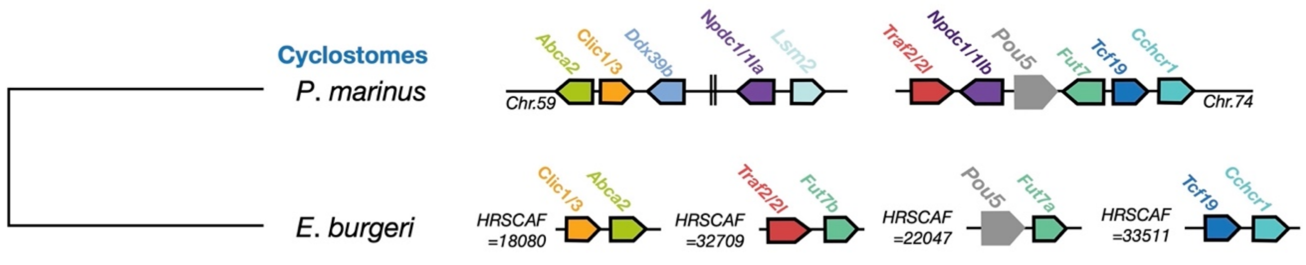

Supplementary Fig. 2, related to Fig. 1b. Detailed syntenies between *POU5* loci in vertebrates and related phylogenetic analyses. Conserved syntenies between gnathostome *POU5F1* (a), *POU5F3* (b) and cyclostome (c) POU5 loci. Genes identified in the genomes of the species analysed are depicted by coloured arrows. Black crosses over empty arrows indicate that the corresponding gene was not identified in the genome version analysed (see Supplementary Data 2). Continuous black lines connect genes found in synteny. The loci are identified by chromosome or scaffold names. Accession numbers are provided in Supplementary Data 2

a

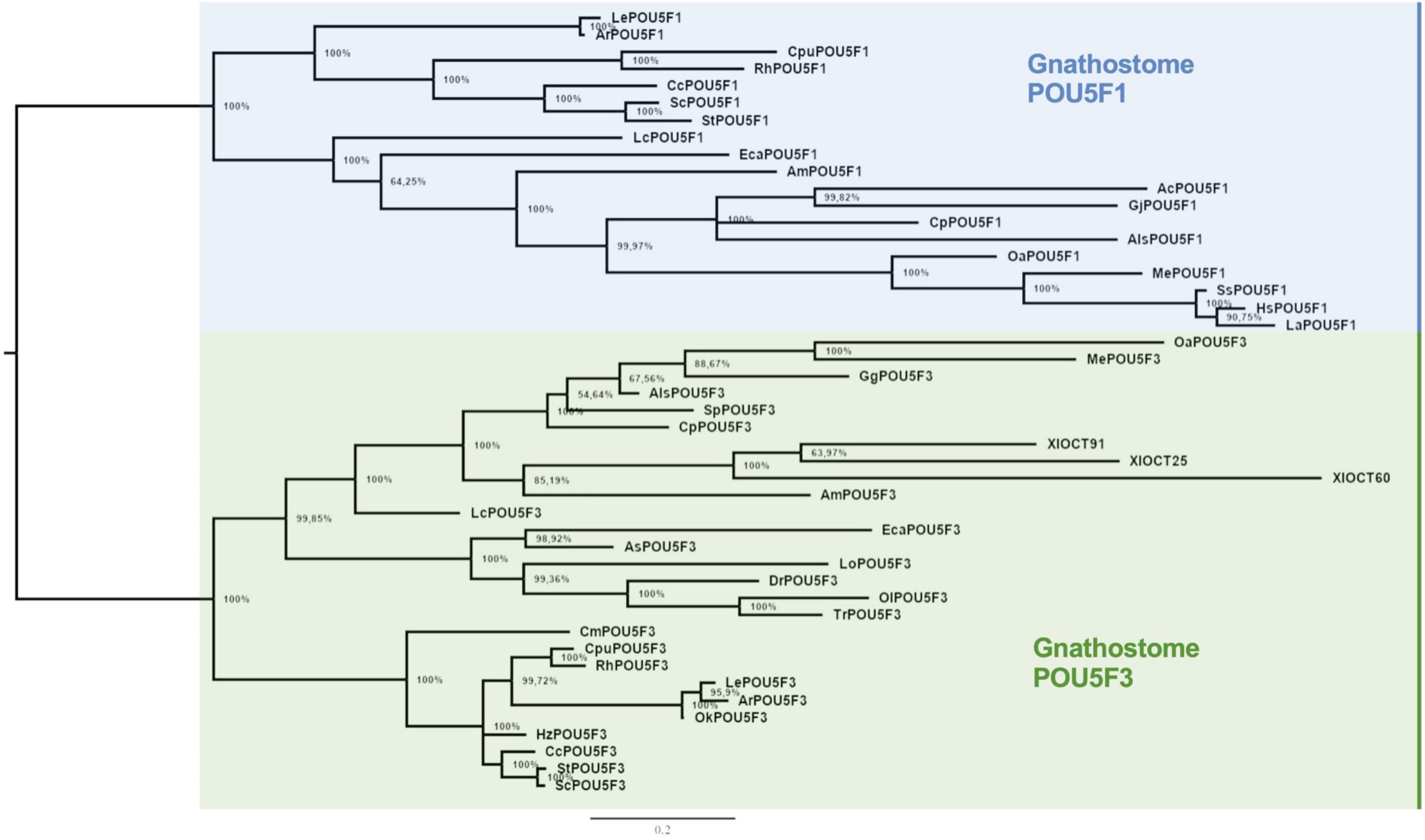
Gnathostome POU5F1
LePOU5F1
ArPOU5F1
CpuPOU5F1
RhPOU5F1
CcPOU5F1
ScPOU5F1
StPOU5F1
LcPOU5F1
EcaPOU5F1
AmPOU5F1
AcPOU5F1
GjPOU5F1
CpPOU5F1
AlsPOU5F1
OaPOU5F1
MePOU5F1
SsPOU5F1
HsPOU5F1
LaPOU5F1
OaPOU5F3
MePOU5F3
GgPOU5F3
AlsPOU5F3
SpPOU5F3
CpPOU5F3
XlOCT91
XlOCT25
XlOCT60
AmPOU5F3
LcPOU5F3
EcaPOU5F3
AsPOU5F3
LoPOU5F3
DrPOU5F3
OlPOU5F3
TrPOU5F3
CmPOU5F3
CpuPOU5F3
RhPOU5F3
LePOU5F3
ArPOU5F3
OkPOU5F3
HzPOU5F3
CcPOU5F3
StPOU5F3
ScPOU5F3
Gnathostome POU5F3
0.2

b

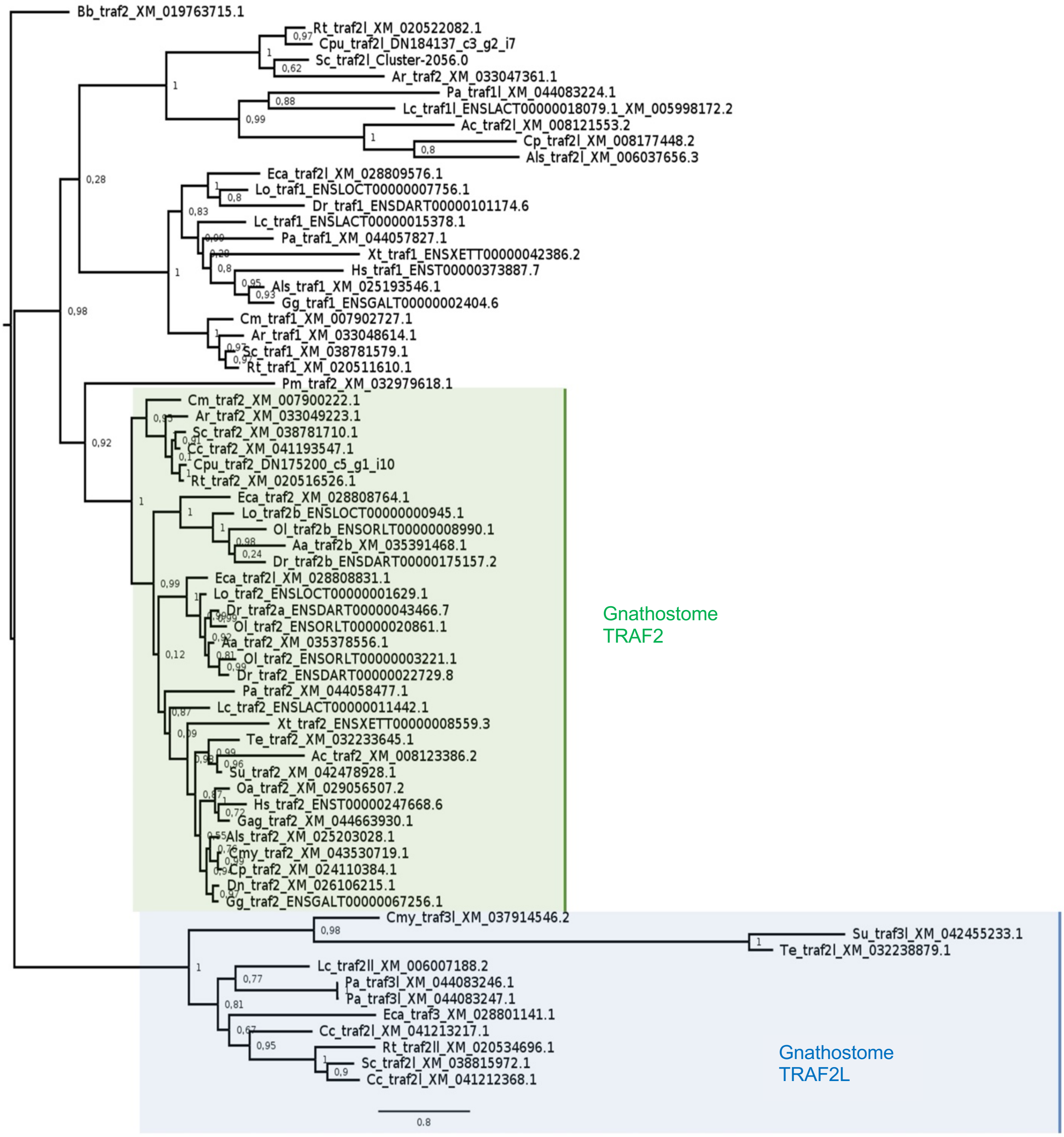
Bb_traf2_XM_019763715.1
Rt_traf2l_XM_020522082.1
Cpu_traf2l_DN184137_c3_g2_i7
Sc_traf2l_Cluster-2056.0
Ar_traf2_XM_033047361.1
Pa_traf1l_XM_044083224.1
Lc_traf1l_ENSLACT00000018079.1_XM_005998172.2
Ac_traf2l_XM_008121553.2
Cp_traf2l_XM_008177448.2
Als_traf2l_XM_006037656.3
Eca_traf2l_XM_028809576.1
Lo_traf1_ENSLOCT00000007756.1
Dr_traf1_ENSDART00000101174.6
Lc_traf1_ENSLACT00000015378.1
Pa_traf1_XM_044057827.1
Xt_traf1_ENSXETT00000042386.2
Hs_traf1_ENST00000373887.7
Als_traf1_XM_025193546.1
Gg_traf1_ENSGALT00000002404.6
Cm_traf1_XM_007902727.1
Ar_traf1_XM_033048614.1
Sc_traf1_XM_038781579.1
Rt_traf1_XM_020511610.1
Pm_traf2_XM_032979618.1
Cm_traf2_XM_007900222.1
Ar_traf2_XM_033049223.1
Sc_traf2_XM_038781710.1
Cc_traf2_XM_041193547.1
Cpu_traf2_DN175200_c5_g1_i10
Rt_traf2_XM_020516526.1
Eca_traf2_XM_028808764.1
Lo_traf2b_ENSLOCT00000000945.1
Ol_traf2b_ENSORLT00000008990.1
Aa_traf2b_XM_035391468.1
Dr_traf2b_ENSDART00000175157.2
Eca_traf2l_XM_028808831.1
Lo_traf2_ENSLOCT00000001629.1
Dr_traf2a_ENSDART00000043466.7
Ol_traf2_ENSORLT00000020861.1
Aa_traf2_XM_035378556.1
Ol_traf2_ENSORLT00000003221.1
Dr_traf2_ENSDART00000022729.8
Pa_traf2_XM_044058477.1
Lc_traf2_ENSLACT00000011442.1
Xt_traf2_ENSXETT00000008559.3
Te_traf2_XM_032233645.1
Ac_traf2_XM_008123386.2
Su_traf2_XM_042478928.1
Oa_traf2_XM_029056507.2
Hs_traf2_ENST00000247668.6
Gag_traf2_XM_044663930.1
Als_traf2_XM_025203028.1
Cmy_traf2_XM_043530719.1
Cp_traf2_XM_024110384.1
Dn_traf2_XM_026106215.1
Gg_traf2_ENSGALT00000067256.1
Cmy_traf3l_XM_037914546.2
Su_traf3l_XM_042455233.1
Te_traf2l_XM_032238879.1
Lc_traf2ll_XM_006007188.2
Pa_traf3l_XM_044083246.1
Pa_traf3l_XM_044083247.1
Eca_traf3_XM_028801141.1
Cc_traf2l_XM_041213217.1
Rt_traf2ll_XM_020534696.1
Sc_traf2l_XM_038815972.1
Cc_traf2l_XM_041212368.1
Gnathostome TRAF2
Gnathostome TRAF2L
0.8

c

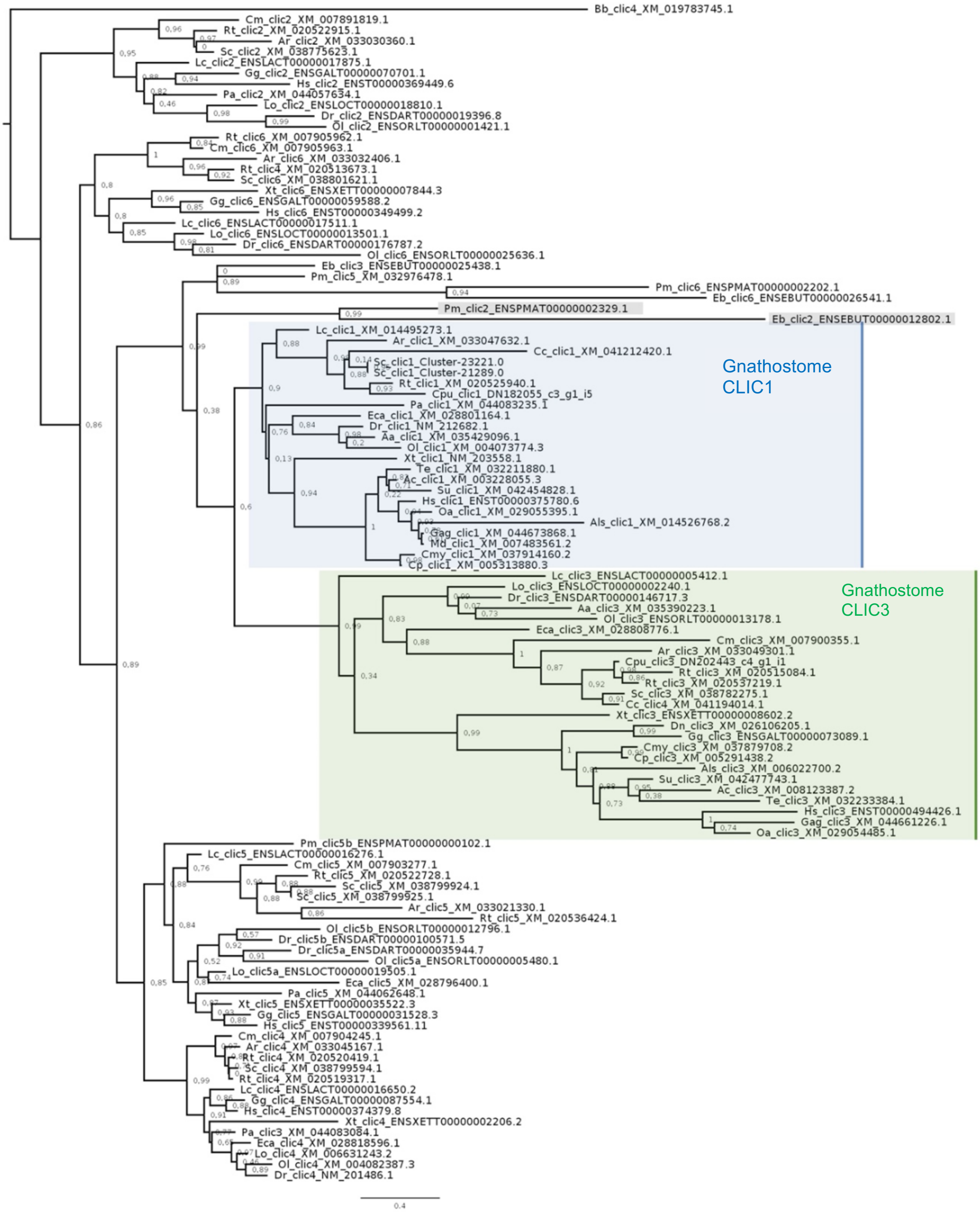

Bb_clic4_XM_019783745.1
Cm_clic2_XM_007891819.1
Rt_clic2_XM_020522915.1
Ar_clic2_XM_033030360.1
Sc_clic2_XM_038775623.1
Lc_clic2_ENSLACT00000017875.1
Gg_clic2_ENSGALT00000070701.1
Hs_clic2_ENST00000369449.6
Pa_clic2_XM_044057634.1
Lo_clic2_ENSLOCT00000018810.1
Dr_clic2_ENSDART00000019396.8
Ol_clic2_ENSORLT00000001421.1
Rt_clic6_XM_007905962.1
Cm_clic6_XM_007905963.1
Ar_clic6_XM_033032406.1
Rt_clic4_XM_020513673.1
Sc_clic6_XM_038801621.1
Xt_clic6_ENSXETT00000007844.3
Gg_clic6_ENSGALT00000059588.2
Hs_clic6_ENST00000349499.2
Lc_clic6_ENSLACT00000017511.1
Lo_clic6_ENSLOCT00000013501.1
Dr_clic6_ENSDART00000176787.2
Ol_clic6_ENSORLT00000025636.1
Eb_clic3_ENSEBUT00000025438.1
Pm_clic5_XM_032976478.1
Pm_clic6_ENSPMAT00000002202.1
Eb_clic6_ENSEBUT00000026541.1
Pm_clic2_ENSPMAT00000002329.1
Eb_clic2_ENSEBUT00000012802.1
Lc_clic1_XM_014495273.1
Ar_clic1_XM_033047632.1
Cc_clic1_XM_041212420.1
Sc_clic1_Cluster-23221.0
Sc_clic1_Cluster-21289.0
Rt_clic1_XM_020525940.1
Cpu_clic1_DN182055_c3_g1_i5
Pa_clic1_XM_044083235.1
Eca_clic1_XM_028801164.1
Dr_clic1_NM_212682.1
Aa_clic1_XM_035429096.1
Ol_clic1_XM_004073774.3
Xt_clic1_NM_203558.1
Te_clic1_XM_032211880.1
Ac_clic1_XM_003228055.3
Su_clic1_XM_042454828.1
Hs_clic1_ENST00000375780.6
Oa_clic1_XM_029055395.1
Als_clic1_XM_014526768.2
Gag_clic1_XM_044673868.1
Md_clic1_XM_007483561.2
Cmy_clic1_XM_037914160.2
Cp_clic1_XM_005313880.3
Gnathostome
CLIC1
Lc_clic3_ENSLACT00000005412.1
Lo_clic3_ENSLOCT00000002240.1
Dr_clic3_ENSDART00000146717.3
Aa_clic3_XM_035390223.1
Ol_clic3_ENSORLT00000013178.1
Eca_clic3_XM_028808776.1
Cm_clic3_XM_007900355.1
Ar_clic3_XM_033049301.1
Cpu_clic3_DN202443_c4_g1_i1
Rt_clic3_XM_020515084.1
Rt_clic3_XM_020537219.1
Sc_clic3_XM_038782275.1
Cc_clic4_XM_041194014.1
Xt_clic3_ENSXETT00000008602.2
Dn_clic3_XM_026106205.1
Gg_clic3_ENSGALT00000073089.1
Cmy_clic3_XM_037879708.2
Cp_clic3_XM_005291438.2
Als_clic3_XM_006022700.2
Su_clic3_XM_042477743.1
Ac_clic3_XM_008123387.2
Te_clic3_XM_032233384.1
Hs_clic3_ENST00000494426.1
Gag_clic3_XM_044661226.1
Oa_clic3_XM_029054485.1
Gnathostome
CLIC3
Pm_clic5b_ENSPMAT00000000102.1
Lc_clic5_ENSLACT00000016276.1
Cm_clic5_XM_007903277.1
Rt_clic5_XM_020522728.1
Sc_clic5_XM_038799924.1
Sc_clic5_XM_038799925.1
Ar_clic5_XM_033021330.1
Rt_clic5_XM_020536424.1
Ol_clic5b_ENSORLT00000012796.1
Dr_clic5b_ENSDART00000100571.5
Dr_clic5a_ENSDART00000035944.7
Ol_clic5a_ENSORLT00000005480.1
Lo_clic5a_ENSLOCT00000019505.1
Eca_clic5_XM_028796400.1
Pa_clic5_XM_044062648.1
Xt_clic5_ENSXETT00000035522.3
Gg_clic5_ENSGALT00000031528.3
Hs_clic5_ENST00000339561.11
Cm_clic4_XM_007904245.1
Ar_clic4_XM_033045167.1
Rt_clic4_XM_020520419.1
Sc_clic4_XM_038799594.1
Rt_clic4_XM_020519317.1
Lc_clic4_ENSLACT00000016650.2
Gg_clic4_ENSGALT00000087554.1
Hs_clic4_ENST00000374379.8
Xt_clic4_ENSXETT00000002206.2
Pa_clic3_XM_044083084.1
Eca_clic4_XM_028818596.1
Lo_clic4_XM_006631243.2
Ol_clic4_XM_004082387.3
Dr_clic4_NM_201486.1
0.4

d

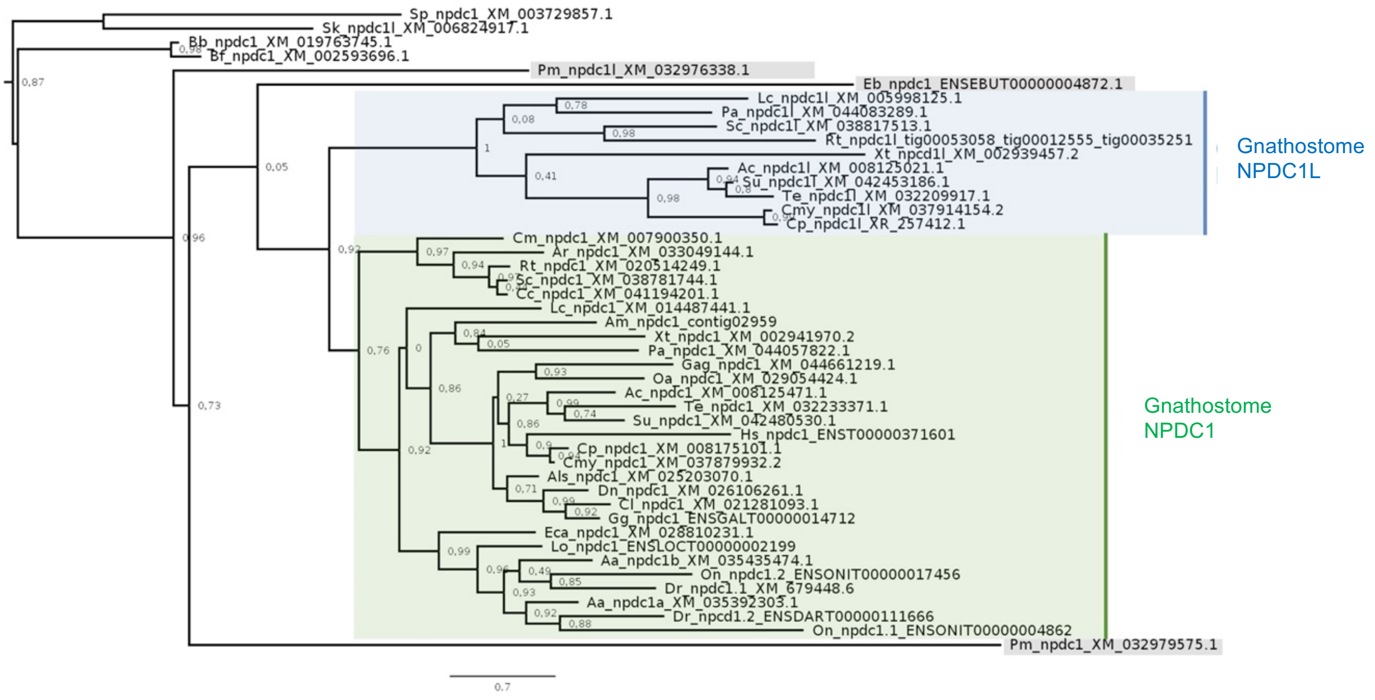
Sp_npdc1_XM_003729857.1
Sk_npdc1l_XM_006824917.1
Bb_npdc1_XM_019763745.1
Bf_npdc1_XM_002593696.1
Pm_npdc1l_XM_032976338.1
Eb_npdc1_ENSEBUT00000004872.1
Lc_npdc1l_XM_005998125.1
Pa_npdc1l_XM_044083289.1
Sc_npdc1l_XM_038817513.1
Rt_npdc1l_tig00053058_tig00012555_tig00035251
Xt_npcd1l_XM_002939457.2
Ac_npdc1l_XM_008125021.1
Su_npdc1l_XM_042453186.1
Te_npdc1l_XM_032209917.1
Cmy_npdc1l_XM_037914154.2
Cp_npdc1l_XR_257412.1
Gnathostome NPDC1L
Cm_npdc1_XM_007900350.1
Ar_npdc1_XM_033049144.1
Rt_npdc1_XM_020514249.1
Sc_npdc1_XM_038781744.1
Cc_npdc1_XM_041194201.1
Lc_npdc1_XM_014487441.1
Am_npdc1_contig02959
Xt_npdc1_XM_002941970.2
Pa_npdc1_XM_044057822.1
Gag_npdc1_XM_044661219.1
Oa_npdc1_XM_029054424.1
Ac_npdc1_XM_008125471.1
Te_npdc1_XM_032233371.1
Su_npdc1_XM_042480530.1
Hs_npdc1_ENST00000371601
Cp_npdc1_XM_008175101.1
Cmy_npdc1_XM_037879932.2
Als_npdc1_XM_025203070.1
Dn_npdc1_XM_026106261.1
Cl_npdc1_XM_021281093.1
Gg_npdc1_ENSGALT00000014712
Eca_npdc1_XM_028810231.1
Lo_npdc1_ENSLOCT00000002199
Aa_npdc1b_XM_035435474.1
On_npdc1.2_ENSONIT00000017456
Dr_npdc1.1_XM_679448.6
Aa_npdc1a_XM_035392303.1
Dr_npcd1.2_ENSDART00000111666
On_npdc1.1_ENSONIT00000004862
Gnathostome NPDC1
Pm_npdc1_XM_032979575.1
0.7

Supplementary Fig. 3. Phylogenetic trees showing the relationships between gnathostomes POU5F1 and POU5F3 proteins (a) and between paralogous genes used in synteny analyses: TRAF2/2L (b), CLIC1/3 (c), NPDC1/NPC1L (d). **(a)** is unrooted, **(b-d)** were rooted using amphioxus or hemichordate sequences. The trees were calculated using IQTree with the following parameters: NNI moves, LG+R4, aLRT branch support. The numbers at each node indicate posterior probabilities of group occurrence (in %). In (b-d), genes exhibiting conserved linkages with cyclostome *POU5* genes, gnathostome *POU5F1* and *POU5F3* are shaded in grey, blue and green respectively. Species abbreviations: Aa, *Anguilla anguilla*; As, *Acipenser sinensis*; Ac, *Anolis carolinensis*; Als, *Alligator sinensis*; Ar, *Amblyraja radiata*; Am, *Ambystoma mexicanum*; Amx, *Astyanax mexicanum*; Ao, *Amphiprion ocellaris*; Bb, *Branchiostoma belcheri*; Bf, *Branchiostoma floridae*; Cl, *Columbia livia*; Cm, *Callorhinchus milii*; Cp, *Chrysemys picta*; Cm, *Chelonia mydas*; Cpu, *Chiloscyllium punctatum*; Dn, *Dromaius novaehollandiae*; Dr, *Danio rerio*; Eb, *Eptatretus burgeri*; Gag, *Gracilinanus agilis*; Gg, *Gallus gallus*; Gm, *Gadus morhua*; Hs, *Homo sapiens*; Hz, *Heterodontus zebra*; La, *Loxodonta africana*; Lc, *Latimeria chalumnae*; Le, *Leucoraja erinacea*; Lj: *Lethenteron japonicum*; Lo, *Lepisosteus oculatus*; Md, *Monodelphis domestica*; Me, *Macropus eugenii*; Oa, *Ornithorhynchus anatinus*; Ok, *Okamejei kenojei*; Ol, *Oryzias latipes*; On, *Oreochromis niloticus*; Pa, *Protopterus annectens*; Pm, *Petromyzon marinus*; Ps, *Pelodiscus sinensis*; Rh/Rt, *Rhincodon typus*; Sc, *Scyliorhinus canicula*; Sk, *Saccoglossus kowalevskii*; Ss, *Sus scrofa*; Ssl, *Salmo salar*; St, *Scyliorhinus torazame*; Su, *Sceloporus undulatus*; Te, *Thamnophis elegans*; Tr, *Takifugu rubripes*; Xl, *Xenopus laevis*; Xt, *Xenopus tropicalis*.

a

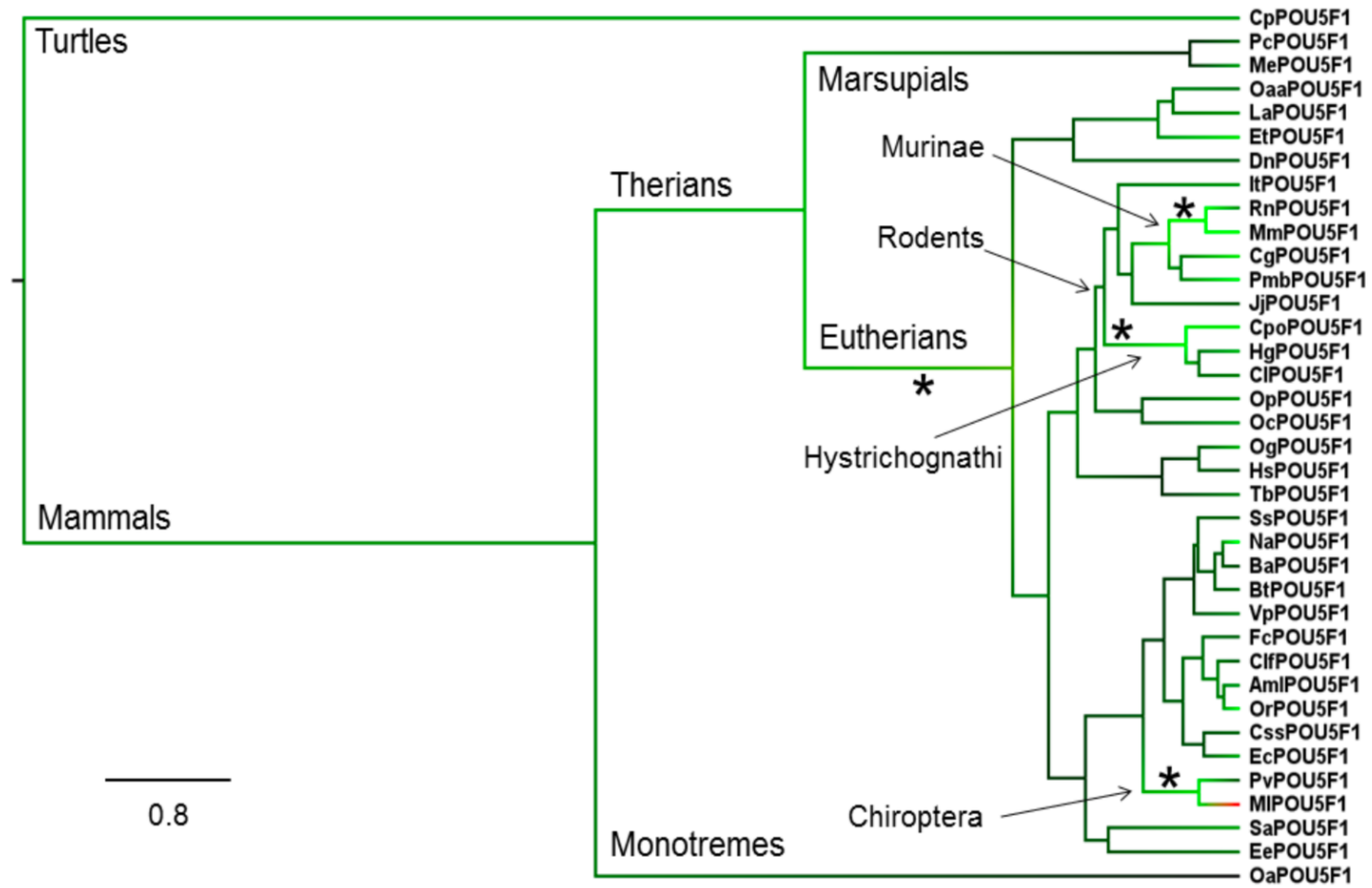

Turtles
Mammals
Therians
Marsupials
Eutherians
Monotremes
Murinae
Rodents
Hystrichognathi
Chiroptera
0.8
CpPOU5F1
PcPOU5F1
MePOU5F1
OaaPOU5F1
LaPOU5F1
EtPOU5F1
DnPOU5F1
ItPOU5F1
RnPOU5F1
MmPOU5F1
CgPOU5F1
PmbPOU5F1
JjPOU5F1
CpoPOU5F1
HgPOU5F1
ClPOU5F1
OpPOU5F1
OcPOU5F1
OgPOU5F1
HsPOU5F1
TbPOU5F1
SsPOU5F1
NaPOU5F1
BaPOU5F1
BtPOU5F1
VpPOU5F1
FcPOU5F1
ClfPOU5F1
AmlPOU5F1
OrPOU5F1
CssPOU5F1
EcPOU5F1
PvPOU5F1
MlPOU5F1
SaPOU5F1
EePOU5F1
OaPOU5F1

b

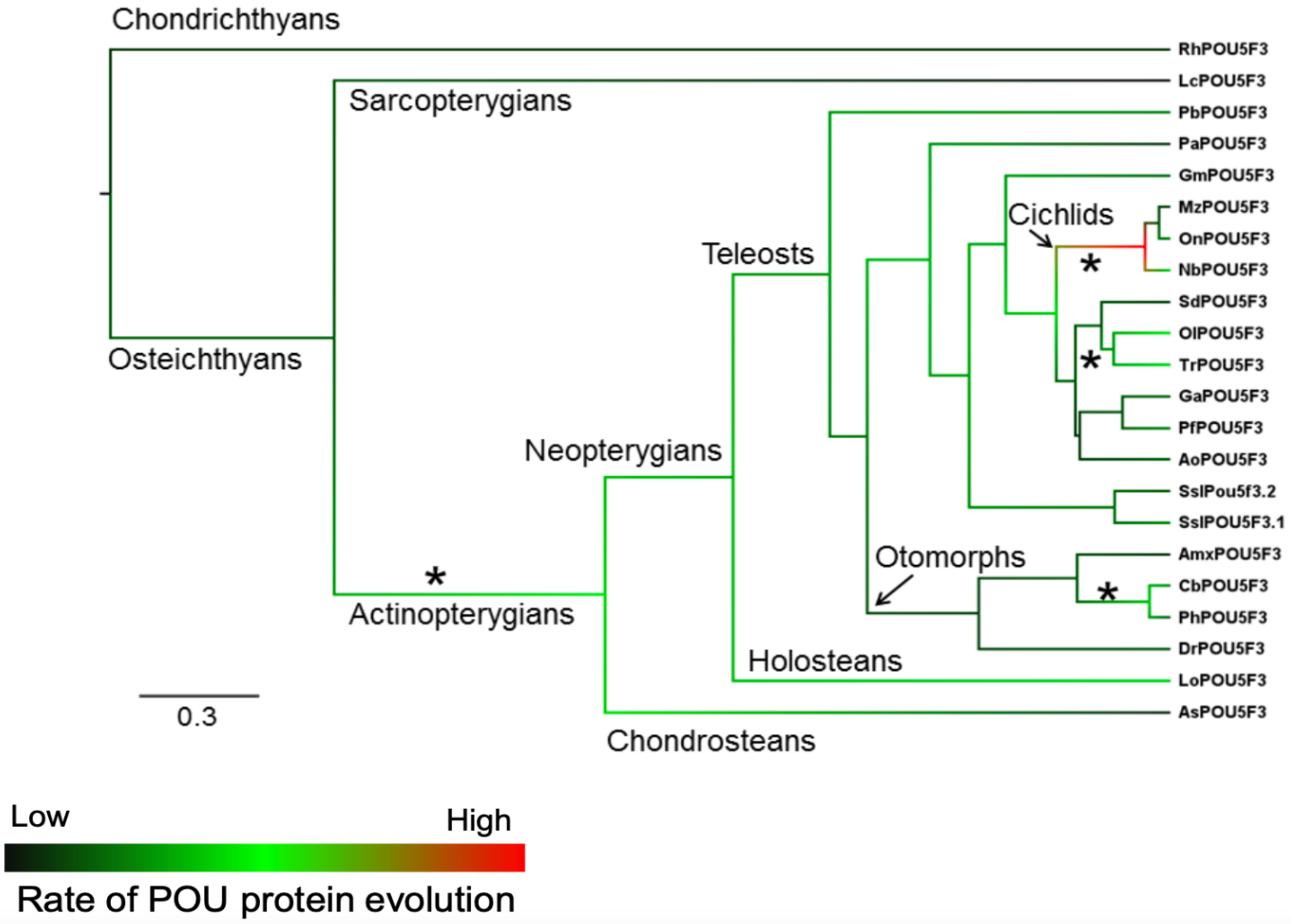
Chondrichthyans
Sarcopterygians
Osteichthyans
Actinopterygians
Neopterygians
Teleosts
Cichlids
Otomorphs
Holosteans
Chondrosteans
0.3
RhPOU5F3
LcPOU5F3
PbPOU5F3
PaPOU5F3
GmPOU5F3
MzPOU5F3
OnPOU5F3
NbPOU5F3
SdPOU5F3
OlPOU5F3
TrPOU5F3
GaPOU5F3
PfPOU5F3
AoPOU5F3
SslPou5f3.2
SslPOU5F3.1
AmxPOU5F3
CbPOU5F3
PhPOU5F3
DrPOU5F3
LoPOU5F3
AsPOU5F3
Low
High
Rate of POU protein evolution

Supplementary Fig. 4. Bayesian analyses of the evolution of mammalian POU5F1 proteins and actinopterygian POU5F3 proteins. Trees showing evolutionary rates of mammalian POU5F1 proteins (a) and actinopterygian POU5F3 proteins (b). Analyses were conducted on alignments of POU domain, linker, homeodomain and C-terminal sequences, imposing species phylogenies after ref.2 for mammals and ref.3 for teleosts. Evolutionary rates were calculated using the BEAST software and are represented in black, green to red from low, moderate to high. The turtle *C. picta* was used as outgroup for mammals. The whale shark *R. typus* and coelacanth *L. chalumnae* were used as outgroups for actinopterygians. Asterisks show branches in which accelerations of evolutionary rates have taken place. The scale bar corresponds to the average number of amino acid changes per site. Species name abbreviations: As, *Acipenser sinensis*; Aml, *Ailuropoda melanoleuca*; Amx, *Astyanax mexicanum*; Ao, *Amphiprion ocellaris*; Ba, *Balanoptera acutorostrata*; Bt: *Bos taurus*; Cb, *Clarias batrachus*; Cg, *Cricetulus griseus*; Clf, *Canis lupus familiaris*; Cl, *Chinchilla lanigera*; Cp, *Chrysemys picta*; Cpo, *Cavia porcellus*; Css, *Ceratotherium simum simum*; Dn, *Dasypus novemcinctus*; Dr, *Danio rerio*; Ec, *Equus caballus*; Ee, *Erinaceus europaeus*; Et, *Echinops telfairi*; Fc, *Felis catus*; Gm, *Gadus morhua*; Ga, *Gasterosteus aculeatus*; Hg, *Heterocephalus glaber*; Hs, *Homo sapiens*; It, *Ictidomys tridecemlineatus*; Jj, *Jaculus jaculus*; La, *Loxodonta africana*; Lc, *Latimeria chalumnae*; Lo, *Lepisosteus oculatus*; Me, *Macropus eugenii*; Ml, *Myotis lucifugus*; Mm, *Mus musculus*; Mz, *Maylandia zebra*; Na, *Neophocaena asiaorientalis*; Nb, *Neolamprologus brichardi*; Oa, *Ornithorhynchus anatinus*; Oaa, *Orycteropus afer afer*; Oc, *Oryctolagus cuniculus*; Og, *Otolemur garnettii*; Ol, *Oryzias latipes*; On, *Oreochromis niloticus*; Op, *Ochotona princeps*; Or, *Odobenus rosmarus divergens*; Pa, *Plecoglossus altivelis*; Pb, *Pantodon buchholzi*; Pc, *Phascolarctos cinereus*; Pf, *Perca fluviatilis*; Ph, *Pangasianodon hypophthalmus*; Pmb, *Peromyscus maniculatus bairdii*; Pv, *Pteropus vampyrus*; Rh/Rt, *Rhincodon typus*; Rn, *Rattus norvegicus*; Sa, *Sorex araneus*; Sd, *Seriola dumerili*; Ss, *Sus scrofa*; Ssl, *Salmo salar*; Tb, *Tupaia belangeri*; Tr, *Takifugu rubripes*; Vp, *Vicugna pacos*.

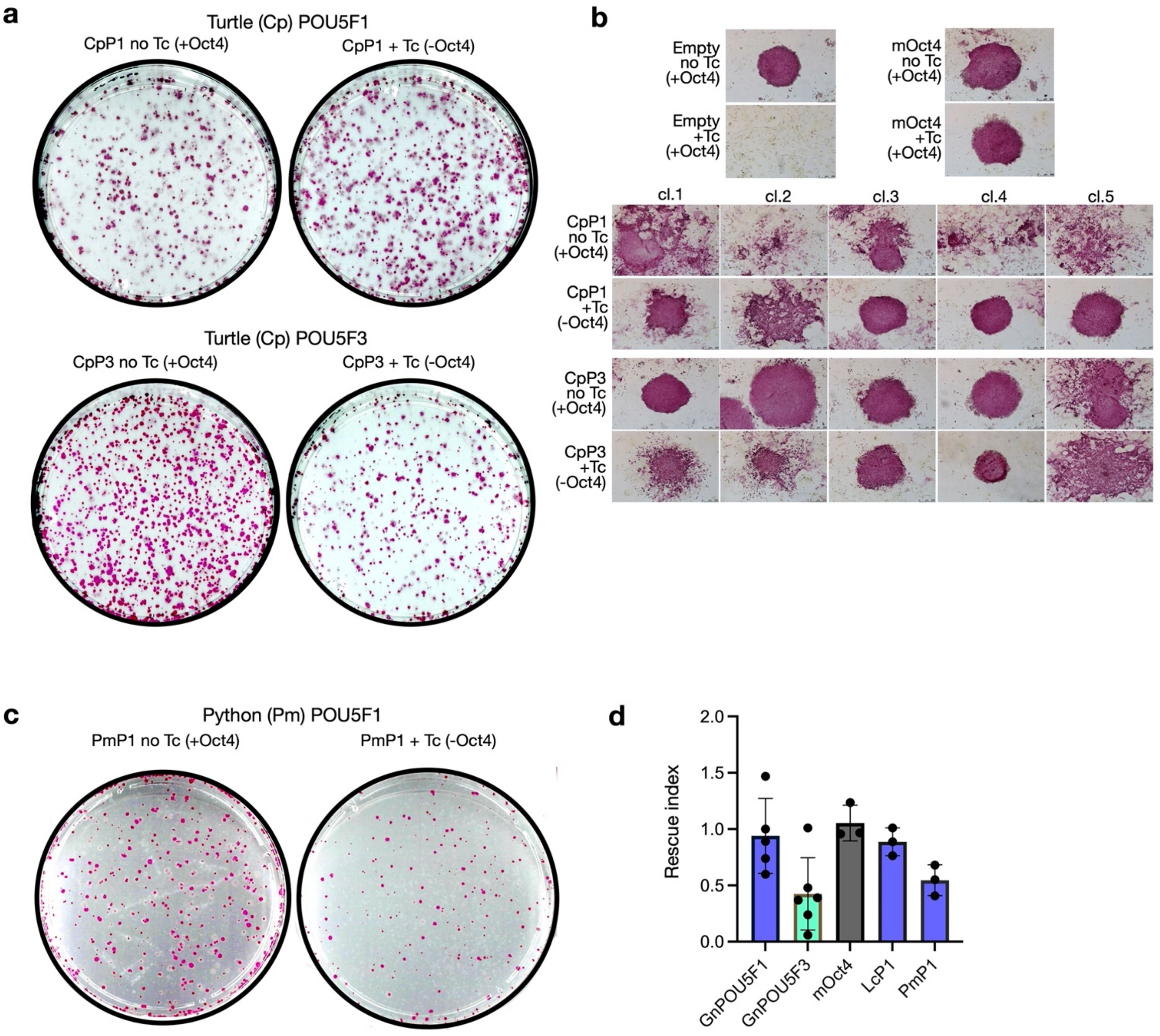
a
Turtle (Cp) POU5F1
CpP1 no Tc (+Oct4)
CpP1 + Tc (-Oct4)
Turtle (Cp) POU5F3
CpP3 no Tc (+Oct4)
CpP3 + Tc (-Oct4)
b
Empty no Tc (+Oct4)
Empty +Tc (+Oct4)
mOct4 no Tc (+Oct4)
mOct4 +Tc (+Oct4)
cl.1
cl.2
cl.3
cl.4
cl.5
CpP1 no Tc (+Oct4)
CpP1 +Tc (-Oct4)
CpP3 no Tc (+Oct4)
CpP3 +Tc (-Oct4)
c
Python (Pm) POU5F1
PmP1 no Tc (+Oct4)
PmP1 + Tc (-Oct4)
d
Rescue index
2.0
1.5
1.0
0.5
0.0
GnPOU5F1
GnPOU5F3
mOct4
LcP1
PmP1

Supplementary Fig. 5, related to Fig. 2. ESC colony morphology of reptilian POU5-rescued Oct4-null ESC cell lines.

a-c, Alkaline Phosphatase (AP) stainings. **a-b,** painted turtle POU5-rescued ESC colonies. **a**, Overview of AP stained colonies (grown in 10 cm dishes) of ZHBTc4 cells + Tetracycline (Tc) rescued with painted turtle (*Chrysemys picta*, Cp) POU5F1 and POU5F3. ZHBTc4 cells were electroporated with POU5 vectors and cultured in the absence or presence of tetracycline (= presence of Oct4 expression/absence of Oct4 expression). **b**, Colony morphologies of Cp POU5-rescued ESC colonies. **c**, Overview of AP stained colonies (grown in 10 cm dishes) of OCT4-null ESCs rescued with python (*Python molurus*, Pm) POU5F1 (PmP1), only POU5 form present in squamate. **d**, Rescue index of OCT4-null ESCs rescued with PmP1 compared to coelacanth and mouse POU5F1s (n=3) and average of gnathostome POU5F1s (GnPOU5F1, n=5) and POU5F3s (GnPOU5F3, n=6). Bar chart (d) show the mean of biologically independent samples +/- SD.

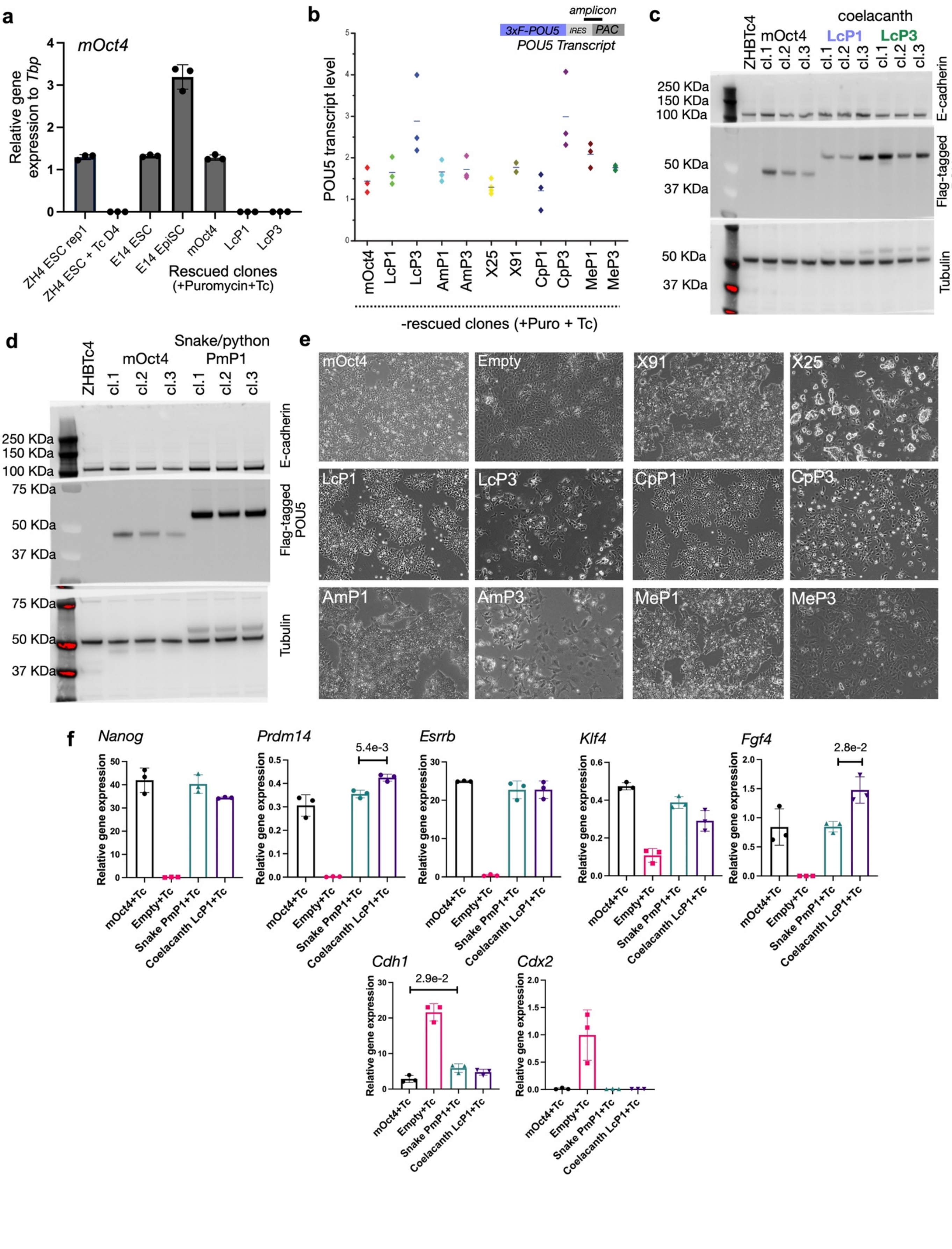
a
mOct4
Relative gene expression to Tbp
ZH4 ESC rep1
ZH4 ESC + Tc D4
E14 ESC
E14 EpiSC
mOct4
LcP1
LcP3
Rescued clones (+Puromycin+Tc)
b
amplicon
3xF-POU5
IRES
PAC
POU5 Transcript
POU5 transcript level
mOct4
LcP1
LcP3
AmP1
AmP3
X25
X91
CpP1
CpP3
MeP1
MeP3
-rescued clones (+Puro + Tc)
c
coelacanth
ZHBTc4
mOct4
LcP1
LcP3
cl.1
cl.2
cl.3
250 KDa
150 KDa
100 KDa
50 KDa
37 KDa
E-cadherin
Flag-tagged POU5
Tubulin
d
Snake/python
mOct4
PmP1
ZHBTc4
cl.1
cl.2
cl.3
250 KDa
150 KDa
100 KDa
75 KDa
50 KDa
37 KDa
E-cadherin
Flag-tagged POU5
Tubulin
e
mOct4
Empty
X91
X25
LcP1
LcP3
CpP1
CpP3
AmP1
AmP3
MeP1
MeP3
f
Nanog
Prdm14
Esrrb
Klf4
Fgf4
Cdh1
Cdx2
5.4e-3
2.8e-2
2.9e-2
Relative gene expression
mOct4+Tc
Empty+Tc
Snake PmP1+Tc
Coelacanth LcP1+Tc

Supplementary Fig. 6, related to Fig. 3. Additional phenotypes of POU5-rescued OCT4-null ESC cell lines.

a, Relative expression of mouse *Oct4* mRNA, measured by qRT-PCR, to confirm that POU5-rescued ESC clonal lines were maintained solely by transfected POU5 constructs. **b**, qRT-PCR showing expression of the puromycin-resistance gene used to indirectly measure POU5 transcript levels among different POU5-rescued clonal ESC lines. Data represented in the plot show average values of three independent clonal lines with the horizontal bars between diamond symbols representing the mean of these values. **c**, Western blot showing expression of flag-tagged POU5 proteins. **d**, Bright-field images of OCT4-null ESC clonal lines rescued by different POU5 proteins. Colonies from POU5-rescued ESCs were picked and expanded in ESC culture containing tetracycline (Tc) for 6 passages before further cell analysis using immunofluorescence and qRT-PCR (as shown in Fig. 3). OCT4-null cells don't grow in normal ESC medium because POU5 activity is required for colonies expansion, therefore no rescued colony could be picked from empty vector controls. To obtain ESCs without OCT4 expression, used as control for immunofluorescence and qRT-PCR in Fig. 3, ZHBTc4 cells were electroporated with the empty vector and cultured in the absence of Tc (presence of Oct4 expression). *Oct4* was later removed by addition of tetracycline for four days, to generate differentiated control cultures. **e**-**f**, additional phenotypes of python POU5F1-rescued cell lines. **e**, Western blot showing expression of flag-tagged PmP1 proteins. **f**, qRT-PCR, of OCT4-null ESCs rescued with PmP1, for pluripotency markers (*Nanog*, *Prdm14*, *Esrrb*, *Klf4* and *Fgf4*), cell adhesion (*Cdh1*) and differentiation markers (*Cdx2*). Abbreviations: Empty: empty vector; Puro, Puromycin; abbreviations for POU5 proteins are the same as in Fig. 2. Bar charts (a) show the mean of n=3 biologically independent samples +/- SD. Scatter plot (b) show each data points representing each biological clones (n=3) and line between data indicate mean. Bar charts (f) show the mean of n=3 biologically independent samples +/- SD with exact *p* values (95% confidence interval) comparing POU5F1 and POU5F3 determined by multiple Unpaired (two-tailed) *t*-tests, with Welch correction.

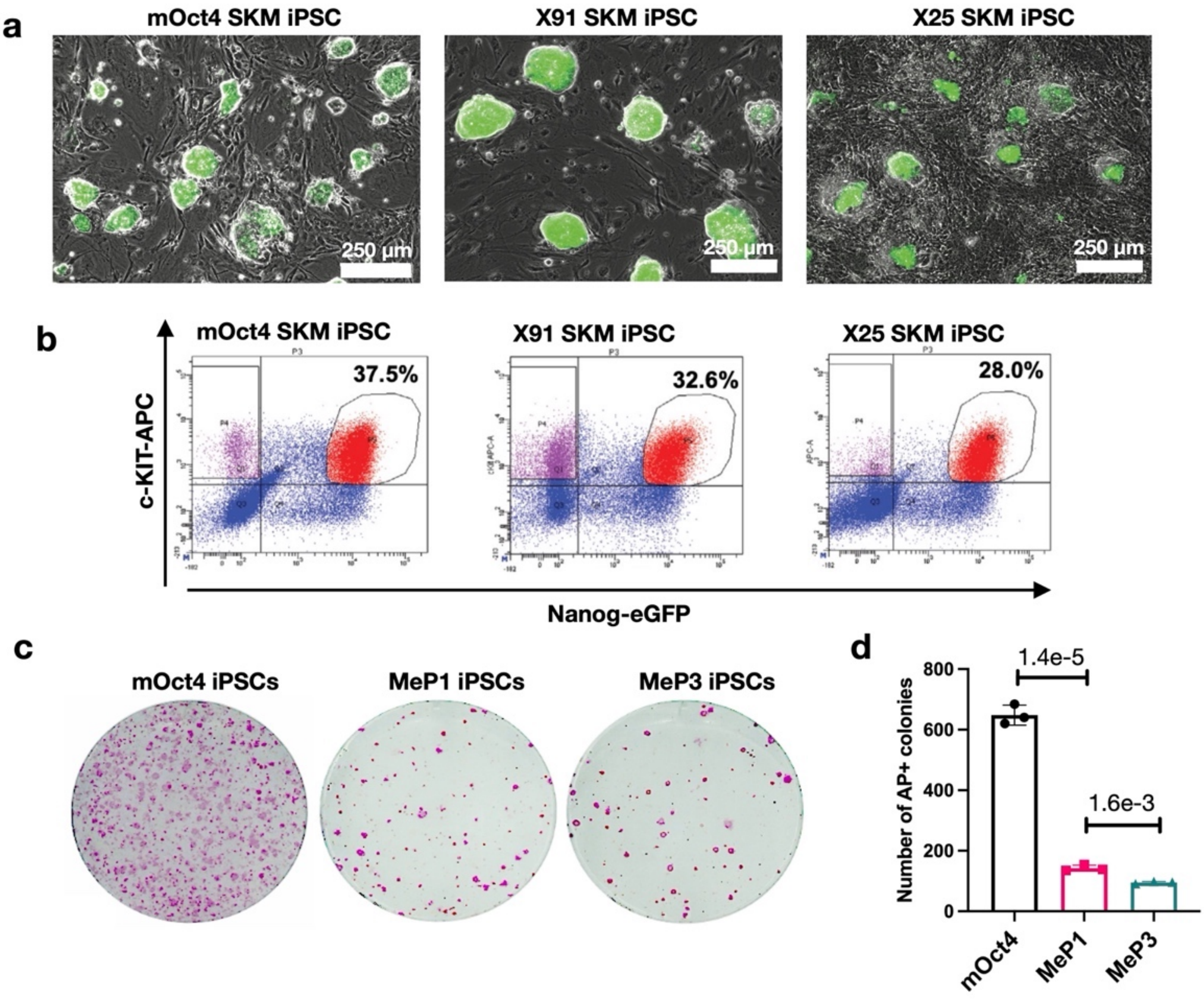
a
mOct4 SKM iPSC
X91 SKM iPSC
X25 SKM iPSC
250 µm
250 µm
250 µm
b
mOct4 SKM iPSC
X91 SKM iPSC
X25 SKM iPSC
37.5%
32.6%
28.0%
c-KIT-APC
Nanog-eGFP
c
mOct4 iPSCs
MeP1 iPSCs
MeP3 iPSCs
d
Number of AP+ colonies
800
600
400
200
0
1.4e-5
1.6e-3
mOct4
MeP1
MeP3

Supplementary Fig. 7, related to Fig. 4. Additional phenotypes of mouse iPSCs generated by different POU5s.

a, Merged brightfield and Nanog-eGFP (green) expression of iPSCs generated with different POU5 proteins: mOct4, X91 or X25 together with Sox2, Klf4 and c-Myc (SKM). **b,** Nanog-eGFP and a cell surface marker c-KIT profile for assessing naïve pluripotency of iPSCs generated from X91, X25 or mOct4 were analysed by flow cytometry and showed as dot plots. **c**, Overview of AP stainings (purple) of iPSCs generated by tammar wallaby Pou5f1 (MeP1) and Pou5f3 (MeP3) together with mSox2, mKlf4 and mc-Myc. **d**, Reprogramming efficiency of iPSCs generation comparing tammar wallaby POU5F1/3 (MeP1 and MeP3) to mOct4. Bar charts (d) show the mean of n=3 biologically independent samples +/- SD with exact *p* values (95% confidence interval) comparing iPSCs generated by different Oct4 homologues determined by multiple Unpaired (two-tailed) *t*-tests, with Welch correction.

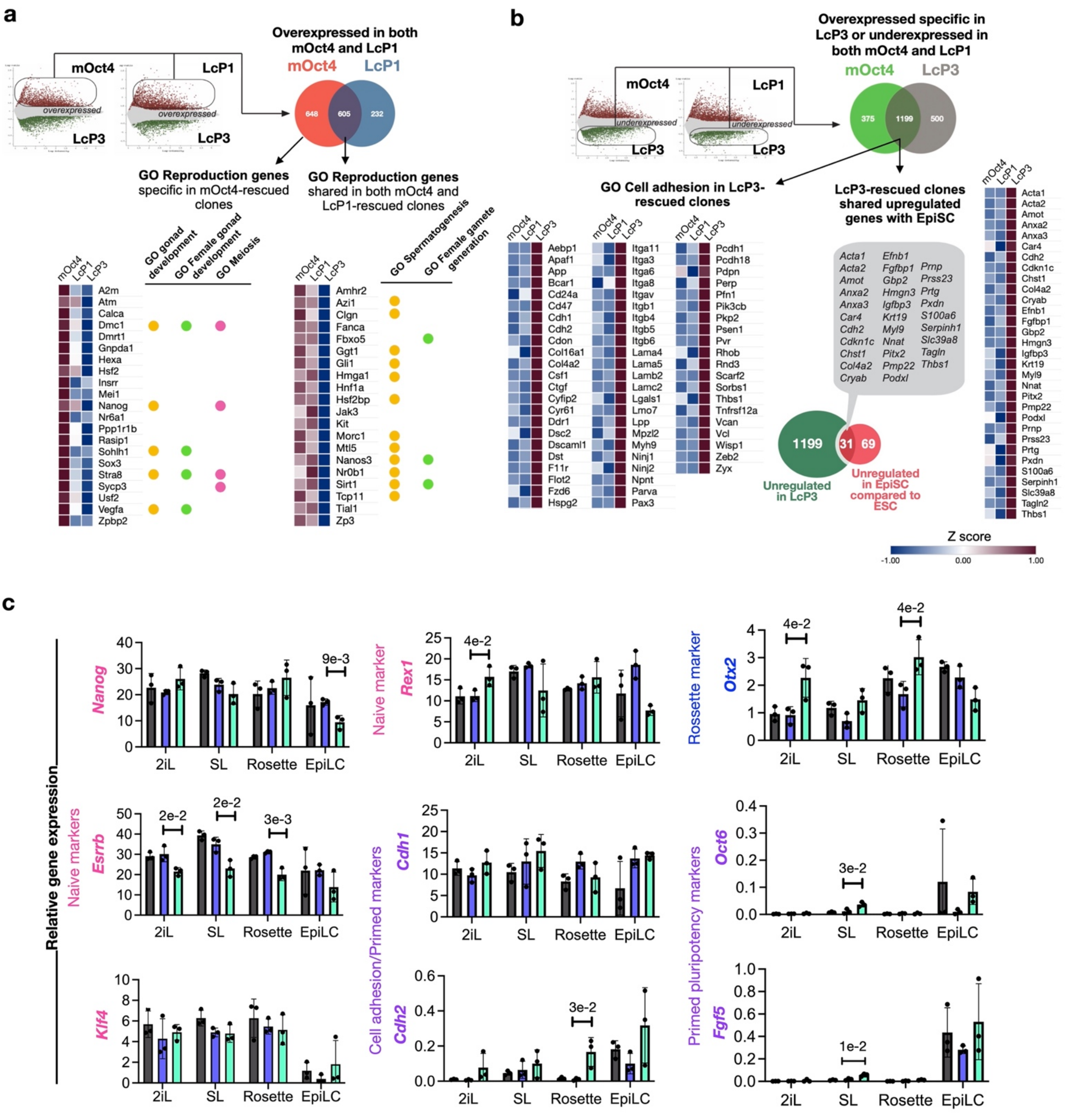

a
mOct4
LcP1
overexpressed
LcP3
Overexpressed in both mOct4 and LcP1
mOct4
LcP1
648
605
232
GO Reproduction genes specific in mOct4-rescued clones
GO Reproduction genes shared in both mOct4 and LcP1-rescued clones
GO gonad development
GO Female gonad development
GO Meiosis
A2m
Atm
Calca
Dmc1
Dmrt1
Gnpda1
Hexa
Hsf2
Insrr
Mei1
Nanog
Nr6a1
Ppp1r1b
Rasip1
Sohlh1
Sox3
Stra8
Sycp3
Usf2
Vegfa
Zpbp2
GO Spermatogenesis
GO Female gamete generation
Amhr2
Azi1
Clgn
Fanca
Fbxo5
Ggt1
Gli1
Hmga1
Hnf1a
Hsf2bp
Jak3
Kit
Morc1
Mtl5
Nanos3
Nr0b1
Sirt1
Tcp11
Tial1
Zp3
b
underexpressed
Overexpressed specific in LcP3 or underexpressed in both mOct4 and LcP1
375
1199
500
GO Cell adhesion in LcP3-rescued clones
LcP3-rescued clones shared upregulated genes with EpiSC
Aebp1
Apaf1
App
Bcar1
Cd24a
Cd47
Cdh1
Cdh2
Cdon
Col16a1
Col4a2
Csf1
Ctgf
Cyfip2
Cyr61
Ddr1
Dsc2
Dscaml1
Dst
F11r
Flot2
Fzd6
Hspg2
Itga11
Itga3
Itga6
Itga8
Itgav
Itgb1
Itgb4
Itgb5
Itgb6
Lama4
Lama5
Lamb2
Lamc2
Lgals1
Lmo7
Lpp
Mpzl2
Myh9
Ninj1
Ninj2
Npnt
Parva
Pax3
Pcdh1
Pcdh18
Pdpn
Perp
Pfn1
Pik3cb
Pkp2
Psen1
Pvr
Rhob
Rnd3
Scarf2
Sorbs1
Thbs1
Tnfrsf12a
Vcan
Vcl
Wisp1
Zeb2
Zyx
Acta1 Efnb1 Prnp
Acta2 Fgfbp1 Prss23
Amot Gbp2 Prtg
Anxa2 Hmgn3 Pxdn
Anxa3 Igfbp3 S100a6
Car4 Krt19 Serpinh1
Cdh2 Myl9 Slc39a8
Cdkn1c Nnat Tagln
Chst1 Pitx2 Thbs1
Col4a2 Pmp22
Cryab Podxl
1199
31
69
Unregulated in LcP3
Unregulated in EpiSC compared to ESC
Acta1
Acta2
Amot
Anxa2
Anxa3
Car4
Cdh2
Cdkn1c
Chst1
Col4a2
Cryab
Efnb1
Fgfbp1
Gbp2
Hmgn3
Igfbp3
Krt19
Myl9
Nnat
Pitx2
Pmp22
Podxl
Prnp
Prss23
Prtg
Pxdn
S100a6
Serpinh1
Slc39a8
Tagln2
Thbs1
Z score
-1.00
0.00
1.00
c
Relative gene expression
Naive markers
Nanog
9e-3
Esrrb
2e-2
2e-2
3e-3
Klf4
Naive marker
Rex1
4e-2
Cell adhesion/Primed markers
Cdh1
Cdh2
3e-2
Rossette marker
Otx2
4e-2
4e-2
Primed pluripotency markers
Oct6
3e-2
Fgf5
1e-2
2iL
SL
Rosette
EpiLC

Supplementary Fig. 8, related to Fig. 5. Difference in the gene expression profiles of OCT4-null ESCs supported by LcPOU5F1, mOct4 and LcPOU5F3.

a-b, Log-ratio plots showing significantly over-expressed (in red) and under-expressed (in green) probes, based on indicated pairwise comparisons (FDR ≤ 0.05). These gene lists were further filtered based on probes corresponding to uniquely annotated genes and with an absolute fold-change cut-off of 2. Differentially expressed genes from both comparisons were used to produce Venn diagrams to further identify common genes expressed in both mOct4 and LcP1-supported ESCs or specific to LcP3-supported ESCs. **a**, Over-expressed genes (605 genes) in common to mOct4 and LcP1-supported cells and over-expressed genes (648 genes) specific to mOct4-supported cells were analysed by the GO term analysis tool ShinyGO (FDR ≤ 0.05 and GO terms with more the 5 genes are shown). Expression of genes in the 'Reproduction' GO term were enriched in both mOct4- and LcP1-rescued clones were shown as heatmaps. **b**, Over-expressed genes (1199) in LcP3-supported cells, but down-regulated in both mOct4- and LcP1-suppoted cell lines were analysed by GO term analysis (Left panel). Heatmaps of genes from the 'biological adhesion' GO term were enriched in the LcP3-rescued clones (Right panel). The set of genes specifically up-regulated in LcP3-supported ESCs (1199 genes) were compared to top 100 most significant genes up-regulated in EpiSC when compared to naïve ESCs. List of genes shared in both EpiSC and LcPOU5F3-rescued clones were used to construct a heatmap to visualise their expression. **c**, Relative gene expression of naïve, rosette and primed pluripotency markers and cell adhesion markers of mOct4, LcP1 or LcP3-rescued cells under SL/Rosette/EpiLC culture conditions were analysed by qRT-PCR, as shown in Fig. 5e. Bar charts (c) show the mean of n=3 biologically independent samples +/- SD with exact *p* values (95% confidence interval) comparing POU5F1 and POU5F3-rescued clones in each culture condition determined by multiple Unpaired (two-tailed) *t*-tests, with Welch correction.

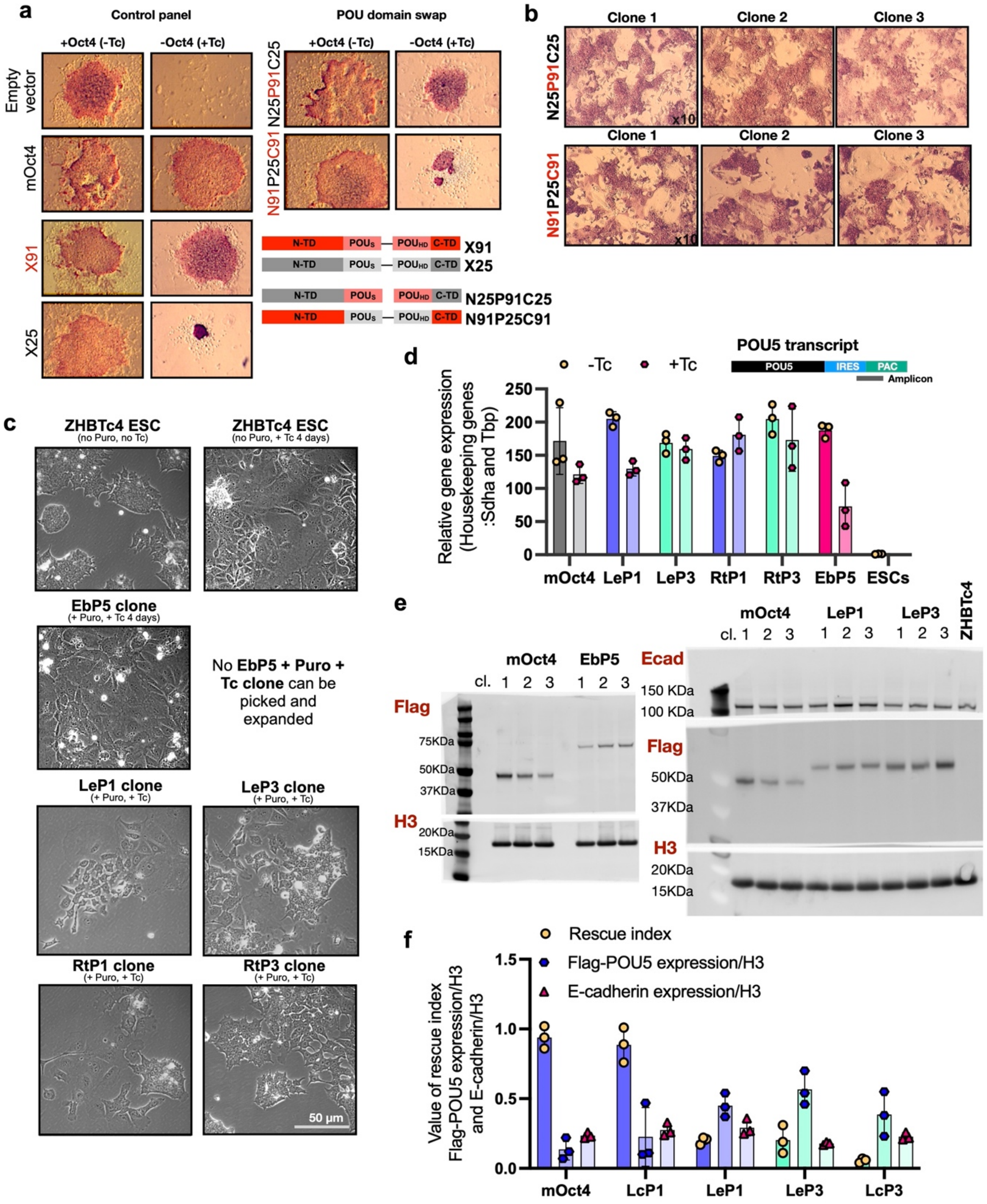
a
Control panel
POU domain swap
+Oct4 (-Tc)
-Oct4 (+Tc)
Empty vector
mOct4
X91
X25
N25P91C25
N91P25C91
N-TD
POUs
POUHD
C-TD
X91
X25
N25P91C25
N91P25C91
b
Clone 1
Clone 2
Clone 3
N25P91C25
N91P25C91
x10
c
ZHBTc4 ESC
(no Puro, no Tc)
ZHBTc4 ESC
(no Puro, + Tc 4 days)
EbP5 clone
(+ Puro, + Tc 4 days)
No EbP5 + Puro + Tc clone can be picked and expanded
LeP1 clone
(+ Puro, + Tc)
LeP3 clone
(+ Puro, + Tc)
RtP1 clone
(+ Puro, + Tc)
RtP3 clone
(+ Puro, + Tc)
50 μm
d
-Tc
+Tc
POU5 transcript
POU5
IRES
PAC
Amplicon
Relative gene expression (Housekeeping genes :Sdha and Tbp)
250
200
150
100
50
0
mOct4
LeP1
LeP3
RtP1
RtP3
EbP5
ESCs
e
mOct4
EbP5
cl. 1 2 3
Flag
75KDa
50KDa
37KDa
H3
20KDa
15KDa
mOct4
LeP1
LeP3
ZHBTc4
Ecad
150 KDa
100 KDa
Flag
50KDa
37KDa
H3
20KDa
15KDa
f
Rescue index
Flag-POU5 expression/H3
E-cadherin expression/H3
Value of rescue index Flag-POU5 expression/H3 and E-cadherin/H3
1.5
1.0
0.5
0.0
mOct4
LcP1
LeP1
LeP3
LcP3

Supplementary Fig. 9, related to Fig. 6. Chondrichthyans POU5 rescue activity and functional domains.

Mapping the functional domains of POU5 proteins responsible for rescue of OCT4-null ESCs.

As *Xenopus* proteins have a similar coding sequence, but differ dramatically in their rescue activity, we used these proteins to initially map the functional domains responsible for rescue. **a**, Morphology of chimeric POU5-rescued ESC colonies stained for alkaline phosphatase (AP, purple) activity. OCT4-null ESCs were rescued by X91-X25 chimeric proteins and data for the POU domain swap is shown. X91 is as effective as mOct4 at supporting ESC self-renewal. Swapping POU domain of X91 into X25 improved rescue activity of X25 while replacement of POU domain in X91 from X25 diminished the capacity of X91 to support ESCs. **b**, Stable OCT4-null ESCs cultures rescued with X91-X25 chimeric proteins were also stained for AP activity.

Chondrichthyan, but not cyclostome POU5 proteins rescue Oct4 phenotypes.

c-f, Analysis of expanded, stable OCT4-null ESC clonal lines rescued by Chondrichthyan POU5 proteins. As Hagfish POU5 (EbP5) cannot rescue, EbP5 expressing colonies were picked and expanded in the absence of Tc, then treated with Tc for four days to remove OCT4 before imaging. **c**, Bright-field images of expanded colonies. **d**, Expression of the puromycin-resistant gene used to indirectly measure POU5 transcript levels between different POU5-rescued clonal lines, measured by qRT-PCR. **e**, Western blots showing levels of FLAG tagged proteins, representing levels of POU5 and E-cadherin (Ecad), in three clones per rescued line with H3 as a loading control. **f**, Bar chart showing the comparison between the rescue index for each species and the relative POU5 and E-cadherin protein levels from stable rescued lines (see Supplementary Fig. 6). Individual data points in the bar graphs represent values from three independent clonal lines and bar charts (d and f) show mean of n=3 biologically independent samples +/- SD

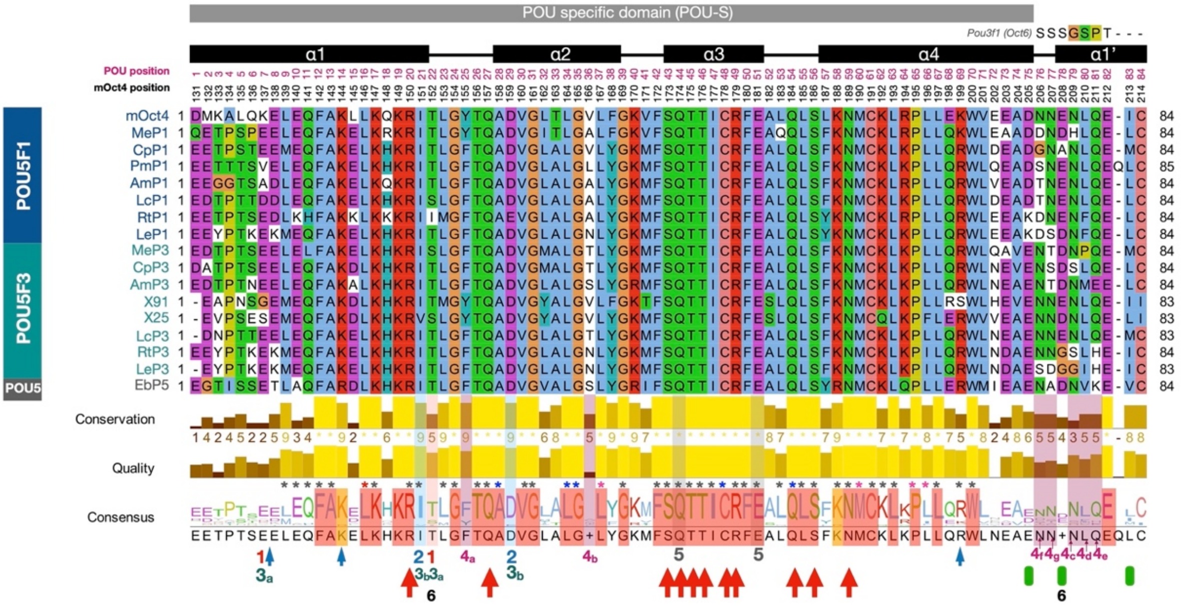

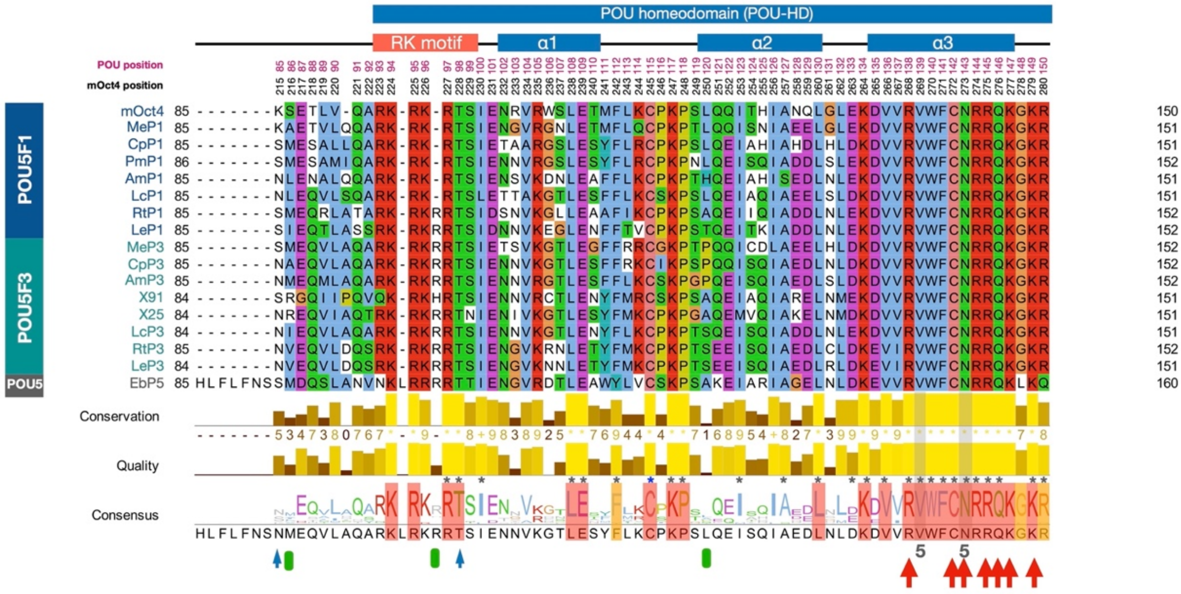

Abbr.	Details	Ref.
Red box	Conserved residues in all POU5s from both cyclostome and gnathostomes	In this study
Orange box	Conserved residues in all POU5s from gnathostomes but not in cyclostomes	In this study
*	(Grey) Conserved residues found in Pit1, Oct1, Oct2, Oct6 and Oct4<br>(Red) Conserved residues found only in all Oct4 homologues, not in Pit1, Oct1, Oct2, Oct6<br>(Blue) Conserved residues found only in all Oct4 homologues and Oct6, not in Pit1, Oct1, Oct2<br>(Pink) Conserved residues found in Oct1, Oct2, Oct6 and Oct4 but not in Pit1	Jin et al., 2016
Red arrow	Residues interacting with DNA based on AlphaFold2-structural models in this study	In this study
Blue arrow	**Protein modifications (based on mouse Oct4)**<br>**Abbr.** Ub, Ubiquitylation; P, Phosphorylation<br>**K137**$^{(POU7)}$: Ub; **K144**$^{(POU14)}$: Ub; **K199**$^{(POU69)}$: Ub; **K215**$^{(POU85)}$: Ub; **T228**$^{(POU98)}$: P	PhospoSite Plus (v6.6.0.2)
	Potential POU5F1 specific residues	In this study

Abbr.	Details	Ref.
1	Residues required for maintenance of pluripotency	Nishimoto et al., 2005
2	Residues required for Oct4-Sox2 interaction on canonical *SoxOct* elements	Remenyi et al., 2003; Merino et al., 2014
3	-**3a** - Mutation at these residues in mOct4 can still generate iPSCs (60% to WT)<br>-**3b** - Mutation at these residues in mOct4 can no longer generate iPSCs & Importance for Sox2-Oct4 heterodimerization.	Jerabek et al., 2017
4	**Oct4 structure**<br>-**4a** (Y155$^{(POU25)}$ in mOct4) interacting with **4e** (Q211$^{(POU81)}$ in linker region of mOct4)<br>-**4b** (V166$^{(POU36)}$ in mOct4) interface with C-terminal part of the linker α 1'.<br>-**4c** and **4d** (N209$^{(POU79)}$ and L210$^{(POU80)}$ in linker) exposed to surface of linker.<br>**Oct4 in iPSC generation and ESCs**<br>-Mutation in **4b**, **4f**, **4g**, **4c** (V166K$^{(POU36)}$, N206A$^{(POU76)}$, N207A$^{(POU77)}$, N209A$^{(POU79)}$ in linker) - reduce iPSC colonies<br>-Mutation in **4d**, **4e** (L210A$^{(POU80)}$, Q211R$^{(POU81)}$ in linker) - no iPSCs<br>-*Xenopus* Xlpou91 with residue "NLQ" in linker (**4c**,**4d**,**4e**)— produce iPSC colonies similar to mOct4.<br>-Zebrafish POU5F3 with residue "P" in linker (**4d**) - no iPSCs<br>-Replacing Oct4 linker with Oct6 linker - no iPSCs<br>**In ZHBTc4 model**<br>-Quadruple mutant (N206A$^{(POU76)}$, N207A$^{(POU77)}$, N209A$^{(POU79)}$, L210A$^{(POU80)}$ - **4f, 4g, 4c, 4d**) - no ESC rescue<br>-Mutation in **4d** (L210A$^{(POU80)}$) - partial ESC rescue* ; impaired embryonic development**	Esch et al., 2013<br>*also in Dong et al., 2022<br>**New finding in Dong et al., 2022
5	Mutation in these residue - no iPSCs	Jin et al., 2016
6	Mutation in these residues (T152R$^{(POU22)}$, E208P$^{(POU78)}$) potentially re-shaping the linker helix, enhancing cellular reprogramming	Tan et al., 2021

Supplementary Fig. 10, related to Fig. 7. Multiple sequence alignment of POU5 proteins used in Oct4 rescue assays.

Protein sequences from POU5s were aligned using MUSCLE and visualized in Jalview[4]. Consensus logo indicating conservation level is shown below the alignment. The uniqueness of each residue among POU5 proteins was identified based on literature review and AlphaFold2-based protein-DNA interaction in this study. Residue conservation among POU proteins (PIT1, OCT1, OCT2, OCT4 and OCT6) is marked with coloured asterisks on the top of consensus logo and level of conservation is shown with coloured boxes over the consensus sequence, explanation below the alignment (lower left). POU domain-DNA interactions from AlphaFold2-based mOct4 structural models using ChimeraX are noted with red arrows and residues with reported post-translational modifications are noted with blue arrows. Literature references for functional analysis of residues are noted under the consensus logo as a table (1-6[5-14]), with the role of the residue detailed below the alignment (lower right).

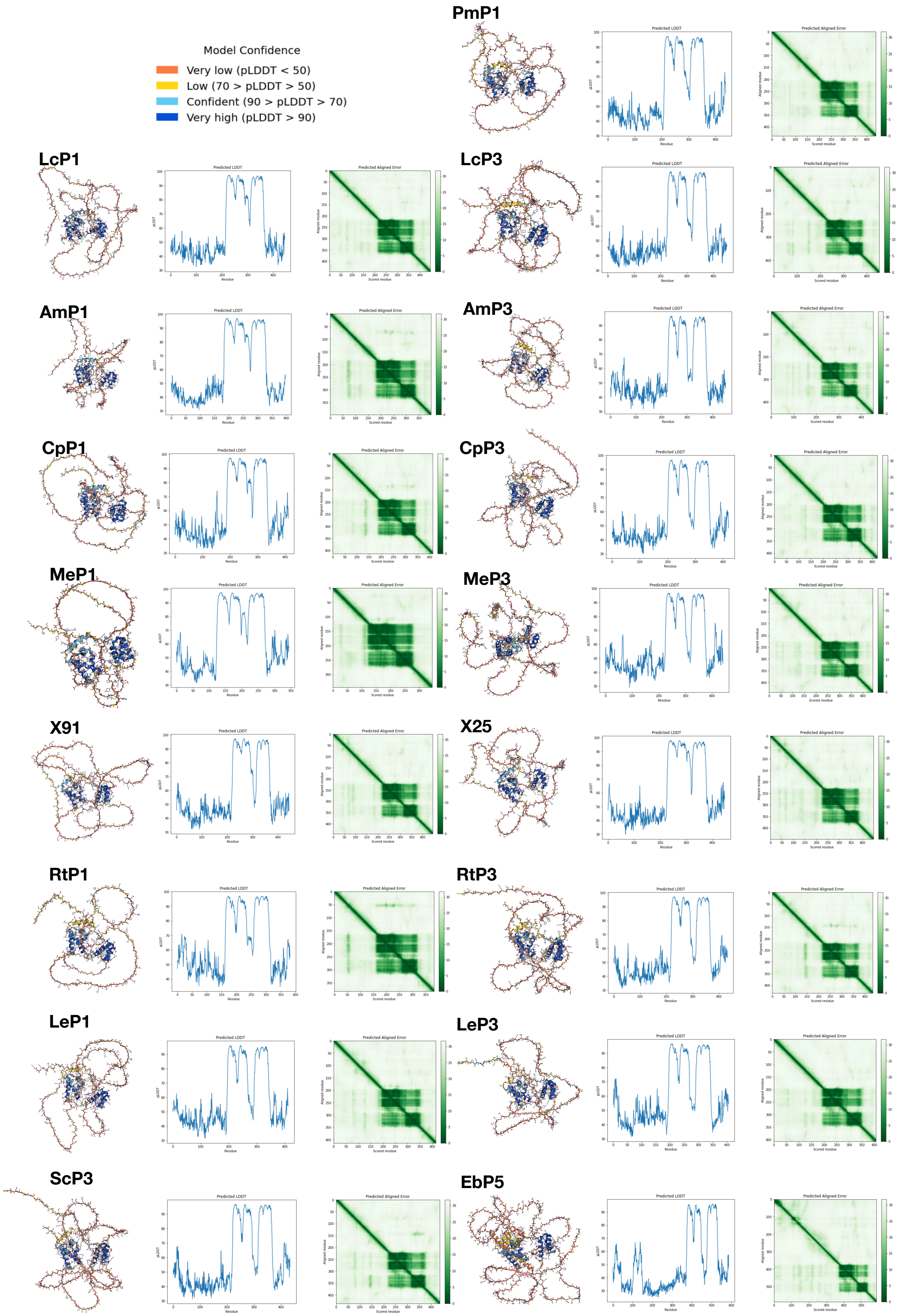

Supplementary Fig. 11. Outputs from AlphaFold2-structural modelling of POU5 proteins. Including 3D coordinates (left), per-residue confidence metric called pLDDT (middle) and Predicted Aligned Error (right)

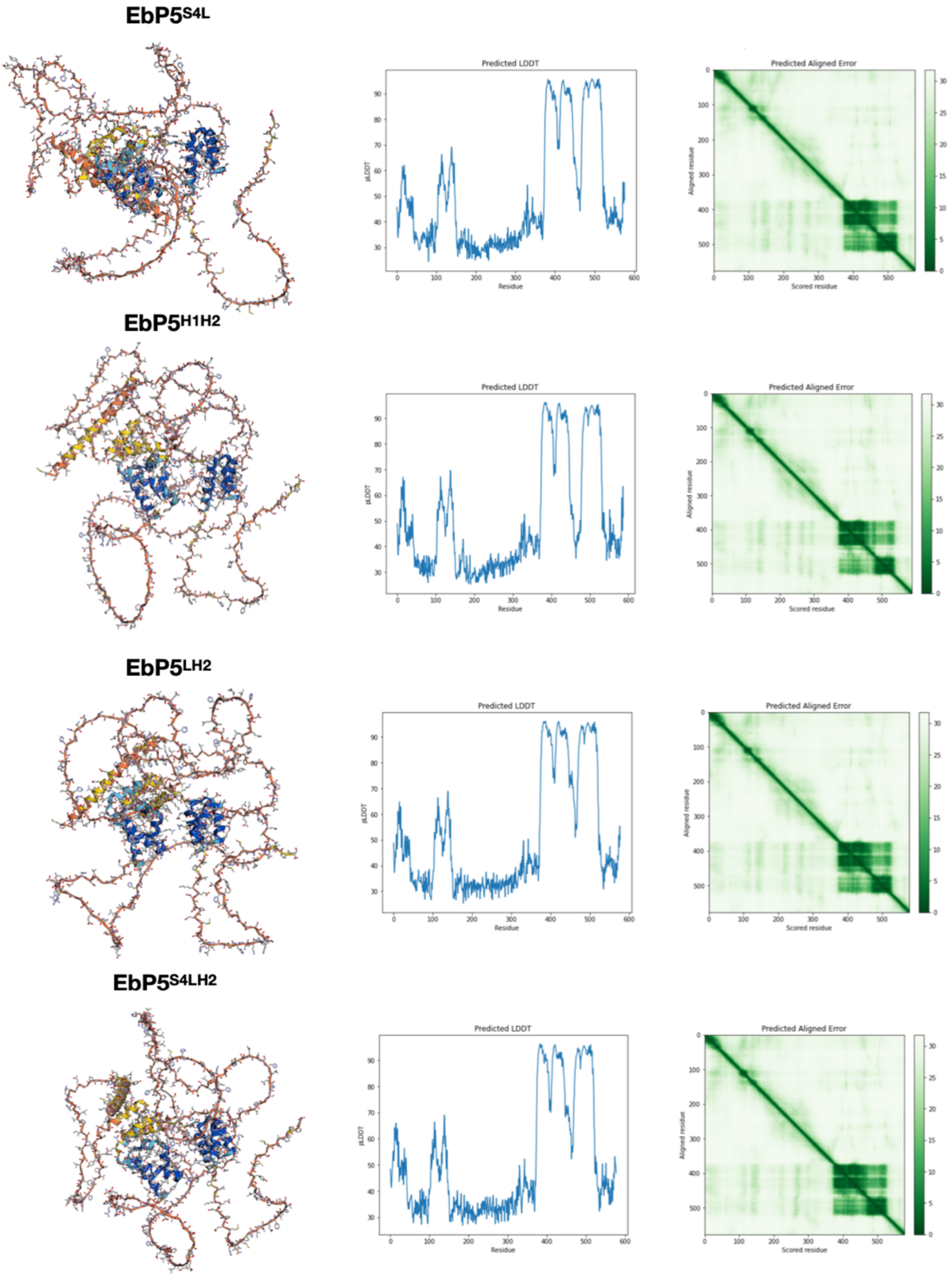

Supplementary Fig. 12. Outputs from AlphaFold2-structural modelling of hagfish-coelacanth chimeric POU5 proteins. Including 3D coordinates (left), per-residue confidence metric called pLDDT (middle) and Predicted Aligned Error (right)

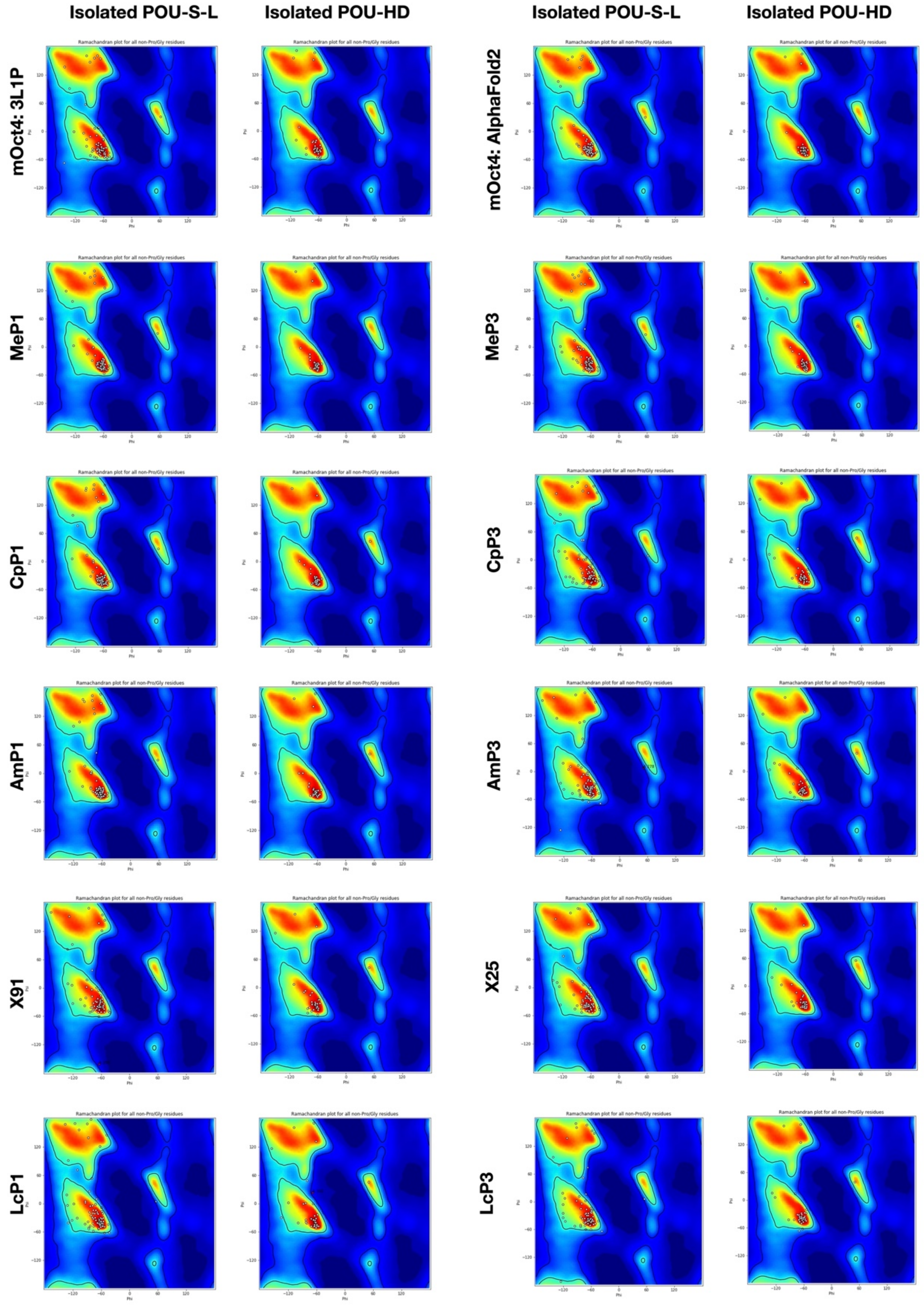

Supplementary Fig. 13. Ramachandran plots of POU5 structural models. Isolated POU specific domain including structural linker (POU-S-L) and isolated POU homeodomain (POU-HD) from each Alphafold2-predicted POU5 protein structures were analysed by Phenix.

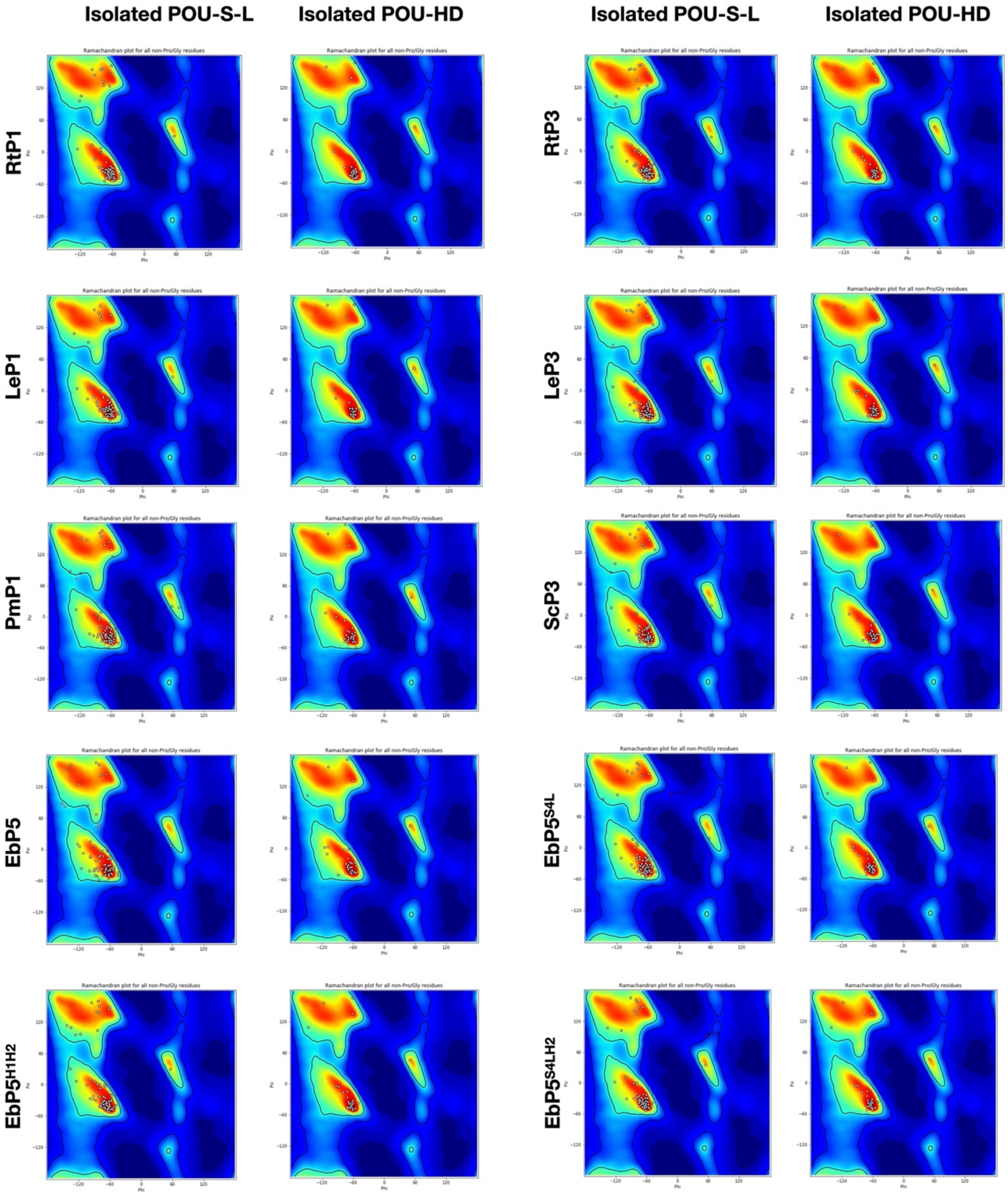

Supplementary Fig. 13 (Continued). Ramachandran plots of POU5 structural models. Isolated POU specific domain including structural linker (POU-S-L) and isolated POU homeodomain (POU-HD) from each Alphafold2-predicted POU5 protein structures were analysed by Phenix.

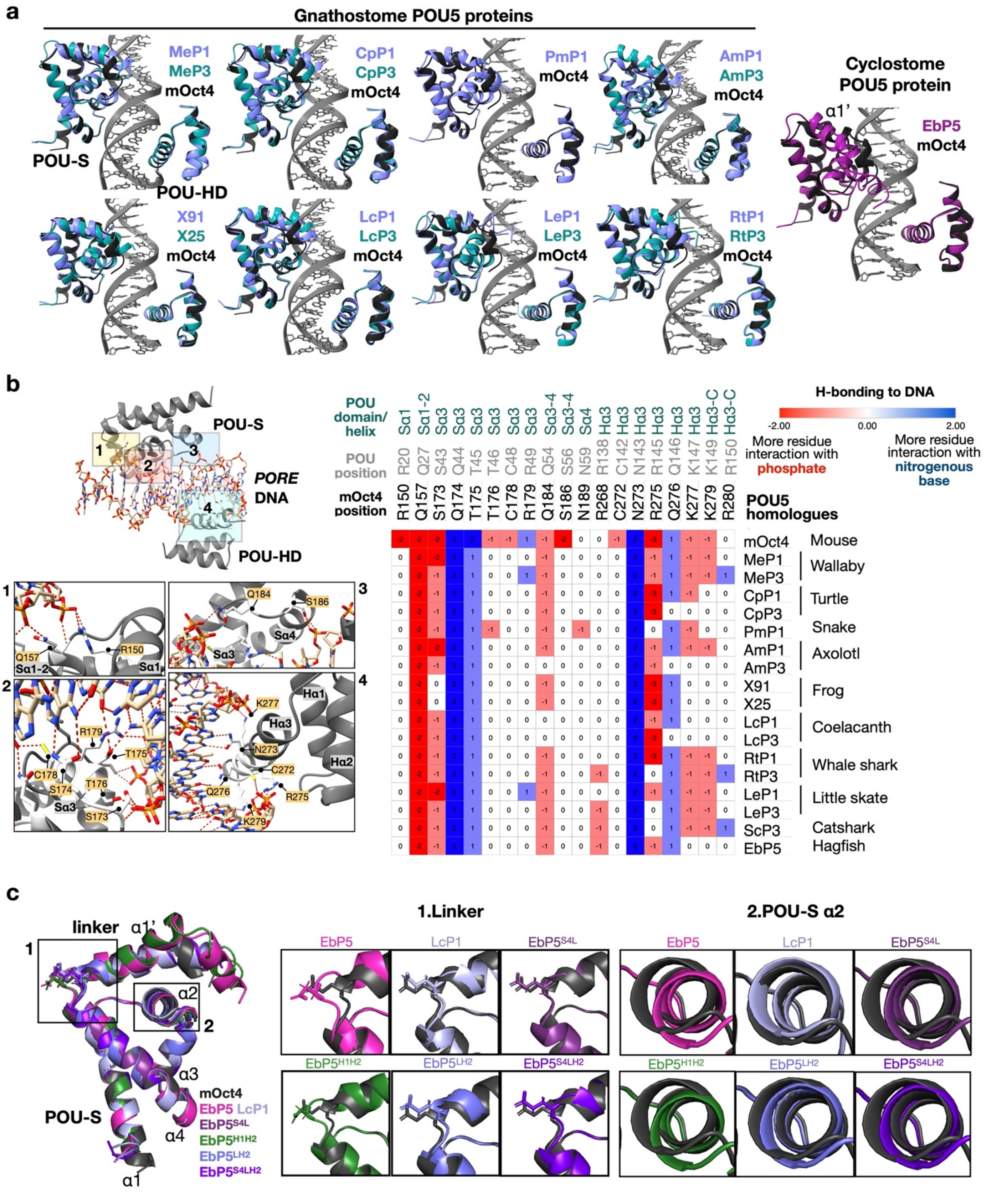
a
Gnathostome POU5 proteins
MeP1
MeP3
mOct4
CpP1
CpP3
PmP1
AmP1
AmP3
POU-S
POU-HD
X91
X25
LcP1
LcP3
LeP1
LeP3
RtP1
RtP3
Cyclostome POU5 protein
α1'
EbP5
b
POU-S
PORE DNA
POU-HD
H-bonding to DNA
More residue interaction with phosphate
More residue interaction with nitrogenous base
POU domain/ helix
POU position
mOct4 position
POU5 homologues
Mouse
Wallaby
Turtle
Snake
Axolotl
Frog
Coelacanth
Whale shark
Little skate
Catshark
Hagfish
ScP3
c
linker
POU-S
1.Linker
2.POU-S α2
EbP5
LcP1
EbP5S4L
EbP5H1H2
EbP5LH2
EbP5S4LH2

Supplementary Fig. 14, related to Fig. 7. AlphaFold2-based structural models for POU5 proteins used in Oct4 rescue assays.

a, Predicted structures for all gnathostome and cyclostome POU5 homologues analysed for rescue potential in OCT4-null ESCs (Figures 2 and 6) using AlphaFold2. The POU-S domain together with the linker helix (POU-S-L) and the POU-HD of each pair of POU5 homologues are shown in superimposition to mOct4 (black) on the *PORE* DNA element (3L1P [https://www.ncbi.nlm.nih.gov/Structure/pdb/3L1P]) and visualized by ChimeraX. **b**, POU-DNA H-bond interactions were predicted by ChimeraX. The number of H-bonds from each residue interacting with nitrogenous base (blue, positive value) and/or phosphate (red, negative value) of the DNA are shown as a heatmap, produced by Morpheus[15]. Zoom-ins on the mOct4-*PORE* interaction regions to visualize specific residues (boxes 1-4). **c**, AlphaFold2 predicted structures for chimeric proteins. Superimposition of POU-S domains from mOct4, LcP1, EbP5 and the four chimeras (left side), with zoom-in 1 focusing on the $E208^{(POU78)}$ residue found altered in the bend between the POU-S $\alpha 4$ and the Linker $\alpha 1'$ and the zoom-in 2 focusing on the positioning of POU-S $\alpha 2$, each structure is compared to mOct4 (dark grey).

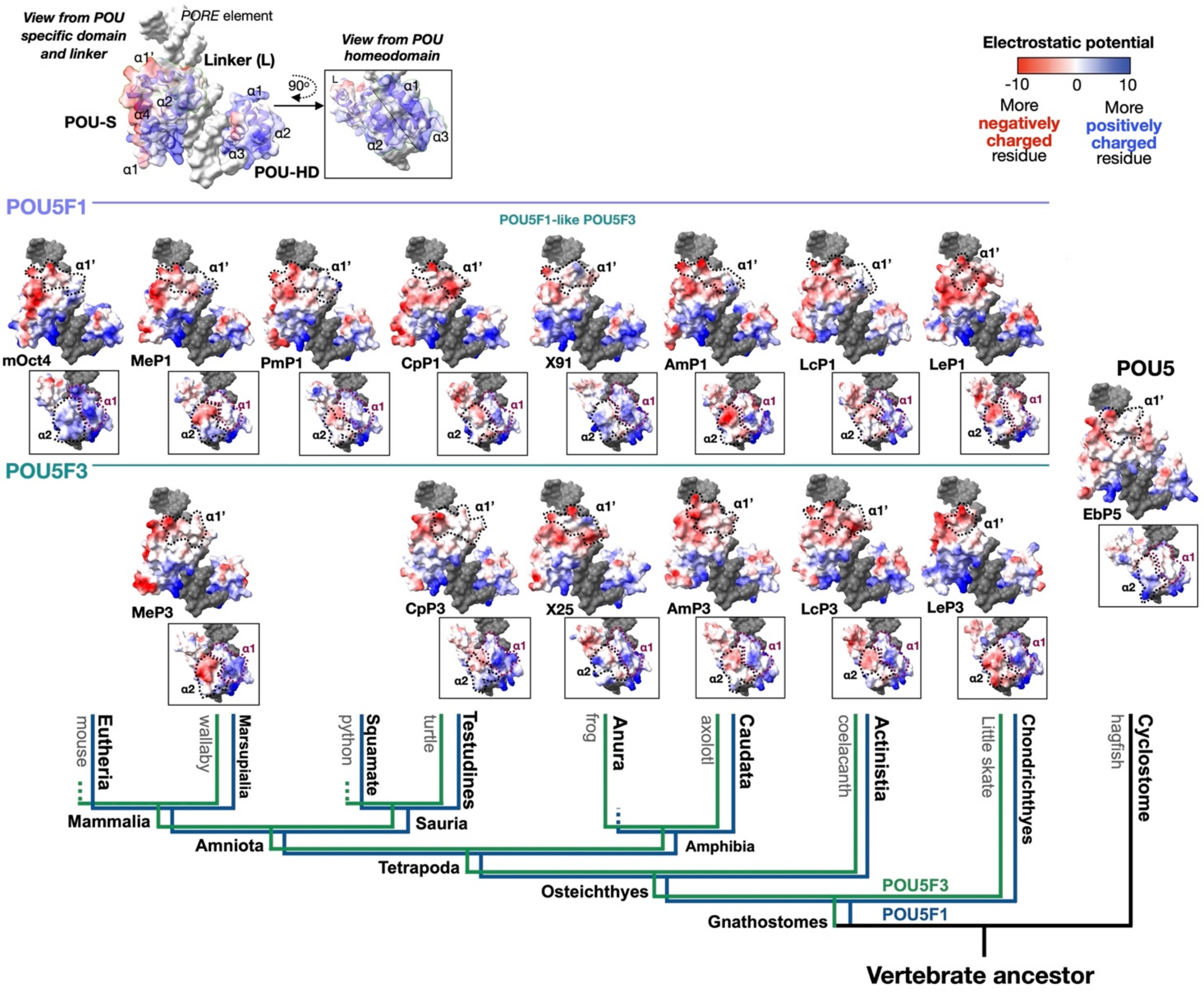

Supplementary Fig. 15, related to Fig. 7. Electrostatic potential of all AlphaFold2-based POU5 structural models.

Predicted electrostatic surface potentials of POU5 domains on the *PORE* DNA element for all gnathostome and cyclostome POU5 homologues analysed. Surface charges, determined by ChimeraX, with negatively charged areas shown in red and positively charged in blue. Each POU5 protein has an orientation focusing on the POU-S-L (top) and the POU-HD (boxed below) with a description of the views above. The structures are organized based on the phylogeny tree shown at the bottom.

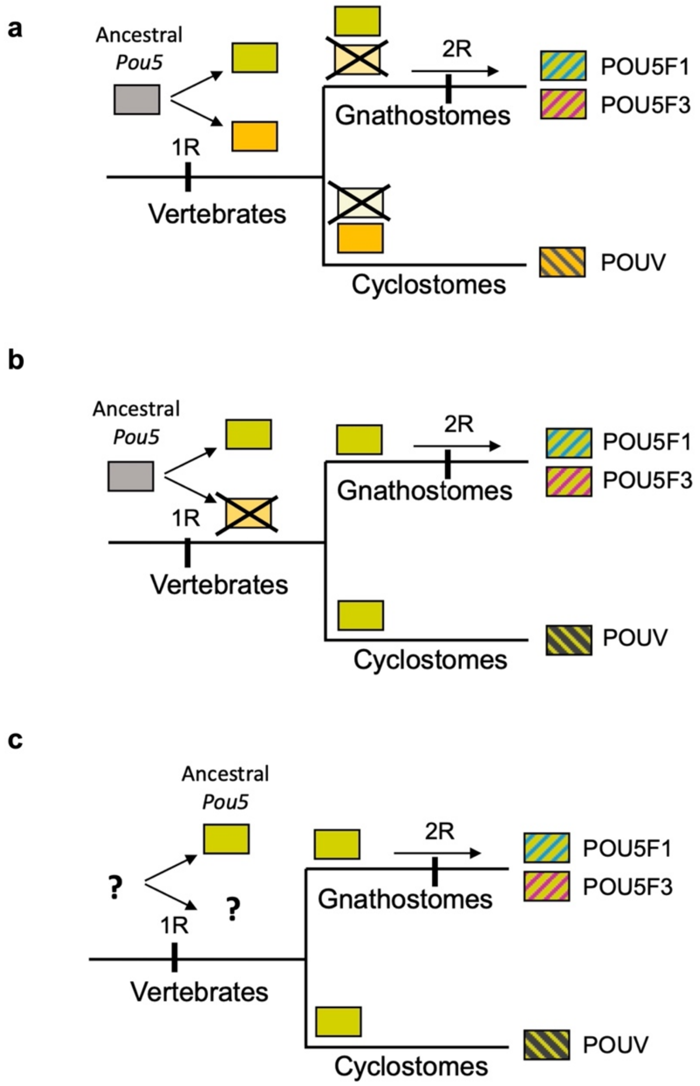

Supplementary Fig. 16, related to Fig. 9. Evolution of the POU5 family prior to the gnathostome radiation.

Our data suggest that the *Pou5f1*/*Pou5f3* duplication was part of the second round of vertebrate WGD (whole genome duplications), recently proposed to have followed the split between gnathostomes and cyclostomes[16]. This hypothesis is consistent with both the detection of extensive syntenies found between the paralogous loci and the monophyly of gnathostome *Pou5* genes, POU5F1 and F3 proteins appearing more similar to each other than to their cyclostome counterparts. Different possibilities consistent with these observations are shown. **a**-**b**, Early rise of *Pou5* genes in the vertebrate lineage, prior to the first round of WGD (1R) with (**a**) differential losses of either one of 1R duplicates between gnathostomes and cyclostomes or (**b**) loss of one 1R duplicate prior to the gnathostome-cyclostome split. **c**, variation of **b**, with the rise of *Pou5* genes following the 1R WGD.

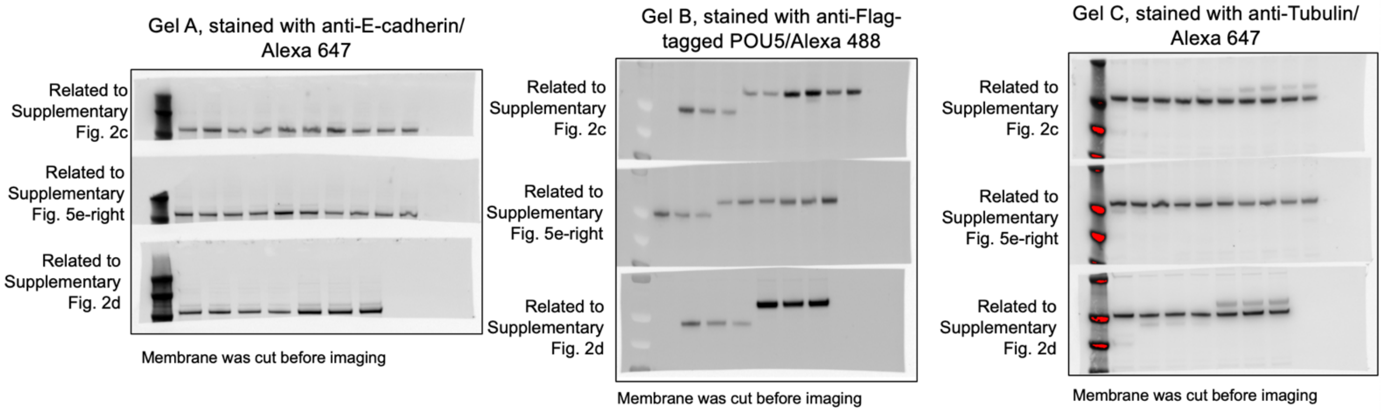

Supplementary Fig. 17. Uncropped western blot images, related to Supplementary Fig. 2c-d and 5e-right.

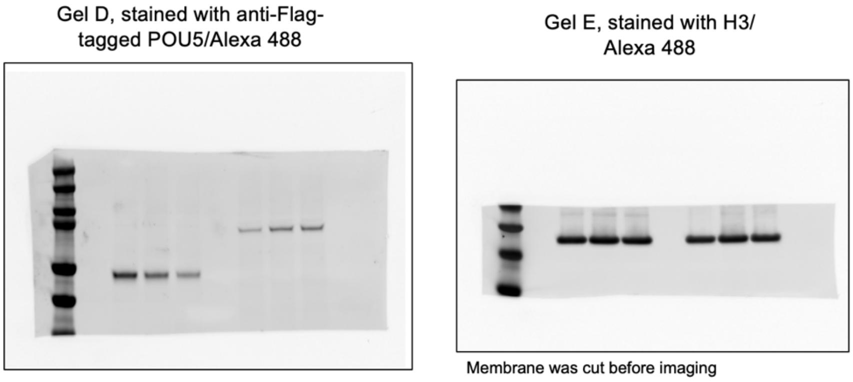

Supplementary Fig. 18. Uncropped western blot images, related to Supplementary Fig. 5e-left.

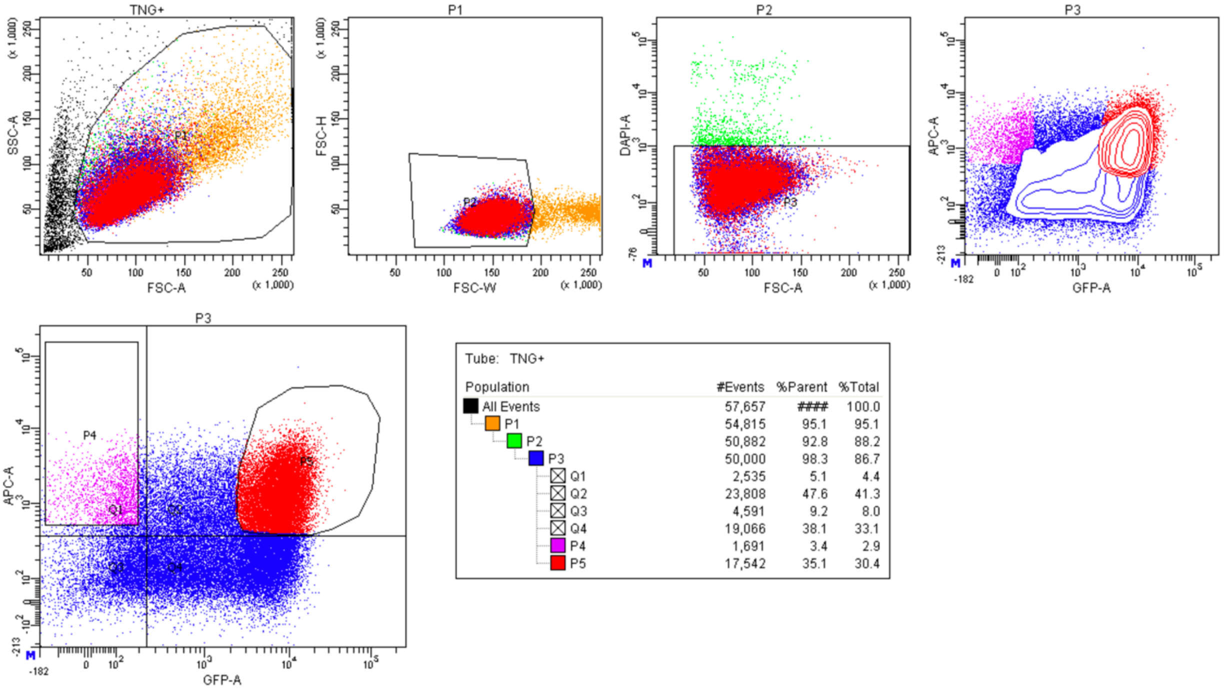

Population	#Events	%Parent	%Total
All Events	57,657	####	100.0
P1	54,815	95.1	95.1
P2	50,882	92.8	88.2
P3	50,000	98.3	86.7
Q1	2,535	5.1	4.4
Q2	23,808	47.6	41.3
Q3	4,591	9.2	8.0
Q4	19,066	38.1	33.1
P4	1,691	3.4	2.9
P5	17,542	35.1	30.4

Supplementary Fig. 19. Flow cytometry gating strategy. Nanog-GFP iPSC lines were induced by different Oct4 homologues and stained with c-KIT-APC. FSC-A/SSC-A gates were set to select cell population of interest and exclude cell debris. FSC-H/FSC-W gates were set to select single cell population. DAPI-A/FSC-A gates were used to select only live cells and exclude dead cells (high DAPI). This DAPI low/negative gates were then used to determine cell populations with/without c-KIT-APC and Nanog-GFP. Boundary between positive and negative cell populations were set using unstained E14Tg2A ESCs.

Supplementary Note 1, related to Fig. 1. Parameters used for the estimation POU5 protein evolutionary rates.

For the estimation of protein evolutionary rates, XML files were generated from input protein sequences using BEAUTi (v1.10.3) and the parameters indicated below (1-6). Species phylogeny with a monophyly of gnathostome POU5F1 and POU5F3 groups was imposed as shown in Supplementary Fig. 3a, except for the position of cyclostomes (Fig. 1d), which was left unconstrained. POU5 protein sequences were subjected to further process using BEAST. A plateau in the results was observed starting from 1 million iterations and we ran 9 million additional iterations for a total of 10 million iterations to ensure that convergence was reached. We used online platform CIPRES SCIENCE GATEWAY (https://www.phylo.org) for BEAST analysis (ref.17). Two outputs from BEAST analysis (.log and .tree file) were analysed for statistical outcomes of the phylogenetic tree. We constructed and visualized phylogenetic trees using TreeAnnotator v.1.10.3 and FigTree (v.1.4.2). The rate of protein evolution was visualized as colour gradient in FigTree.

1) Substitution Model: JTT (ref.18)
2) Site heterogeneity Model: Gamma
3) Number of Gamma Categories: 4
4) Clock Model: Lognormal relaxed clock (Uncorrelated)
5) Tree Prior shared by all tree models: Coalescent Model (Fixed)
6) MCMC: Length of chain:10,000,000, Echo state to screen every: 10,000, Log parameters every: 10,000

Supplementary Note 2, related to Fig. 6b. Additional details regarding catshark embryo analysis.

Expression of *ScPou5f1* and *ScPou5f3* during catshark early development:

The catshark develops from a telolecithal egg and exhibits a protracted development, gastrulation movements starting at stage 11, seven to ten days after blastocoel formation, which itself takes place at the time of egg deposition. Prior to gastrulation (blastocoel stage to stage 10), the blastoderm consists of two cell populations, a deep mesenchymal one of endodermal identity, and a superficial, epithelial-like layer, expressing *Sox2* and *Bmp4* in opposite, largely complementary territories[19]. At these stages, both *ScP1* and *ScP3* show similarly strong, uniform expression in the superficial cell layer, including the blastoderm margin but excluding the deep mesenchyme (Fig. 6b i-ii, ii^{1}, viii-ix). Expression restricted to the superficial layer including the margin is maintained for both paralogues at stage 11, when involution starts posteriorly (Fig. 6b iii, x, x^{1}). When the embryonic axis becomes morphologically visible, at the posterior pole of the blastoderm (stage 12), the signal intensity decreases in a sector located anteriorly to the forming neural plate, while increasing posteriorly, at the level of the forming posterior arms where mesoderm internalisation takes place (Fig. 6b iv, xi). During axis elongation (stage 13-14), the transcripts become confined to the posterior part of the embryonic axis, including the posterior arms but excluding anterior territories, which start to express lineage specific markers (Fig. 6b v-vi, v^{1}, v^{2}, xii-xiii, xiii^{1}). The expression profiles of the two paralogues segregate at the time of neural tube closure (Fig. 6b vii, xiv). From stage 15 to 19, *ScP1* expression is consistently observed in the posterior lateral mesoderm, at the site expected for primordial germ cells (PGCs) (Fig. 6b vii, vii^{1}, vii^{2}), while *ScP3* only exhibits a faint and transient signal at this location at stage 15. *ScP3* expression sites not shared by *ScP1* are also observed at these stages, in the posterior part of the head enlargement at stage 15 (Fig. 6b xiv, xiv^{1}), in the anterior hindbrain, with two bands of expression at stage 16 (Fig. 6b xv, xv^{1}), and at the level of the tail bud at stages 16 to 19 (Fig. 6b xv, xv^{2}, xvi, xvi^{1}).

Additional Fig. 6b legend:

Whole-mount views of catshark embryos following in situ hybridisations with probes for *ScPou5f1* (*ScP1*) (**i-vii**) or *ScPou5f3* (*ScP3*) (**viii-xvi**). **i-iii** and **viii-x** show dorsal views of the blastoderm at blastula (**i, viii**) and epiboly (**ii, ix**) stages, and at the onset of gastrulation (stage 11; **iii, x**). **iv-vii** and **xi-xiv** show dorsal views of the elongating embryo at stage 12 (**iv, xi**), stage 13 (**v, xii**), stage 14 (**vi, xiii**), stage 14+ (**xiv**) and stage 15 (**vii**). **xv** and **xvi** show lateral views of stage 19 and 20 embryos respectively. **ii^{1}**, **v^{1}**, **v^{2}**, **x^{1}**, **xiii^{1}**, **xiv^{1}** and **xvi^{1}** show sections of the embryos presented respectively in **ii**, **v**, **x**, **xiii**, **xiv** and **xvi**, at the plane and level indicated by red dotted lines. **vii^{1}** shows enhanced magnification of the territory boxed in red in **vii**. **vii^{2}** shows a transverse section at the level of presumptive PGCs of a stage 19 embryo, submitted to whole-mount hybridisation with a *ScP1* probe. Red arrows in **iv-vi** and **xi-xiii** point to a signal at the posterior margin lining the posterior arms. Red arrowheads in **vii**, **vii^{1}**, **vii^{2}**, **xiv** point to signals observed at the expected location for PGCs. **xv^{1}** and **xv^{2}** respectively show magnifications of anterior hindbrain and tailbud territories boxed in red

in **xv**, where *ScP3* but not *ScP1*, is expressed. The red asterisk in **xvi**, **xvi**[1] and the red arrow in **xv**[1] and **xvi** respectively indicate *ScP3* signals in the tailbud and in the anterior hindbrain, not observed for *ScP1*.

Supplementary Note 3, related to Fig. 3b-c. CellProfiler Pipeline for immunostaining quantification.

#copy this text to .txt or .rtf file#

```
Version:5
DateRevision:413
GitHash:
ModuleCount:7
HasImagePlaneDetails:False

Images:[module_num:1|svn_version:'Unknown'|variable_revision_number:2|show_window:False|notes:['To begin creating your project, use the Images module to compile a list of files and/or folders that you want to analyze. You can also specify a set of rules to include only the desired files in your selected folders.']|batch_state:array([], dtype=uint8)|enabled:True|wants_pause:False]
    :
    Filter images?:Images only
    Select the rule criteria:and (extension does isimage) (directory doesnot containregexp "[\\\\/]\\.")

Metadata:[module_num:2|svn_version:'Unknown'|variable_revision_number:6|show_window:False|notes:['The Metadata module optionally allows you to extract information describing your images (i.e, metadata) which will be stored along with your measurements. This information can be contained in the file name and/or location, or in an external file.']|batch_state:array([], dtype=uint8)|enabled:True|wants_pause:False]
    Extract metadata?:No
    Metadata data type:Text
    Metadata types:{}
    Extraction method count:1
    Metadata extraction method:Extract from file/folder names
    Metadata source:File name
    Regular expression to extract from file name:^(?P<Plate>.*)_(?P<Well>[A-P][0-9]{2})_s(?P<Site>[0-9])_w(?P<ChannelNumber>[0-9])
    Regular expression to extract from folder name:(?P<Date>[0-9]{4}_[0-9]{2}_[0-9]{2})$
    Extract metadata from:All images
    Select the filtering criteria:and (file does contain "")
    Metadata file location:Elsewhere...|
    Match file and image metadata:[]
    Use case insensitive matching?:No
    Metadata file name:None
    Does cached metadata exist?:No

NamesAndTypes:[module_num:3|svn_version:'Unknown'|variable_revision_number:8|show_window:False|notes:['The NamesAndTypes module allows you to assign a meaningful name to each image by which other modules will refer to it.']|batch_state:array([], dtype=uint8)|enabled:True|wants_pause:False]
    Assign a name to:All images
    Select the image type:Color image
    Name to assign these images:nuclei
    Match metadata:[]
    Image set matching method:Order
    Set intensity range from:Image metadata
    Assignments count:1
    Single images count:0
    Maximum intensity:255.0
    Process as 3D?:No
    Relative pixel spacing in X:1.0
```

Relative pixel spacing in Y:1.0
Relative pixel spacing in Z:1.0
Select the rule criteria:and (file does contain "")
Name to assign these images:DNA
Name to assign these objects:Cell
Select the image type:Grayscale image
Set intensity range from:Image metadata
Maximum intensity:255.0

Groups:[module_num:4|svn_version:'Unknown'|variable_revision_number:2|show_window:False|notes:['The Groups module optionally allows you to split your list of images into image subsets (groups) which will be processed independently of each other. Examples of groupings include screening batches, microtiter plates, time-lapse movies, etc.']|batch_state:array([], dtype=uint8)|enabled:True|wants_pause:False]
Do you want to group your images?:No
grouping metadata count:2
Metadata category:None
Metadata category:None

ColorToGray:[module_num:5|svn_version:'Unknown'|variable_revision_number:4|show_window:True|notes:[]|batch_state:array([], dtype=uint8)|enabled:True|wants_pause:False]
Select the input image:nuclei
Conversion method:Combine
Image type:RGB
Name the output image:OrigGray
Relative weight of the red channel:1.0
Relative weight of the green channel:1.0
Relative weight of the blue channel:1.0
Convert red to gray?:Yes
Name the output image:OrigRed
Convert green to gray?:Yes
Name the output image:OrigGreen
Convert blue to gray?:Yes
Name the output image:OrigBlue
Convert hue to gray?:Yes
Name the output image:OrigHue
Convert saturation to gray?:Yes
Name the output image:OrigSaturation
Convert value to gray?:Yes
Name the output image:OrigValue
Channel count:1
Channel number:1
Relative weight of the channel:1.0
Image name:Channel1

IdentifyPrimaryObjects:[module_num:6|svn_version:'Unknown'|variable_revision_number:14|show_window:True|notes:[]|batch_state:array([], dtype=uint8)|enabled:True|wants_pause:False]
Select the input image:OrigGray
Name the primary objects to be identified:IdentifyPrimaryObjects
Typical diameter of objects, in pixel units (Min,Max):15,50
Discard objects outside the diameter range?:No
Discard objects touching the border of the image?:No
Method to distinguish clumped objects:Intensity
Method to draw dividing lines between clumped objects:Intensity
Size of smoothing filter:10
Suppress local maxima that are closer than this minimum allowed distance:7.0
Speed up by using lower-resolution image to find local maxima?:Yes

```
    Fill holes in identified objects?:After declumping only
    Automatically calculate size of smoothing filter for declumping?:Yes
    Automatically calculate minimum allowed distance between local maxima?:Yes
    Handling of objects if excessive number of objects identified:Continue
    Maximum number of objects:500
    Display accepted local maxima?:No
    Select maxima color:Blue
    Use advanced settings?:Yes
    Threshold setting version:12
    Threshold strategy:Global
    Thresholding method:Otsu
    Threshold smoothing scale:1.3488
    Threshold correction factor:1.0
    Lower and upper bounds on threshold:0.0,1.0
    Manual threshold:0.0
    Select the measurement to threshold with:None
    Two-class or three-class thresholding?:Three classes
    Log transform before thresholding?:No
    Assign pixels in the middle intensity class to the foreground or the background?:Foreground
    Size of adaptive window:50
    Lower outlier fraction:0.05
    Upper outlier fraction:0.05
    Averaging method:Mean
    Variance method:Standard deviation
    # of deviations:2.0
    Thresholding method:Minimum Cross-Entropy

MeasureObjectSizeShape:[module_num:7|svn_version:'Unknown'|variable_revision_number:3|show_window:True|notes:[]|batch_state:array([], dtype=uint8)|enabled:True|wants_pause:False]
    Select object sets to measure:IdentifyPrimaryObjects
    Calculate the Zernike features?:Yes
    Calculate the advanced features?:No
```

#copy this text to .txt or .rtf file#

Supplementary Table 1. Protein sequences used for AlphaFold2 structural modelling

Oct4 homologues	Protein sequences and ModelArchive Accession code
**mOct4**	UniProt : P20263<br>[https://alphafold.ebi.ac.uk/entry/P20263]
**MeP1**	MAGHLAPEYFSPPPGGGGAGSSEPTWNWPGFQGPPSGAGAGPGPDGWGMAPYHPLYDMWGGGVYCEPQPNVGVMAQPAQEIAVPDGEA<br>GPGVESPSEDSSPESRATVRVTKIEPVESGEEQPQETPSPEELEQFAKELKRKRITLGYTQADVGITLGALFGKVFSQTTICRFEAQQ<br>LSFKNMCKLRPLLQKWLEAADDNDHLQELCKAETVLQQARKRKRTSIENGVRGNLETMFLQCPKPTLQQISNIAEELGLEKDVVRVWF<br>CNRRQKGKRSNSNSPREDFEAAGSFPGGPMPFPLAPGPPPFSHPSYGGPHFTALYSPSPFTEGDAFSSLPVTPLGSAMHSS<br>[https://www.modelarchive.org/doi/10.5452/ma-eym79]
**MeP3**	MPDGSSQYGKGGYIGGSTPRPSQPQPSSQSFFTFPSAVKSEYSLEGPSTPDSSQTKGWYAFSAPPPPAEAGQASASHNLHLMPSKEED<br>SSCSSPSSSSSGAPERDLRETVVPEAKYCAHPGPTYYAPAWTGPFWPGLGSSGSVPTGALPGTPLPPHPLQALGTFPGPRPLYPASVQ<br>QPQPGLGSSGGTSSGSVSSSNVSSSVSSTSGSSGAASEEGIPSSDSGEEDTPTSEELELFAKELKHKRISLGFTQADVGMALGTLYGK<br>MFSQTTICRFEALQLSFKNMCKLKPLLQRWLQAVENTDNPQEMCSMEQVLAQARKRKRRTSIETSVKGTLEGFFRRCGKPTPQQICDL<br>AEELHLDKDVVRVWFCNRRQKGKRLLLPYGEDGEALPYELAPGTALVLPTAAVPQNYAAPPPPEPPALYMSTFPKGEPCIPGMPMPNG<br>GI<br>[https://www.modelarchive.org/doi/10.5452/ma-bfj5i]
**CpP1**	MAGHVGQELGRPYPLAGQGLHLDAVPPGPANGAPHEGPGSFLPEAYNGAAPGQGYGYRLDYGPQGGLLETQPGDPPGHQALLRAWCPF<br>PMGGEGWPPPYGPAQPAAAYEGLKPDVKTERECGQQGPMYGHPAQAWGGCFLPQATARPPAALPPPPAGPAGEGGEGSGASSPQSDGS<br>GGSPGAPAAPGEETPSTEEMEQFAKELKHKRITLGFTQADVGLALGVLYGKMFSQTTICRFEALQLSFKNMCKLKPLLQRWLDEADGN<br>ANLQEMCSMESALLQARKRKRTSIETAARGSLESYFLRCPKPSLQEIAHIAHDLHLDKDVVRVWFCNRRQKGKRSGGCSVRDDCEGGA<br>LPFAPPAQLPGPPMGHHPPPPQGYNAAAFATLYVPQFHHGGEPFDPTPPGPPLMGHPMHST<br>[https://www.modelarchive.org/doi/10.5452/ma-fgbzx]
**CpP3**	MFSQEALPAASFSLGAGILQDASGQFGKSSYSSSPQPPPFFPFPAVKAEYEPPELQAGEAKPWCPFPGPEPCHQPGMAGGHQGPPLLG<br>SGQAPKEEPGQEKGRRQGSPEAKCALLPAGPYYAHPWSSPFWPALAGHSAAAARGPLPSQTFPGVGLCPGAHHAIYPSDPHQPPSGLS<br>SLGSSGSSSGAASEGGQSSESGDEDATPTSEELEQFAKDLKHKRITLGFTQADVGMALGTLYGKMFSQTTICRFEALQLSFKNMCKLK<br>PLLQRWLNEVENSDSLQELCNAEQVLAQARKRKRRTSIENNVKGTLESFFRKCIKPSPQQISQIAEDLNLDKDVVRVWFCNRRQKGKR<br>LLLPFGDENEGAMYDMNLALAHPALPAAVSSYAPAPLASPPPIYMSAFHKGEICPQALQPAVSMGNSGN<br>[https://www.modelarchive.org/doi/10.5452/ma-qu7g5]
**PmP1**	MAGHLRRELGRPYSFPVQGLHLTTLPAVPQHETSGVFLPDAFSGATPGQAFGFKPDYGSQGGIVEPYPGGGEVSHSWYPFPAGGEAWN<br>PPPGVVMGPYAVPQPGEACQGSKPDIKVERDFDQPGPYYGGGQPWAGACLVPPAAARPASCSSTKEAASPSPDPQPSSSPASQVEEAD<br>SADSSPRSDASQSPSEQMASEEVKEEAASGDEETTTSVELEQFAKELKHKRITLGFTQADVGLALGLLYGKMFSQTTICRFEALQLSF<br>KNMCKLKPLLQRWLQEADSNENLQEQLCSMESAMIQARKRKRTSIENNVRGSLESYFLRCPKPNLQEISQIADDLSLEKDVVRVWFCN<br>RRQKGKRNPGPGPRDECDGPVLPQVCPHGPLQAPGLGPAHHQLPPPAQGYNTTAFAAVYLPQFHEGDAFVPPTSSSAVMGHPMHSS<br>[https://www.modelarchive.org/doi/10.5452/ma-e7fwh]
**AmP1**	MAGHLGQEIGRAAYGFGAQALHLGAGGLEAGGPGFLSESYGPYAGFKALEYAHGGAEGEGRPGAHGLARAWYPFSEAWGPVYGQSGAG<br>AGFESSRVEVKVERPDKEAGYGQQHQQAWAGYFVPQLAVPARSPASVASGGQVPAAPASPSDDSPHSSTASSSSASPDLGAGGAPRDL<br>DSGDEEGGTSADLEQFAKELKQKRITLGFTQADVGLALGALYGKMFSQTTICRFEALQLSFKNMCKLRPLLQRWLVEADTNENLQELC<br>NLENALQQARKRKRTSIENSVKDNLEAFFLKCPKPTHQEIAHISEDLNLEKDVVRVWFCNRRQKGKRSICREEYDGFQQYPGMQPGPP<br>ALSHLPTSYIAQGYNGAAAAFAAVYMQPFHDSEMYSQTVSRHLHSN<br>[https://www.modelarchive.org/doi/10.5452/ma-bmjpb]
**AmP3**	MLGRDTVTRDPSPTFAICRTGGAMYSQDPRAPIPMNIQEGSCQATFPGLPRPRPPAQSNTRPLGHPQPFLPFPGVKTPYGTCERAGGG<br>VEPEQARPWHPFQVPEALGPPGISVGHQVDRLGEVRREFKEEPQAEDGCRQGSPEGTYSPPVPTYGGPYYPQPWNGSFWPALGGTGST<br>ANCSPGSAIPVPPGLYPSPLNQSSSGVSSLGSSSSEATSEGGLSSDSGDEDTPTNEELEQFAKALKHKRITLGFTQADVGLALGSLYG<br>RMFSQTTICRFEALQLSFKNMCKLKPLLQRWLNEAENTDNMEELCNMEQMLAQARKRKRRTSIENNVRGTLESFFLKCSKPGPQEISQ<br>IAEDLSLDKDVVRVWFCNRRQKGKRLLLPFVEEMEGGGMYETNQAMAHPGGAPFTLPTMISSQGYPVSSLNSQTLYMTAFHKTEMFPQ<br>ALHPGVPLGNSIS<br>[https://www.modelarchive.org/doi/10.5452/ma-aaehw]
**X91**	MYNQQTYPSFTHNPALMPDGSGQYNLGTYTGMARHPHQAQAFFPFSGVKSDYGDLGGQTTSVGDTSAWNPLTSLDSANQLGISGQGNP<br>FKNLKREREDDEEKSESPEPKCSPPSLPPAYYTHAWNPTTTFWSQVSSSGTTVVSKPLPTPLQPGDKCDPVEANKIFTSSPDKSGESG<br>ISSLDNSRCSSATSSSSGGTNVGTPRSLSRGASDGLSSDSEEEAPNSGEMEQFAKDLKHKRITMGYTQADVGYALGVLFGKTFSQTTI<br>CRFESLQLSFKNMCKLKPLLRSWLHEVENNENLQEIISRGQIIPQVQKRKHRTSIENNVRCTLENYFMRCSKPSAQEIAQIARELNME<br>KDVVRVWFCNRRQKGKRQVYPYIRENGGEPYDTPQTLTPPSQGPFPLPQVMPSQVFPTVPLGANPTIYAPTYHKNDMFPQAMHHGIGM<br>GNQGN<br>[https://www.modelarchive.org/doi/10.5452/ma-gmf2j]
**X25**	MYSQQPFPAFAFNAGLMQDPANCHFGGYTGLGHPQPFSFAFSTLKSENGESGVQGMGDCTTPVMPWNSLASFDHQVQMENNQQGNPPR<br>APSPTLSDSRIKVKEEVVHETDSGEESPEPKYPSPPNPSLYYPNAWTGAPFWQVNPTPGNNINPMPNQTLVKNTSLPGNTTYPTPANQ<br>SPNTPVDCVASSMESSRCSSTNSPNGAINERATTIPNGEMLDGGQSSDNEEEVPSESEMEQFAKDLKHKRVSLGYTQADVGYALGVLY<br>GKMFSQTTICRFESLQLSFKNMCQLKPFLERWVVEAENNDNLQELINREQVIAQTRKRKRRTNIENIVKGTLESYFMKCPKPGAQEMV<br>QIAKELNMDKDVVRVWFCNRRQKGKRQGMPTVEENDGEGYDVAQTMGSPPVGHHALQQVVTPQGYMAAPQIYASAFHKNDLFPQTVPH<br>GMAMGGHIG<br>[https://www.modelarchive.org/doi/10.5452/ma-0ajir]
**LcP1**	MAGHLGQDVGRSYGMCPEVFQVSGGTRQEASSVLGADGASLFSPPGFAFKEEYGHAAVDTQFGDPATMAGAHALSRPWYPFPAAEAWA<br>HGAIMTGQHSTQAPHSSHDIHKVHIKTEEEGRSEAKTSTPVSQGVLHGHPLWSPRFLPQVPSGGLVANSLVSARTLGGHGYWASQQAT<br>QSRPPTSPVSGKSSPSDQPTSPENAEENHRDALTDSGTEDTPTTDDLEQFAKELKHKRISLGFTQADVGLALGALYGKMFSQTTICRF<br>EALQLSFKNMCKLKPLLQRWLDEADTNENLQELCNLEQVLSQARKRKRTSLETTAKGTLESFFLKCSKPSLQEIAQIAEELSLDKDVV<br>RVWFCNRRQKGKRSLYQSEEEYETPPQYGVHPFQPPVPTMHLPNSVVQQAYNGTPFPTTLYVPQFYEGEAFPQPRAGPCTRNEFWDST<br>PFSTSF<br>[https://www.modelarchive.org/doi/10.5452/ma-kpwra]
**LcP3**	MSDRSTTPNQDGSRVCRPIYPQEGLSSLNLGNGMLQDSSSQFSKSNYNGISPQPFFPFPPMKADYGQIMELQVGDSGHSRHWYAFPAP<br>DLSSQLALGGQQAGGHSSQLGEIRDPAKVEIKQEKESPEPKYVSPPAAAAIAAGAYYAHPWNNSFWPALTSSSATTGSSNPIPGQAFP<br>GIGVFPGTSAQLYPTALNHTSSGVSSLASSSSSSSGAGSEEGQSSDSADEDNPTTEELEQFAKELKHKRITLGFTQADVGLALGTLYG<br>KMFSQTTICRFEALQLSFKNMCKLKPLLQRWLNEAENNENLQEMCNIEQVLAQARKRKRRTSIENNVKGTLENYFLKCPKPTSQEISQ<br>IADDLNLEKDVVRVWFCNRRQKGKRLAFPFGEEAEGISYDVNPALHAPSTSSMTLHGPVVTQGYPGTTLTAPPPVYMPAFHKSEVFPQ<br>ALHPGVAMTNHTT<br>[https://www.modelarchive.org/doi/10.5452/ma-4pjaj]

**RtP1**	MNTAVCAEPLGHRAVPEPSSQATLPAPPAFPLPGDYRLRQFREPSSRMATPWYPESWAPGPPSSSEEEPRRRAQPQVPEVKSPSPALH<br>AGWRSPLPAPSCCFLPPAGYPLPGQPALCPQQEHPARDPPGEQPRSRSRSPGRPQEEEEEAAAGAGSPPDTEGEETPTSEDLKHFAKK<br>LKKKRIIMGFTQAEVGLALGALYGKMFSQTTICRFEALQLSYKNMCKLRPLLQRWLEEAKDNENFQELCSMEQRLATARKRKRRTSID<br>SNVKGLLEAAFIKCPKPSAQEIIQIADDLNLEKDVVRVWFCNRRQKGKRALFHGGEDSEGVPGYGPLPMQHAPLGLPSPLAPHQGYVG<br>EAIGTVYTPHFHKGSLYRQQPAPAPGTMHSS<br>[https://www.modelarchive.org/doi/10.5452/ma-zvoeb]
**RtP3**	MSISPGQGANGAKSIQSPERAALVPFGNGVVQDVGAQFYKPGYNGLSAQYLFPFPPLKGEYGHSESPLGDCAAVSPSGYWYPFPGAEP<br>VPTHGAGHNSQVAVAGGYINRPEIKTEKESRGYPQEVRYSSPSGPGPSYNSRWSSPFWQPALAASSSNSSPGSSSSSGPLPSQSYGPF<br>PSPPQLYPSPSQHSDSSQPLQSNTGSTGTASEEGQSSDSEEEYPTKEKMEQFAKELKHKRITLGFTQADVGLALGNLYGKMFSQTTIC<br>RFEALQLSFKNMCKLKPILQRWLNDAENNGSLHEICNVEQVLDQSRKRKRRTSIENGVKRNLETYFMKCPKPTSEEISQIAEDLCLDK<br>EVIRVWFCNRRQKGKRMTLPCMEENDVQIPEGSPLHMSPGALMLPEPTVTQGYSAPMVPPPMYMSPFPQALHPAVSMGNHPS<br>[https://www.modelarchive.org/doi/10.5452/ma-hnxlj]
**LeP1**	MTSRIQHEIHRSFPPLFHDPRQLQSQEMSSQLAPERPSIVQGLHCGPAFRGDCQGHMAPEYRLGEPGTAGVSPHLPRPWYPFPAPDHW<br>PHSSVVARYPNAPPAPGREEDEKGTEAKFLPSLYNSPWSSCYLPQLPAAPSPPTARTAAGLQNQGHSPSSDHRSQPASPQMQPNNPSP<br>SPSADHDTKWVAPDGGNEEYPTKEKMEQFAKELKHKRITLGFTQADVGLALGNLYGKMFSQTTICRFEALQLSYKNMCKLKPLLQRWL<br>EEAKDSDNFQELCSIEQTLASSRKRKRRTSIDNNVKEGLENFFTVCPKPSTQEITKIADDLNLEKDVVRVWFCNRRQKGKRAAFQYGD<br>EFVELAGYGMPPLQPPLPGGFGAQGYNAAAISAAALYVPQADSYHCSEAAQAAPVARTMHSS<br>[https://www.modelarchive.org/doi/10.5452/ma-irupv]
**LeP3**	MSGTLSPVHGPPVTHRVNQSPESAALPAFSSGVVQDMGSQMYKPGYSSMGGQYLFPLGPRKSEYGGCDSAALPHPGYWHQPPVAEPPS<br>TAHWPQGHPHSGHSAHIKVEKDGFSSPPHPSYPHRWCGPGFWPPVPGSSSPASGSGQPCPGYSSFPSPPHMFPSPSQLSDSNPATQSI<br>AGSTGNTSEEGQSSDSEEEYPTKEKMEQFAKELKHKRITLGFTQADVGLALGNLYGKMFSQTTICRFEALQLSFKNMCKLKPILQRWL<br>NDAESDGGIHEICNVEQVLDQSRKRKRRTSIENGVKNNLETYFMKCPKPTSEEISQIAEDLRLDKEVIRVWFCNRRQKGKRMTLPCME<br>ENELQINEESPIRLSPGSLMPDPCVPQGYVMPPHMYMSPFPQPLHPAVTMGNHSS<br>[https://www.modelarchive.org/doi/10.5452/ma-3q6yf]
**ScP3**	MSARPGQGTSVSRSVPSPERPPVLSFGNGVVPDVSPQFYKPGYNAISAQYLFPFPGLKGEYGHSETQLGDCAAVSHTGYWYPFTDPAA<br>AHGSGHSSGHPTQLSAPGGLLRPEIKTEKECKGYPQEGRYISPTAPAPSYNHRWSTTFWQPALSSAANSTSSSSSSSSSAPLPSQTYS<br>AFGSFPSPPQMYPTNPSPSQQSDSSQPAHNTGSTGTTSEEGQSSDSEEEYPTKEKMEQFAKELKHKRITLGFTQADVGLALGNLYGKM<br>FSQTTICRFEALQLSFKNMCKLKPILQRWLNDAENNGGLHEICNVEQVLDQSRKRKRRTSIENGVKRNLETYFMKCPKPTSEEISQIA<br>EDLQLDKEVIRVWFCNRRQKGKRMTLPCMEENDVQIHEGSPLHMSPNALMLPDPIVTQGYSAAMVPPPMYMSPFPQALHPAVSMGNHT<br>S<br>[https://www.modelarchive.org/doi/10.5452/ma-l8ud8]
**EbP5**	MSSLSIKVDAANELQLMYESYSEASAAQSSVVRPYMEQAAQQRGACCVPGQGSGHGHVHLVPISPLSLGYSGELYEPSLQCPSRAHGA<br>VPGPAWYAYPGPSAAGSEAAQAAASWAAARGVEIKCEEGVGVGEHVESEVKRIRYQDFKYGAVYGSGYVLPNAAMHGGHGQQNPHSLS<br>HLNLHPHLQLNPSPHLQPNPNLHLQLNPNSHLQPSSHLQPNPSSHLQPNPNSHLQLSSHLQQNSHLQPSSHLQPNSHLQPNPNSHLQP<br>NPNSHLQPNTNSHLQPNPNSHLQPNPNSHLQPNPNSHLQLSSHSQQNPLSLLNVHPQAYPHSQSPAPTPLAAALASTQSPGASSGTED<br>GGCARERSRNSEHDLGSGDEGTISSETLAQFARDLKHKRITLGFTQADVGVALGSLYGRIFSQTTICRFEALQLSYRNMCKLQPLLER<br>WMIEAENADNVKEVCHLFLFNSSMDQSLANVNKLRRRRTTIENGVRDTLEAWYLVCSKPSAKEIARIAGELNLDKEVVRVWFCNRRQK<br>LKQLSPPLPKEEEPEMSSQHQLMPHIQEFGTLPTSSSSPAYDIHTYNMQASMNAPILPS<br>[https://www.modelarchive.org/doi/10.5452/ma-4n7bl]
**EbP5**^{S4L}	MSSLSIKVDAANELQLMYESYSEASAAQSSVVRPYMEQAAQQRGACCVPGQGSGHGHVHLVPISPLSLGYSGELYEPSLQCPSRAHGA<br>VPGPAWYAYPGPSAAGSEAAQAAASWAAARGVEIKCEEGVGVGEHVESEVKRIRYQDFKYGAVYGSGYVLPNAAMHGGHGQQNPHSLS<br>HLNLHPHLQLNPSPHLQPNPNLHLQLNPNSHLQPSSHLQPNPSSHLQPNPNSHLQLSSHLQQNSHLQPSSHLQPNSHLQPNPNSHLQP<br>NPNSHLQPNTNSHLQPNPNSHLQPNPNSHLQPNPNSHLQLSSHSQQNPLSLLNVHPQAYPHSQSPAPTPLAAALASTQSPGASSGTED<br>GGCARERSRNSEHDLGSGDEGTISSETLAQFARDLKHKRITLGFTQADVGVALGSLYGRIFSQTTICRFEALQLSFKNMCKLKPLLQR<br>WLDEADTNENLQELCNLEQVLSQARRRRTTIENGVRDTLEAWYLVCSKPSAKEIARIAGELNLDKEVVRVWFCNRRQKLKQLSPPLPK<br>EEEPEMSSQHQLMPHIQEFGTLPTSSSSPAYDIHTYNMQASMNAPILPS<br>[https://www.modelarchive.org/doi/10.5452/ma-zy6ha]
**EbP5**^{H1H2}	MSSLSIKVDAANELQLMYESYSEASAAQSSVVRPYMEQAAQQRGACCVPGQGSGHGHVHLVPISPLSLGYSGELYEPSLQCPSRAHGA<br>VPGPAWYAYPGPSAAGSEAAQAAASWAAARGVEIKCEEGVGVGEHVESEVKRIRYQDFKYGAVYGSGYVLPNAAMHGGHGQQNPHSLS<br>HLNLHPHLQLNPSPHLQPNPNLHLQLNPNSHLQPSSHLQPNPSSHLQPNPNSHLQLSSHLQQNSHLQPSSHLQPNSHLQPNPNSHLQP<br>NPNSHLQPNTNSHLQPNPNSHLQPNPNSHLQPNPNSHLQLSSHSQQNPLSLLNVHPQAYPHSQSPAPTPLAAALASTQSPGASSGTED<br>GGCARERSRNSEHDLGSGDEGTISSETLAQFARDLKHKRITLGFTQADVGVALGSLYGRIFSQTTICRFEALQLSYRNMCKLQPLLER<br>WMIEAENADNVKEVCHLFLFNSSMDQSLANVNKLRKRKRTSLETTAKGTLESFFLKCSKPSLQEIAQIAEELNLDKEVVRVWFCNRRQ<br>KLKQLSPPLPKEEEPEMSSQHQLMPHIQEFGTLPTSSSSPAYDIHTYNMQASMNAPILPS<br>[https://www.modelarchive.org/doi/10.5452/ma-4flec]
**EbP5**^{LH2}	MSSLSIKVDAANELQLMYESYSEASAAQSSVVRPYMEQAAQQRGACCVPGQGSGHGHVHLVPISPLSLGYSGELYEPSLQCPSRAHGA<br>VPGPAWYAYPGPSAAGSEAAQAAASWAAARGVEIKCEEGVGVGEHVESEVKRIRYQDFKYGAVYGSGYVLPNAAMHGGHGQQNPHSLS<br>HLNLHPHLQLNPSPHLQPNPNLHLQLNPNSHLQPSSHLQPNPSSHLQPNPNSHLQLSSHLQQNSHLQPSSHLQPNSHLQPNPNSHLQP<br>NPNSHLQPNTNSHLQPNPNSHLQPNPNSHLQPNPNSHLQLSSHSQQNPLSLLNVHPQAYPHSQSPAPTPLAAALASTQSPGASSGTED<br>GGCARERSRNSEHDLGSGDEGTISSETLAQFARDLKHKRITLGFTQADVGVALGSLYGRIFSQTTICRFEALQLSYRNMCKLQPLLER<br>WMIEAETNENLQELCNLEQVLSQARKRKRTSLETTAKGTLESFFLKCSKPSLQEIAQIAEELNLDKEVVRVWFCNRRQKLKQLSPPLP<br>KEEEPEMSSQHQLMPHIQEFGTLPTSSSSPAYDIHTYNMQASMNAPILPS<br>[https://www.modelarchive.org/doi/10.5452/ma-z7lnj]
**EbP5**^{S4LH2}	MSSLSIKVDAANELQLMYESYSEASAAQSSVVRPYMEQAAQQRGACCVPGQGSGHGHVHLVPISPLSLGYSGELYEPSLQCPSRAHGA<br>VPGPAWYAYPGPSAAGSEAAQAAASWAAARGVEIKCEEGVGVGEHVESEVKRIRYQDFKYGAVYGSGYVLPNAAMHGGHGQQNPHSLS<br>HLNLHPHLQLNPSPHLQPNPNLHLQLNPNSHLQPSSHLQPNPSSHLQPNPNSHLQLSSHLQQNSHLQPSSHLQPNSHLQPNPNSHLQP<br>NPNSHLQPNTNSHLQPNPNSHLQPNPNSHLQPNPNSHLQLSSHSQQNPLSLLNVHPQAYPHSQSPAPTPLAAALASTQSPGASSGTED<br>GGCARERSRNSEHDLGSGDEGTISSETLAQFARDLKHKRITLGFTQADVGVALGSLYGRIFSQTTICRFEALQLSFKNMCKLKPLLQR<br>WLDEADTNENLQELCNLEQVLSQARKRKRTSLETTAKGTLESFFLKCSKPSLQEIAQIAEELNLDKEVVRVWFCNRRQKLKQLSPPLP<br>KEEEPEMSSQHQLMPHIQEFGTLPTSSSSPAYDIHTYNMQASMNAPILPS<br>[https://www.modelarchive.org/doi/10.5452/ma-yye48]

Supplementary Table 2. Structural assessment of isolated POU domains

Oct4 homologues/ chimeric proteins		Structural assessment using MolProbity on Phenix			
		Isolated POU-S-L		Isolated POU-HD	
		Ramachandran favoured	Clash score	Ramachandran favoured	Clash score
**x-ray diffraction PDB: 3L1P** (Esch et al., 2013, ref[5])	**mOct4**	89.02	4.95	93.75%	0.00
**AlphaFold2** (in this study)	**mOct4**	98.90%	1.96	100.00%	0.00
	**MeP1**	100.00%	2.63	100.00%	0.00
	**MeP3**	98.91%	2.66	100.00%	1.18
	**CpP1**	98.90%	1.36	100.00%	1.20
	**CpP3**	90.22%	0.00	95.92%	0.00
	**PmP1**	96.81%	0.00	97.96%	0.00
	**AmP1**	97.83%	1.34	100.00%	0.00
	**AmP3**	90.22%	0.00	95.92%	0.00
	**X91**	89.53%	0.00	97.96%	0.00
	**X25**	92.22%	0.00	95.92%	0.00
	**LcP1**	89.13%	0.00	89.80%	1.19
	**LcP3**	92.22%	0.00	93.88%	0.00
	**RtP1**	100.00%	0.66	100.00%	0.00
	**RtP3**	98.90%	1.33	100.00%	1.17
	**LeP1**	96.70%	1.32	100.00%	0.00
	**LeP3**	95.12%	0.74	100.00%	0.00
	**ScP3**	95.45%	0.00	100.00%	0.00
	**EbP5**	91.09%	0.00	97.96%	0.00
	**EbP5^{S4L}**	96.67%	0.00	100.00%	0.00
	**EbP5^{H1H2}**	97.00%	3.70	100.00%	0.00
	**EbP5^{LH2}**	97.83%	0.00	100.00%	0.00
	**EbP5^{S4LH2}**	96.74%	2.00	100.00%	0.00

Supplementary Table 3. Structural assessment of isolated POU domains on *PORE* sequence

**Oct4 homologues/chimeric proteins on *PORE*** (AlphaFold2-based structures)	**Structural assessment using MolProbity on Phenix**	
	Ramachandran favoured (%)	Clash score
**mOct4-*PORE***	99.29	7.51
**MeP1-*PORE***	100.00	8.39
**MeP3-*PORE***	99.29	8.65
**CpP1-*PORE***	99.29	7.71
**CpP3-*PORE***	92.20	4.98
**PmP1-*PORE***	97.20	4.15
**AmP1-*PORE***	98.60	5.21
**AmP3-*PORE***	92.20	5.78
**X91-*PORE***	92.59	7.76
**X25-*PORE***	93.53	5.81
**LcP1-*PORE***	89.36	5.79
**LcP3-*PORE***	92.81	5.81
**RtP1-*PORE***	100.00	5.99
**RtP3-*PORE***	99.29	8.64
**LeP1-*PORE***	97.89	5.20
**LeP3-*PORE***	96.95	6.26
**ScP3-*PORE***	97.06	6.89
**EbP5-*PORE***	93.33	5.07

Supplementary Table 4. Resources used in and generated by this study

Resources	Sources	Catalogue number
**Chemicals for cell culture**		
Glasgow Minimum Essential Medium (GMEM)	Sigma-Aldrich, Merck	G5154-6X500 ML
Dulbecco’s Modified Eagle Medium (DMEM), High Glucose	ThermoFisher	41965-039
Fetal Bovine Serum (FBS)	ThermoFisher	10270-106
KnockOut Serum Replacement (KSR)	ThermoFisher	10828-028
Non-essential amino acids (NEAA)	Sigma-Aldrich, Merck	M7145
L-glutamine	ThermoFisher	25030024
Sodium pyruvate	ThermoFisher	11360039
β –mercaptoethanol	Sigma-Aldrich, Merck	M6250
Leukemia Inhibitory Factor (LIF)	Homemade (Brickman Lab)	-
L-ascorbic acid	Sigma-Aldrich, Merck	A4403-100MG
Alk5 inhibitor	Tocris	A83-01
Gelatin	Homemade (Brickman Lab)	-
Tetracycline hydrochloride (Tc)	Sigma-Aldrich, Merck	T-7660
Puromycin dihydrochloride	Sigma-Aldrich, Merck	P8833
N-2 Supplement	Homemade (Brickman Lab)	-
B-27 Supplement	ThermoFisher	17504001
Neurobasal medium	ThermoFisher	21103049
Dulbecco's Modified Eagle Medium: Nutrient Mixture F-12 (DMEM/F12)	ThermoFisher	21331020
PD0325901	Sigma-Aldrich	PZ0162-5MG
CHIR99021	Axon Med Chem	CT 99021
IWP-2	Calbiochem	681671
Activin A	Peprotech	120-14E
bFGF	Peprotech	450-33
Fibronectin (FN)	Millipore Sigma	FC010-1MG
DAPI	Sigma-Aldrich, Merck	D9542
**Laboratory animals**		
Nanog-eGFP mice	Ian Chamber Laboratory, University of Edinburgh	The mice were maintained, bred, and manipulated at University of Copenhagen, SUND transgenic core facility under the project number 2012-15-2934-00142 and 2013-15-2934-00935. Animal work was authorized by the Danish National Animal Experiments Inspectorate (Dyreforsøgstilsynet, license no. 2018-15-0201-01520) and performed according to national guidelines.
129S2/ScPasCrl mice	Charles Reiver	
**Wild animals**		

Catshark (*Scyliorhinus canicula*)	Purchased from local professional fishermen and maintained by the Oceanological Observatory Aquariology Service of Banyuls sur Mer (France) with aquatic infrastructure.	Experimental work consisted of fixation of euthanised catshark (non-mammalian) embryos prior to hatching and prior to central nervous system differentiation, which does not require ethic agreement according to national regulations (license number A6601601).
**Cell lines**		
Mus musculus (house mouse) Embryonic Stem Cell ZHBTc4 containing tetracycline-suppressible Oct4 transgene	Niwa et al., 2000 (ref.20)	ZHBTc4 ESC cell line was gifted by Hitoshi Niwa (Institute of Molecular Embryology and Genetics, Kumamoto University).
Nanog-GFP mouse embryonic fibroblasts	Homemade	Derived from Nanog-eGFP mouse embryos at embryonic stage 13.5
E14Tg2A ESC	Smith & Hooper, 1987 (ref.21)	Control murine ES cell line
**Commercial Assays**		
Alkaline Phosphatase Staining Kit	Sigma-Aldrich, Merck	86R-1KT
Agilent's microarray kit (SurePrint G3 Mouse GE 8X60K Kit; LowInput QuickAmp Labeling Kit One-Color; RNA Spike In Kit-One Color; Gene Expression Hybridization Kit; Pack 5 Backings 8 arrays per slide; Gene Expression Wash Pack)	Agilent Technologies	G4852A; 5190-2305; 5188-5282; 5188-5242; G2534-60014; 5188-532)
RNeasy™ Mini Kit	Qiagen	74106
SuperScript™ III Reverse Transcriptase	ThermoFisher	18080044
LightCycler® 480 Probes Master Mix	Roche Diagnostics	04902343001
Retro-X™ Concentrator	Clontech	631453
Retro-X™ qRT-PCR Titration Kit	Clontech	631456
**Plasmids**		
pCAGIP-3xflag mOct4 (*Mus musculus* POU5F1)	Livigni et al., 2013 (ref.22)	N/A
pCAGIP-empty	Livigni et al., 2013; Morrison and Brickman 2006 (ref.22-23)	N/A
pCAGIP-3xflag XlPOU91 (*Xenopus laevis* POU91)/XlPOU5F3.1, X91	Livigni et al., 2013 (ref.22)	N/A
pCAGIP-3xflag XlPOU25 (*Xenopus laevis* POU25)/XlPOU5F3.2, X25	Livigni et al., 2013 (ref.22)	N/A
pCAGIP-3xflag LcPOU5F1 (*Latimeria chalumnae* POU5F1), LcP1	This paper	Synthetic gene
pCAGIP-3xflag LcPOU5F3 (*Latimeria chalumnae* POU5F3), LcP3	This paper	Synthetic gene
pCAGIP-3xflag AmPOU5F1 (*Ambystoma mexicanum* POU5F1), AmP1	This paper	cDNA from Elly Tanaka
pCAGIP-3xflag AmPOU5F3 (*Ambystoma mexicanum* POU5F3), AmP3	This paper	cDNA from Elly Tanaka
pCAGIP-3xflag CpPOU5F1 (*Chrysemys picta* POU5F1), CpP1	This paper	Synthetic gene
pCAGIP-3xflag CpPOU5F3 (*Chrysemys picta* POU5F3), CpP3	This paper	Synthetic gene
pCAGIP-3xflag MePOU5F1 (*Macropus eugenii* POU5F1), MeP1	This paper	Synthetic gene

pCAGIP-3xflag MePOU5F3 (*Macropus eugenii* POU5F3), MeP3	This paper	Synthetic gene
pCAGIP-3xflag RtPOU5F1 (*Rhincodon typus* POU5F1), RtP1	This paper	Synthetic gene
pCAGIP-3xflag RtPOU5F3 (*Rhincodon typus* POU5F3), RtP3	This paper	Synthetic gene
pCAGIP-3xflag LePOU5F1 (*Leucoraja erinacea* POU5F1), LeP1	This paper	Synthetic gene
pCAGIP-3xflag LePOU5F3 (*Leucoraja erinacea* POU5F3), LeP3	This paper	Synthetic gene
pCAGIP-3xflag ScPOU5F1 (*Scyliorhinus canicula* POU5F1), ScP1	This paper	Synthetic gene
pCAGIP-3xflag ScPOU5F3 (*Scyliorhinus canicula* POU5F3), ScP3	This paper	Synthetic gene
pCAGIP-3xflag EbPOU5 (*Eptatretus burgeri* POU5), EbP5	This paper	Synthetic gene
pCAGIP-3xflag EbP5LH2 (Hagfish EbP5-coelacanth LcP1 chimeric protein)	This paper	Synthetic gene
pCAGIP-3xflag EbP5S4LH2 (Hagfish EbP5-coelacanth LcP1 chimeric protein)	This paper	Synthetic gene
pCAGIP-3xflag S313 (*Scyliorhinus canicula* POU5F1-POU5F3 chimeric protein)	This paper	Synthetic gene
pCAGIP-N25P91C25 (*Xenopus laevis* POU5F3.2/POU25- POU5F3.1/POU91 chimeric protein)	This paper	Synthetic gene
pCAGIP-N91P25C91 (*Xenopus laevis* POU5F3.2/POU25- POU5F3.1/POU91 chimeric protein)	This paper	Synthetic gene
pMXs-mouse Oct4, mouse Sox2, mouse Klf4, mouse c-Myc	Takahashi and Yamanaka, 2006 (ref.24)	Addgene
pMXs-3xflag Xlpou91 (*Xenopus laevis* POU91)/XlPOU5F3.1, X91	This paper	cDNA from Morrison and Brickman 2006
pMXs-3xflag Xlpou25 (*Xenopus laevis* POU25)/XlPOU5F3.2, X25	This paper	cDNA from Morrison and Brickman 2006
**Deposited Data**		
DNA microarray data of LcPOU5F1, LcPOU5F3 and mOct4-rescued ESCs	This paper	GSE148167; https://www.ncbi.nlm.nih.gov/geo/query/acc.cgi?acc=GSE148167
DNA microarray data of X91 SKM iPSCs, X25 SKM iPSCs and mOct4 SKM iPSCs	This paper	GSE183049; https://www.ncbi.nlm.nih.gov/geo/query/acc.cgi?acc=GSE183049
**Datasets from published sources**		
Resource website for *POU5* genes	NCBI genome database; Ensembl	https://www.ncbi.nlm.nih.gov/genome; https://m.ensembl.org
Resource website for Chondrichthyes *POU5* genes	Marra et al. 2019 (ref.25) Squalomix database (ref.26)	https://transcriptome.riken.jp/squalomix/blast/
Resource website for little skate *POU5* genes	SkateBase (ref.27-28)	http://skatebase.org/
Resource website for thorny skate *POU5* genes	GenomeArk	https://vgp.github.io/genomeark/Amblyraja_radiata/
Resource website for lamprey *POU5* genes	Stowers Institute	https://genomes.stowers.org/organism/Petromyzon/marinus

Resource website for mouse Oct4 structure on *PORE* DNA	PDB: 3L1P<br>(ref.5)	Esch et al., 2013<br>DOI:<br>10.2210/pdb3L1P/pdb
Resource website for mouse Sox2 structure	PDB: 6HT5<br>PDB: 1GT0<br>(ref.8)	Remenyi et al., 2003<br>DOI:<br>10.2210/pdb6HT5/pdb<br>DOI:<br>10.2210/pdb1GT0/pdb
**Software**		
BEAST/BEAUTi/TreeAnnotator (version 1.10.3) and FigTree (version 1.4.2)	Suchard et al., 2018 (ref.17)	https://beast.community
MrBayes (version 3.2.7a)	Huelsenbeck and Ronquist, 2001 (ref.29)	https://nbisweden.github.io/MrBayes/index.html
CellProfiler (version 4.2.1)	Stirling et al., 2021 (ref.30)	https://cellprofiler.org
NIA Array Analysis Tool	Sharov et al., 2005 (ref.31)	https://lgsun.grc.nia.nih.gov/ANOVA/
Morpheus	Morpheus, https://software.broadinstitute.org/morpheus<br>(ref.15)	https://software.broadinstitute.org/morpheus
ShinyGO (version 0.61)<br>(based on Ensembl Release 96, archived on May 23, 2020)	Ge et al., 2019 (ref.32)	http://bioinformatics.sdstate.edu/go/
FCS Express (version 3.0)	DeNovo Software	https://denovosoftware.com
Fiji ImageJ (version 2.3.0)	Schindelin et al., 2012 (ref.33)	https://imagej.net/Fiji
Microsoft Excel (version 16.61.1)	-	-
Prism GraphPad for statistics and data visualization, version 9.1.1 (223)	-	-
AlphaFold2 (version 2.1.0)	Jumper et al., 2021<br>(ref.34)	https://colab.research.google.com/github/deepmind/alphafold/blob/main/notebooks/AlphaFold.ipynb#scrollTo=rowN0bVYLe9n
PyMol (version 2.5.1)	The PyMOL Molecular Graphics System, Version 2.0 Schrödinger, LLC.<br>(ref.35)	https://pymol.org/2/
Phenix (Python-based Hierarchical ENvironment for Integrated Xtallography) (version 1.19.2-4158)	Liebschner et al., 2019 (ref.36)	https://phenix-online.org/download/
UCSF ChimeraX (version 1.2)	Pettersen et al., 2021 (ref.37)	https://www.rbvi.ucsf.edu/chimerax/
BD FACSDiva Software (version 6.1.3)	BD Biosciences	-
ImageLab software (version 6.1)	Bio-Rad	-
LightCycler software (version 1.5)	Roche Molecular Systems	-
PANTHER Classification System (version 16)	Mi et al., 2020 (ref.38)	http://pantherdb.org
CIPRES Science Gateway	Miller et al., 2010 (ref.39)	-

Supplementary Table 5. Antibodies used in this study

Antibodies	Dilution	Sources	Catalogue number
Goat polyclonal anti-mouse Klf4	1:200	R&D Systems	AF3158
Goat polyclonal anti-human Gata6	1:200	R&D Systems	AF1700
Goat polyclonal anti-human/mouse E-cadherin	1:200	R&D Systems	AF748
Rabbit polyclonal anti-mouse/human Oct4	1:1000	Abcam	Ab19857
Mouse monoclonal anti-mouse/rat/human Oct-3/4, clone name: Oct-3/4 (C-10)	1:200	Santa Cruz	Sc-5279
Mouse monoclonal anti-Flag, clone name: M2	1:1000	Sigma-Aldrich, Merck	F3165
Mouse monoclonal anti-mouse Cdx2	1:100	BioGenex	MU392A-UC
Mouse monoclonal anti-mouse p120 catenin, clone name: 98/pp120	1:250	BD Transduction Laboratories, BD Biosciences	610134
Mouse monoclonal anti-mouse Histone H3, clone name: mAbcam 10799	1:2000	Abcam	Ab10799
Rat monoclonal anti-mouse CD31 (PECAM-1) conjugated with APC, clone name: MEC13.3 (RUO)	1:100	BD Pharmingen, BD Biosciences	551262
Mouse c-KIT (CD117) conjugated with APC, clone name: 2B8 (RUO)	1:500	BD Pharmingen, BD Biosciences	561074
Mouse SSEA1 conjugated with Alexa Fluor 647, clone name: MC480 (RUO)	1:50	BD Pharmingen, BD Biosciences	560120
Donkey anti-Goat IgG (H+L) Secondary Antibody-Alexa Fluor 488	1:800	ThermoFisher	A11055
Donkey anti-Goat IgG (H+L) Secondary Antibody-Alexa Fluor 568	1:800	ThermoFisher	A11057
Donkey anti-Goat IgG (H+L) Secondary Antibody-Alexa Fluor 647	1:800	ThermoFisher	A21447
Donkey anti-Mouse IgG (H+L) Secondary Anti-body-Alexa Fluor 488	1:800	ThermoFisher	A21202
Donkey anti-Mouse IgG (H+L) Secondary Anti-body-Alexa Fluor 568	1:800	ThermoFisher	A10037

Supplementary Table 6. Primers used in and generated by this study

Genes	Forward primers	Reverse primers	UPL Probe number
Esrrb (Fig. 3,4)	AACTGGGCCAAGCACATC	ATCTCCATCCAGGCACTCTG	93
Esrrb (Fig. 5,6,8)	CGATTCATGAAATGCCTCAA	ATCTCCATCCAGGCACTCTG	89
Prdm14 (Fig. 3,4)	GGCCATACCAGTGCGTGTA	TGCTGTCTGATGTGTGTTCG	73
Prdm14 (Fig. 6,8)	GGCCATACCAGTGCGTGTA	TGCTGTCTGATGTGTGTTCG	16
Fgf5	GCGAAACTTCAGTCTGTACTTCACT	ACCGGTGAAACCAAAGGTG	95
Gata6	GGTCTCTACAGCAAGATGAATGG	TGGCACAGGACAGTCCAAG	40
Stella	GATGCACAACGATCCAGATTT	TGGAAATTAGAACGTACATACTC CAA	73
IRES-PAC	TGGCTCTCCTCAAGCGTATT	CCCCAGATCAGATCCCATAC	41
Klf4	CGGGAAGGGAGAAGACACT	GAGTTCCTCACGCCAACG	62
Rex1	GCGGTGTGTACTGTGGTGTC	CCCTCAGCTTCTTCTTGCAC	67
Nanog	CCTCCAGCAGATGCAAGAA	GCTTGCACTTCATCCTTTGG	25
Otx2	GGCCTCACTTTGTTCTGACC	AAATCAACTTGCCAGAATCCA	84
Cdh1	ATCCTCGCCCTGCTGATT	ACCACCGTTCTCCTCCGTA	18
Cdh2	GGTGGAGGAGAAGAAGACCAG	GGCATCAGGCTCCACAGTAT	67
Oct6	CATTTTTCGTTTCGTTTTACCC	GAGCGCAGACCCTCTCTG	72
Fgf5	CATCACATTCCCGAATTAAGC	CATCACATTCCCGAATTAAGC	29
Cdx2	CACCATCAGGAGGAAAAGTGA	CTGCGGTTCTGAAACCAAAT	34
Fgf4	GCAAGCTCTTCGGTGTGC	CGTAGGATTCGTAGGCGTTG	29
Gapdh	GGGTTCCTATAAATACGGACT	CCATTTTGTCTACGGGACGA	52
Tbp	GGGGAGCTGTGATGTGAAGT	CCAGGAAATAATTCTGGCTCA	97

Supplementary Table 7. Probe sequences used for whole-mount in situ hybridization

Genes	Sequences of antisense strand
ScPou5f1	ctaactcaagtgcatcgtcctgggcaccggagtggcttgcattggcatgggatggtggtacaagtccccctcgtgaaattg<br>aggtgcgtagatggcggcggtggcgatggctgccccgttgtacccctgcggggcaggcacccccactggggaaggct<br>ggagctggggcatcatgtacccggccagcccctcgtactcttccccaaaattgaaggctgccctcttccccttctgacgcc<br>gattacagaaccagactcgcacgacatccttctccagattcaggtcgtctgcaatctgagtgatctccagggtggagggttt<br>cggacatttcatgaagcaggtctccagcgtccccttcacgttattgtcgatgctcgtgcgtcgcttccgccgtctcgtcggag<br>ccagcatttgctcgatgctgcacagctcctgaaagttatcattgtccttggcttcctccagccagcgctgcaacagaggtttc<br>agtttacacatgttcttgtagctcagctgtagggcttcgaacctgcagatggtcgtctggctgaacatcttgccgtacaaagc<br>cccgagggccaggcccacctctgcctgtgtgaaacccataatgatccgtctcttcttcagttctttagcaaacagctccaaa<br>tcctccgaagtgggcgattc
ScPou5f3	ctaactcgtgtgattgcccatggagacagcagggtgtagagcttgggggaagggagacatgtatataggggggtggcac<br>catagctgcactgtatccttgggttactatggggtcaggtagcatcagagcattgggcgacatgtgaagtggactcccctc<br>gtggatttgtacatcattctcctccatacaaggcaaggtcatcctcttccccttctgcctcctattgcagaaccaaactcggat<br>cacctctttgtctaattggaggtcttcagcgatctgggagatttcttcgctggtgggcttcgggcatttcatgaaataggtctcc<br>aagtttcttttcacaccattctcaatgctagtccgcctcttccgtttcctggactgatccagaacctgttccacgttacaaatctc<br>atgaaggccgccattgttctcggcatcgttcagccagcgctgaagtatcggtttcagtttgcacatgtttttgaagctcagctg<br>cagtgcttcaaacctgcagattgtggtctgactgaacatcttcccataaagattccccaaagctaatccaacgtcagcctgt<br>gtaaatccaagtgttattcgcttgtgtttcagctctttggcaaactgttccattttctctttggtaggatattc

Supplementary References

1. Gold, D. A., Gates, R. D. & Jacobs, D. K. The early expansion and evolutionary dynamics of POU class genes. *Mol. Biol. Evol.* **31**, 3136–3147 (2014).

2. Meredith, R. W. *et al.* Impacts of the Cretaceous Terrestrial Revolution and KPg extinction on mammal diversification. *Science* **334**, 521–524 (2011).

3. Betancur-R, R. *et al.* The tree of life and a new classification of bony fishes. *PLoS Curr.* **5**, ecurrents.tol.53ba26640df0ccaee75bb165c8c26288 (2013).

4. Waterhouse, A. M., Procter, J. B., Martin, D. M. A., Clamp, M. & Barton, G. J. Jalview Version 2—a multiple sequence alignment editor and analysis workbench. *Bioinformatics* **25**, 1189–1191 (2009).

5. Esch, D. *et al.* A unique Oct4 interface is crucial for reprogramming to pluripotency. *Nat. Cell Biol.* **15**, 295–301 (2013).

6. Jin, W. *et al.* Critical POU domain residues confer Oct4 uniqueness in somatic cell reprogramming. *Scientific Reports* **6**, 20818 (2016).

7. Nishimoto, M. *et al.* Oct-3/4 maintains the proliferative embryonic stem cell state via specific binding to a variant octamer sequence in the regulatory region of the UTF1 locus. *Mol Cell Biol* **25**, 5084–5094 (2005).

8. Reményi, A. *et al.* Crystal structure of a POU/HMG/DNA ternary complex suggests differential assembly of Oct4 and Sox2 on two enhancers. *Genes Dev* **17**, 2048–2059 (2003).

9. Jerabek, S. *et al.* Changing POU dimerization preferences converts Oct6 into a pluripotency inducer. *EMBO reports* **18**, 319–333 (2017).

10. Dong *et al.* A balanced Oct4 interactome is crucial for maintaining pluripotency. *Science Advances* **8**, eabe4375 (2022).

11. Li, S. *et al.* Disruption of OCT4 Ubiquitination Increases OCT4 Protein Stability and ASH2L-B-Mediated H3K4 Methylation Promoting Pluripotency Acquisition. *Stem Cell Reports* **11**, 973–987 (2018).

12. Hornbeck, P. V. *et al.* PhosphoSitePlus, 2014: mutations, PTMs and recalibrations. *Nucleic Acids Research* **43**, D512–D520 (2015).

13. Tan, D. S. *et al.* Directed Evolution of an Enhanced POU Reprogramming Factor for Cell Fate Engineering. *Mol Biol Evol* **38**, 2854–2868 (2021).

14. Merino, F. *et al.* Structural basis for the SOX-dependent genomic redistribution of OCT4 in stem cell differentiation. *Structure* **22**, 1274–1286 (2014).

15. Starruß, J. *et al.* Morpheus: a user-friendly modeling environment for multiscale and multicellular systems biology. *Bioinformatics* **30**, 1331-1332 (2014).

16. Simakov, O. *et al.* Deeply conserved synteny resolves early events in vertebrate evolution. *Nat. Ecol. Evol.* **4**, 820–830 (2020).

17. Suchard, M. A. *et al.* Bayesian phylogenetic and phylodynamic data integration using BEAST 1.10. *Virus Evol.* **4**, (2018).

18. Jones, D.T., Taylor, W.R. & Thornton, J.M. The rapid generation of mutation data matrices from protein sequences. *Comput Appl Biosci*. **8**, 275-82 (1992).

19. Godard, B. G. *et al.* Mechanisms of endoderm formation in a cartilaginous fish reveal ancestral and homoplastic traits in jawed vertebrates. *Biol. Open* **3**, 1098–1107 (2014).

20. Niwa, H., Miyazaki, J. & Smith, A. G. Quantitative expression of Oct-3/4 defines differentiation, dedifferentiation or self-renewal of ES cells. *Nat. Genet.* **24**, 372–376 (2000).

21. Smith, A. G. & Hooper, M. L. Buffalo rat liver cells produce a diffusible activity which inhibits the differentiation of murine embryonal carcinoma and embryonic stem cells. *Dev Biol* **121**, 1–9 (1987).

22. Livigni, A. *et al.* A conserved Oct4/POUV-dependent network links adhesion and migration to progenitor maintenance. *Curr. Biol. CB* **23**, 2233–2244 (2013).

23. Morrison, G. M. & Brickman, J. M. Conserved roles for Oct4 homologues in maintaining multipotency during early vertebrate development. *Development* **133**, 2011–2022 (2006).

24. Takahashi, K., & Yamanaka, S. Induction of pluripotent stem cells from mouse embryonic and adult fibroblast cultures by defined factors. *Cell* **126**, 663–676 (2006)

25. Marra, N. J. *et al.* White shark genome reveals ancient elasmobranch adaptations associated with wound healing and the maintenance of genome stability. *Proc. Natl. Acad. Sci.* **116**, 4446 (2019).

26. Hara, Y. *et al.* Shark genomes provide insights into elasmobranch evolution and the origin of vertebrates. *Nat. Ecol. Evol.* **2**, 1761–1771 (2018).

27. Wang, Q. *et al.* Community annotation and bioinformatics workforce development in concert-Little Skate Genome Annotation Workshops and Jamborees. *Database J. Biol. Databases Curation* 2012, bar064–bar064 (2012).

28. Wyffels, J. *et al.* SkateBase, an elasmobranch genome project and collection of molecular resources for chondrichthyan fishes. *F1000Research* **3**, 191 (2014).

29. Huelsenbeck, J. P. & Ronquist, F. MRBAYES: Bayesian inference of phylogenetic trees. *Bioinformatics* **17**, 754–755 (2001).

30. Stirling, D. R., Carpenter, A. E. & Cimini, B. A. CellProfiler Analyst 3.0: accessible data exploration and machine learning for image analysis. *Bioinformatics* **37**, 3992–3994 (2021).

31. Sharov, A. A., Dudekula, D. B. & Ko, M. S. H. A web-based tool for principal component and significance analysis of microarray data. *Bioinformatics* **21**, 2548–2549 (2005).

32. Ge, S. X., Jung, D. & Yao, R. ShinyGO: a graphical gene-set enrichment tool for animals and plants. *Bioinformatics* (2019)

33. Schindelin, J. *et al.* Fiji: an open-source platform for biological-image analysis. *Nat. Methods* **9**, 676–682 (2012).

34. Jumper, J. *et al.* Highly accurate protein structure prediction with AlphaFold. *Nature* **596**, 583–589 (2021).

35. The PyMOL Molecular Graphics System, Version 2.0 Schrödinger, LLC.

36. Liebschner, D. *et al.* Macromolecular structure determination using X-rays, neutrons and electrons: recent developments in Phenix. *Acta Crystallogr D Struct Biol* **75**, 861–877 (2019).
37. Pettersen, E. F. *et al.* UCSF ChimeraX: Structure visualization for researchers, educators, and developers. *Protein Sci* **30**, 70–82 (2021).
38. Thomas, P. D. *et al*. PANTHER: a library of protein families and subfamilies indexed by function. *Genome Res* **13**, 2129–2141 (2003).
39. M. A. Miller, W. Pfeiffer, & T. Schwartz. Creating the CIPRES Science Gateway for inference of large phylogenetic trees. in *2010 Gateway Computing Environments Workshop (GCE)* 1–8 (2010).
