# Supplementary material for: Evolutionary origin of vertebrate OCT4/POU5 functions in supporting pluripotency
Source: Nat Commun. 2022 Sep 21;13:5537. doi: 10.1038/s41467-022-32481-z (PMC9492771; doi:10.1038/s41467-022-32481-z)
Supplement: Supplementary file 2 — Description of Additional Supplementary Files [file 41467_2022_32481_MOESM2_ESM.pdf]

Description of Additional Supplementary Files

Supplementary Data 1, related to Fig. 1. Sequences of the POU specific domain (POU_S), Linker domain and POU homeodomain (POU_{HD}) of POU5 proteins used to generate the consensus sequence of POU5 protein (Fig. 1a) and the phylogenetic tree showing the evolutionary rates of vertebrate POU5 proteins (Fig. 1c)

Supplementary Data 2, related to Fig. 1. Accession numbers of the sequences used in synteny and phylogenetic analyses and predictions of *POU5* coding sequences from genomic databases

Supplementary Data 3, related to Fig. 5. Gene Expression Data and Gene Ontology Analysis based on the transcriptomic comparisons of coelacanth (Lc) POU5-rescued and mOct4-rescued ESC lines
