# Supplementary material for: Evolutionary origin of vertebrate OCT4/POU5 functions in supporting pluripotency
Source: Nat Commun. 2022 Sep 21;13:5537. doi: 10.1038/s41467-022-32481-z (PMC9492771; doi:10.1038/s41467-022-32481-z)
Supplement: Supplementary file 3 — Supplementary Data 1 [file 41467_2022_32481_MOESM3_ESM.pdf]

>HsPOU5F1
QDIKALQKELEQFAKLLKQKRITLGYTQADVGLTLGVLFGKVFSQTTICRFEALQLSFKNMCKLRPLLQKWVEEADNNENLQEICKA--------------------------------
---ETLV-QARKRK-RTSIENRVRGNLENLFLQCPKPTLQQISHIAQQLGLEKDVVRVWFCNRRQKGKRSS
>SsPOU5F1
QDIKALQKDLEQFAKLLKQKRITLGYTQADVGLTLGVLFGKVFSQTTICRFEALQLSFKNMCKLRPLLQKWVEEADNNENLQEICKA--------------------------------
---ETLV-QARKRK-RTSIENRVRGNLESMFLQCPKPTLQQISHIAQQLGLEKDVVRVWFCNRRQKGKRSS
>LaPOU5F1
QDIKTRQKDLEQFAKLLKQKRITLGYTQADVGLTLGVLFGKVFSQTTICRFEALQLSFKNMCKLRPLLQKWVEEADNNENLQEICKT--------------------------------
---ENLLQQARKRK-RTSIENRVRGSLENLFLQCPKPSLQQIGHIAQQLGLEKDVVRVWFCNRRQKGKRSS
>MePOU5F1
PQETPSPEELEQFAKELKRKRITLGYTQADVGITLGALFGKVFSQTTICRFEAQQLSFKNMCKLRPLLQKWLEAADDNDHLQELCKA--------------------------------
---ETVLQQARKRK-RTSIENGVRGNLETMFLQCPKPTLQQISNIAEELGLEKDVVRVWFCNRRQKGKRSN
>OaPOU5F1
QQETPSREELEQFAKELKRKRITLGYTQADVGVTLGALFGKVFSQTTICRFEAQQLSFKNMCKLRPLLQRWLEAADDNDRLQEMCNA--------------------------------
---ETVLQQARKRK-RTSIENKVRGNLETMFLQCPKPNLQQISSIAEELGLEKDVVRVWFCNRRQKGKRGS
>AlsPOU5F1
ENETMTTEEMEQFVRELKHKRIMLGFTQADVGLALGVLYGRMFSQTTICRFEAQQLSFRNMCRLRPLLHRWLREADARPELQQLCGM--------------------------------
---E--VTQAHKRK-RTRIESGARWRLEICFRHCPKPGLPQIARIARSLGLDKDVVRVWFCNRRQKGKRRG
>CpPOU5F1
GEETPSTEEMEQFAKELKHKRITLGFTQADVGLALGVLYGKMFSQTTICRFEALQLSFKNMCKLKPLLQRWLDEADGNANLQEMCSM--------------------------------
---ESALLQARKRK-RTSIETAARGSLESYFLRCPKPSLQEIAHIAHDLHLDKDVVRVWFCNRRQKGKRSG
>AcPOU5F1
DSQEDASAKMEQFAKELKHKRITMGFTQADVGLSLGLLYGKMFSQTTICRFEALQLSFKNMSKLKPLLQRWLQEADRNENLQELCTM--------------------------------
---ESAMIQARKRK-RTSIENTVRGALEAYFRRCSKPSLQQITQIASELGLDKDVVRVWFCNRRQKGKRNI
>GjPOU5F1
DEEIPTSEEMEQFAKDLKHKRITLGFTQADVGLALGVLYGKMFSQTTICRFEALQLSFKNMCKLKPLLQRWLHEADSNENLQQLCSS--------------------------------
---ESAMIQARKRK-RTSIETTVRGSLETYFLRCPKPTLQEISQIANDLHLEKDVVRVWFCNRRQKGKRNL
>AmPOU5F1
DEEGGTSADLEQFAKELKQKRITLGFTQADVGLALGALYGKMFSQTTICRFEALQLSFKNMCKLRPLLQRWLVEADTNENLQELCNL--------------------------------
---ENALQQARKRK-RTSIENSVKDNLEAFFLKCPKPTHQEIAHISEDLNLEKDVVRVWFCNRRQKGKRSI
>LcPOU5F1
TEDTPTTDDLEQFAKELKHKRISLGFTQADVGLALGALYGKMFSQTTICRFEALQLSFKNMCKLKPLLQRWLDEADTNENLQELCNL--------------------------------
---EQVLSQARKRK-RTSLETTAKGTLESFFLKCSKPSLQEIAQIAEELSLDKDVVRVWFCNRRQKGKRSL
>LePOU5F1
NEXXXXXXXXXXXXXXXXXXXXXXXXXXXXXXXXXXXXXGKMFSQTTICRFEALQLSYKNMCKLKPLLQRWLEEAKDSDNFQELCSI--------------------------------
---EQTLASSRKRKRRTSIDNNVKEGLENFFTVCPKPSTQEITKIADDLNLEKDVVRVWFCNRRQKGKRAA
>ArPOU5F1
NEDSPTSEDLEQFAKELKMKRITMGFTQAEVGLALGALYGKMFSQTTICRFEALQLSYKNMCKLKPLLQRWLEEAKDSDNFQELCSI--------------------------------
---EQTLASSRKRKRRTSIDXNVKEALENFFTVXPKPSTQEITKIADDLNLEKDVVRVWFCNRRQKGKRAA
>CpuPOU5F1
AEETPTSEDLEQFAKELKKNRIILGFTQAEVGLALGALYGKMFSQTTICRFEALQLSYKNMCKLKPLLQRWLEEANDTENFQELCSI--------------------------------
---EQRLAPSRKRKQRTSIDNNLREALESAFLKCPKPSAQELGQIADNLILEKDVVRVWFCNRRQKGKRAL
>RtPOU5F1
GEETPTSEDLKHFAKKLKKKRIIMGFTQAEVGLALGALYGKMFSQTTICRFEALQLSYKNMCKLRPLLQRWLEEAKDNENFQELCSM--------------------------------
---EQRLATARKRKRRTSIDSNVKGLLEAAFIKCPKPSAQEIIQIADDLNLEKDVVRVWFCNRRQKGKRAL

>CcPOU5F1
DEETLTSEDLEQFAKELKRKRIIMGFTQAEVGLALGTLYGKMFSQTTICRFEALQLSYKNMCKLKPLLQRWLEAAKNNENLQELCSM--------------------------------
---EQTQAPTRRRKQRTSIDNNVKGSLEAAFMKCPKPSTLEITQIADDLDLEKDVVRVWFCNRRQKGKRAA
>ScPOU5F1
DEESPTSEDLELFAKELKKRRIIMGFTQAEVGLALGALYGKMFSQTTICRFEALQLSYKNMCKLKPLLQRWLEEAKDNDNFQELCSI--------------------------------
---EQMLAPTRRRKRRTSIDNNVKGTLETCFMKCPKPSTLEITQIADDLNLEKDVVRVWFCNRRQKGKRAA
>StPOU5F1
DEESPTSEDLEQFAKELKKRRIILGFTQAEVGLALGALYGKMFSQTTICRFEALQLSYKNMCKLKPLLQRWLEEAKDNENFQELCSI--------------------------------
---EQRLAPMRRRKRRTSIDNNVKGTLETFFVKCPKPSTLEIAQIADNLNLEKDVVRVWFCNRRQKGKRAA
>OaPOU5F3
EEEXXXXXXXXXFAQELRHKRITLGLTQAEVGMALGTLYGRVFSQTTICRFEALQLSFKNMCKLKPILHRWLNKAESADHPQETYIG--------------------------------
---ERVLVPARKRKRRTSIQSSIKVSLESLFCRCGKPSPQQICDIAQDLQLDKDXXXXXXXXXXXXXXXXX
>MePOU5F3
EEDTPTSEELEKFAKELKHKRISLGFTQADVGMALGTLYGKMFSQTTICRFEALQLSFKNMCKLKPLLQRWLQAVENTDNPQEMCSM--------------------------------
---EQVLAQARKRKRRTSIETSVKGTLEGFFRRCGKPTPQQICDLAEELHLDKDVVRVWFCNRRQKGKRLL
>GgPOU5F3
DEDAPTSEELEQFAKDLKHKRIMLGFTQADVGLALGTLYGKMFSQTTICRFEALQLSFKNMCKLKPLLQRWLNEAENTDNMQEMCNA--------------------------------
---EQVLAQARKRKRRTSIETNVKGTLESFFRKCVKPSPQEISQIAEDLNLDKDVVRVWFCNRRQKGKRLL
>AlsPOU5F3
DEDTPTSEELEQFAKDLKHKRITLGFTQADVGLALGTLYGKMFSQTTICRFEALQLSFKNMCKLKPLLQRWLNEAENNDNMQELCNA--------------------------------
---EQVLAQARKRKRRTSIETNVKGTLESFFRKCVKPSPQEISQIAEDLNLDKDVVRVWFCNRRQKGKRLL
>CpPOU5F3
EDATPTSEELEQFAKDLKHKRITLGFTQADVGMALGTLYGKMFSQTTICRFEALQLSFKNMCKLKPLLQRWLNEVENSDSLQELCNA--------------------------------
---EQVLAQARKRKRRTSIENNVKGTLESFFRKCIKPSPQQISQIAEDLNLDKDVVRVWFCNRRQKGKRLL
>SpPOU5F3
DEDTPTSEELEQFSKDLKHKRITLGFTQADVGLALGTLYGKMFSQTTICRFEALQLSFKNMCKLKPLLQRWLNEAENNENLQELCNA--------------------------------
---EQVLAQARKRKRRTSIETNVKGTLENVFRKCVKPSPQEISQIAEDLNLDKDVVRVWFCNRRQKGKRLL
>XlOCT91
EEEAPNSGEMEQFAKDLKHKRITMGYTQADVGYALGVLFGKTFSQTTICRFESLQLSFKNMCKLKPLLRSWLHEVENNENLQEIISR--------------------------------
---GQIIPQVQKRKHRTSIENNVRCTLENYFMRCSKPSAQEIAQIARELNMEKDVVRVWFCNRRQKGKRQV
>XlOCT25
EEEVPSESEMEQFAKDLKHKRVSLGYTQADVGYALGVLYGKMFSQTTICRFESLQLSFKNMCQLKPFLERWVVEAENNDNLQELINR--------------------------------
---EQVIAQTRKRKRRTNIENIVKGTLESYFMKCPKPGAQEMVQIAKELNMDKDVVRVWFCNRRQKGKRQG
>XlOCT60
EEDGMTLEEMEEFAKELKQKRVALGYTQGDIGHALGILYGKMFSQTTICRFESLQLTFKNMCKLKPLLEQWLGEAENNDNLQEMIHK--------------------------------
---AQIEEQNRKRKMRTCFDTVLKGQLEGHFMCNQKPGARELTEIAKELSLEKDVVRVWFCNRRQKEKSKF
>AmPOU5F3
DEDTPTNEELEQFAKALKHKRITLGFTQADVGLALGSLYGRMFSQTTICRFEALQLSFKNMCKLKPLLQRWLNEAENTDNMEELCNM--------------------------------
---EQMLAQARKRKRRTSIENNVRGTLESFFLKCSKPGPQEISQIAEDLSLDKDVVRVWFCNRRQKGKRLL
>LcPOU5F3
DEDNPTTEELEQFAKELKHKRITLGFTQADVGLALGTLYGKMFSQTTICRFEALQLSFKNMCKLKPLLQRWLNEAENNENLQEMCNI--------------------------------
---EQVLAQARKRKRRTSIENNVKGTLENYFLKCPKPTSQEISQIADDLNLEKDVVRVWFCNRRQKGKRLA
>AsPOU5F3
EEENLSTEELEQFAKELKHKRITLGFTQADVGLALGNLYGKMFSQTTICRFEALQLSFKNMCKLKPLLQRWLNEAENTDNPQDMYKI--------------------------------
---ERVFADSRKRKRRTSLEVTVRGALESYFIKCPKPNTQDITQIAEDLRLEKDVVRVWFCNRRQKGKRLA

```
>LoPOU5F3
DEEVITSQDLEQFSKEFKQKRITMGFTQADVGLALGHLYGKMFSQTTICRFEALQLSYKNLCKLKPLLQSWLAEAEASENPQDLFKV--------------------------------
---ERVFLDTRKRKRRTSLETSVRGALESYFAGCPKPNAQEMTRIADDLGLERDVVRVWFCNRRQKGKRLL
>DrPOU5F3
EEETLTTEDLEQFAKELKHKRITLGFTQADVGLALGNLYGKMFSQTTICRFEALQLSFKNMCKLKPLLQRWLNEAENSENPQDMYKI--------------------------------
---ERVFVDTRKRKRRTSLEGTVRSALESYFVKCPKPNTLEITHISDDLGLERDVVRVWFCNRRQKGKRLA
>OlPOU5F3
EEENLSTEELEQFAKELKHKRITLGFTQADVGLALGNLYGKMFSQTTICRFEALQLSFKNMCKLKPLLQRWLDEAETSENPQDMYKI--------------------------------
---ERVFADTRKRKRRTSLEGAVRSALEAYFIKCPKPNTQEITHISDDLGLERDVVRVWFCNRRQKGKRLA
>TrPOU5F3
EEEILTTEELEQFAKELKHKRITLGFTQADVGLALGNLYGKMFSQTTICRFEALQLSFKNMCRLKPLLQKWLNEAETTENPQDMYKV--------------------------------
---ERVFVDTRKRKRRTSLEGAVRSALEAYFIKCPKPNTQEITHISDDLGLERDVVRVWFCNRRQKGKRLA
>CmPOU5F3
EEEYRTKGKMEEFAKELKRKRITLGFTQADVGIALGNLYGKMFSQTTICRFEALQLSFKNMCKLKPILERWLNDAQNHDGVHEICVT--------------------------------
---EQVTDQSRKRKRRTSIENSVKGNLETCFMKCPRPTSEEITQIAEDLNLEKDVIRVWFSNRRQKGKRMT
>CpuPOU5F3
SEEYPTKEKMEQFAKELKHKRITLGFTQADVGLALGNLYGKMFSQTTICRFEALQLSFKNMCKLKPILQRWLNDAENNGGLQEICNM--------------------------------
---EQVLDQSRKRKRRTSIENGVKRNLETYFMKCPKPTSEEISQIAEDLCLDKEVIRVWFCNRRQKGKRMT
>RhPOU5F3
EEEYPTKEKMEQFAKELKHKRITLGFTQADVGLALGNLYGKMFSQTTICRFEALQLSFKNMCKLKPILQRWLNDAENNGSLHEICNV--------------------------------
---EQVLDQSRKRKRRTSIENGVKRNLETYFMKCPKPTSEEISQIAEDLCLDKEVIRVWFCNRRQKGKRMT
>HzPOU5F3
EEEYPTKEKMEQFAKELKHKRITLGFTQADVGLALGNLYGKMFSQTTICRFEALQLSFKNMCKLKPILQRWLNDAENNGGLHEICNV--------------------------------
---EQVLDQSRKRKRRTSIENGVKRNLETYFMKCPKPTSEEISQIAEDLRLDKEVVRVWFCNRRQKGKRMT
>CcPOU5F3
EEEYPTKEKMEQFAKELKHKRITLGFTQADVGLALGNLYGKMFSQTTICRFEALQLSFKNMCKLKPILQRWLNDAENNGSLHEICNM--------------------------------
---EQVLDQSRKRKRRTSIENGVKRNLETYFMKCPKPTSEEISQIADDLRLDKEVIRVWFCNRRQKGKRMT
>StPOU5F3
EEEYPTKEKMEQFAKELKHKRITLGFTQADVGLALGNLYGKMFSQTTICRFEALQLSFKNMCKLKPILQRWLNDAENNGGLQEICHV--------------------------------
---EQVLDQSRKRKRRTSIENGVKRNLETYFMKCPKPTSEEISQIAEDLQLDKEVIRVWFCNRRQKGKRMT
>ScPOU5F3
EEEYPTKEKMEQFAKELKHKRITLGFTQADVGLALGNLYGKMFSQTTICRFEALQLSFKNMCKLKPILQRWLNDAENNGGLHEICNV--------------------------------
---EQVLDQSRKRKRRTSIENGVKRNLETYFMKCPKPTSEEISQIAEDLQLDKEVIRVWFCNRRQKGKRMT
>LePOU5F3
EEEYPTKEKMEQFAKELKHKRITLGFTQADVGLALGNLYGKMFSQTTICRFEALQLSFKNMCKLKPILQRWLNDAESDGGIHEICNV--------------------------------
---EQVLDQSRKRKRRTSIENGVKNNLETYFMKCPKPTSEEISQIAEDLRLDKEVIRVWFCNRRQKGKRMT
>ArPOU5F3
EEEYPTKEKMEQFAKELKHKRITLGFTQADVGLALGNLYGKMFSQTTICRFEALQLSFKNMCKLKPILQRWLNDAESDGGIHEICNV--------------------------------
---EQVLDQSRXRKRRTSIENGVKNXLETYFMKSPKPTSEEISQIAEDLRLDKEVIRVWFCNRRQKGKRMT
>OkPOU5F3
XXXYPTKEKMEQFAKELKHKRITLGFTQADVGLALGNLYGKMFSQTTICRFEALQLSFKNMCKLKPILQRWLNDAESDGGIHEICNV--------------------------------
---EQVLDQSRKRKRRTSIENGVKSNLETYFMKCPKPTSEEISQIAEDLRLDKEVIRVWFCNRRQKGKRMT
>PmPOU5
AASDGNIDELARFAKELKVKRVNLGFTQAEMGLSIGSLCGRVFSQTTICRFEALQLSQRNLCKLQPLFKLWMDDVGVNGGSGGVSSVGGGVGGK-------------------------
---TPREVGYRMRKKRTYFASYTRMQLEAYYNVCSKPNMSAIASIAQQLKLENSVVRLWFSNRRQKCRKAS
```

```
>LjPOU5
VGSDGNIDELARFAKELKMKRVNLGFTQAEMGISIGSLCGRVFSQTTICRFEALQLSQRNLCKLQPLFKLWMEDVQSNGSISSNGGNGGNGGNGGNGGNGGNGGNDTKENAAAGQT
LSKTPREVVQRIRKKRTYFASYTRMQLEAYYNVCSKPNMSAIASIAQHLKLENNVVRLWFSNRRQKCKKAC
>EbPOU5
DEGTISSETLAQFARDLKHKRITLGFTQADVGVALGSLYGRIFSQTTICRFEALQLSYRNMCKLQPLLERWMIEAENADNVKEVCHLFLFNSSMD---------------------
---QSLANVNKLRRRRTTIENGVRDTLEAWYLVCSKPSAKEIARIAGELNLDKEVVRVWFCNRRQKLKQLS
>BfPOU2
GDEPSQLEELEQFAKMFKQRRIKLGFTQGDVGLAMGKLYGNDFSQTTISRFEALNLSFKNMCKLKPLLEKWLQDADSSMANPGALGSPHGSI------------------------
---E-VLG--RRRKKRTSIETNVRVALEKAFIQNPKPTSEEIGIIAEQLGMEKEVVRVWFCNRRQKEKRIN
>BfPOU3.1
XEESPTADELEQFAMVFKQRRIKLGFTQADVGLALGTIHGNVLSQTTICRFEALQLSFKNMCKLKPLLQKWLETADSNMSPGSLPKDEGLGG------------------------
---E-VITPGRKRKKRTSIEVSVKGALETFFYKQPKPSAIEISQLSEGLNLDKEVVRVWFCNRRQKERRMS
>BfPOU3.2
DDDTPTSDDLEQFAKQFKQRRIKLGFTQADVGLALGTLYGNVFSQTTICRFEALQLSFKNMCKLKPLLQKWLEEADSSSGSPSSIDK-----------------------------
-----IAAQGRKRKKRTSIEVTVKGALESHFLKQPKPSAQEIAQLADSLQLEKEVVRVWFCNRRQKEKRMT
```
