# Supplementary material for: Evolutionary origin of vertebrate OCT4/POU5 functions in supporting pluripotency
Source: Nat Commun. 2022 Sep 21;13:5537. doi: 10.1038/s41467-022-32481-z (PMC9492771; doi:10.1038/s41467-022-32481-z)
Supplement: Supplementary file 6 — Reporting Summary [file 41467_2022_32481_MOESM6_ESM.pdf]

nature research

Corresponding author(s): Joshua M Brickman
Sylvie Mazan

Last updated by author(s): Jul 22, 2022

Reporting Summary

Nature Research wishes to improve the reproducibility of the work that we publish. This form provides structure for consistency and transparency in reporting. For further information on Nature Research policies, see Authors & Referees and the Editorial Policy Checklist .

Statistics

For all statistical analyses, confirm that the following items are present in the figure legend, table legend, main text, or Methods section.

n/a	Confirmed	
☐	☒	The exact sample size (*n*) for each experimental group/condition, given as a discrete number and unit of measurement
☐	☒	A statement on whether measurements were taken from distinct samples or whether the same sample was measured repeatedly
☐	☒	The statistical test(s) used AND whether they are one- or two-sided<br>*Only common tests should be described solely by name; describe more complex techniques in the Methods section.*
☒	☐	A description of all covariates tested
☒	☐	A description of any assumptions or corrections, such as tests of normality and adjustment for multiple comparisons
☐	☒	A full description of the statistical parameters including central tendency (e.g. means) or other basic estimates (e.g. regression coefficient) AND variation (e.g. standard deviation) or associated estimates of uncertainty (e.g. confidence intervals)
☐	☒	For null hypothesis testing, the test statistic (e.g. *F*, *t*, *r*) with confidence intervals, effect sizes, degrees of freedom and *P* value noted<br>*Give P values as exact values whenever suitable.*
☐	☒	For Bayesian analysis, information on the choice of priors and Markov chain Monte Carlo settings
☒	☐	For hierarchical and complex designs, identification of the appropriate level for tests and full reporting of outcomes
☒	☐	Estimates of effect sizes (e.g. Cohen's *d*, Pearson's *r*), indicating how they were calculated

Our web collection on statistics for biologists contains articles on many of the points above.

Software and code

Policy information about availability of computer code

Data collection

1.Alkaline phosphatase staining images were obtained using Leica-5500B microscope.
2.Immunofluorescence images were obtained using Leica AF6000 widefield and Leica SP8 Confocal microscope.
3.qRT-PCR data were obtained using LightCycler 480 II (Roche) and the concentration of transcripts of each gene was calculated in LightCycler 480 software (version 1.5.162 SP3) based on the cDNA pool-derived standard curve.
4.Global transcriptome of POU5-rescued lines were obtained by using Agilent one-color microarray-based gene expression analysis. The processed 8X60K Agilent slides containing probes specific for mouse genes (Grid_GenomicBuild: mm9:NCBI37:Jul2007) were scanned with Agilent Scanner. Agilent Feature Extraction (FE) version: 11.0.1.1. The raw data was extracted from the scanner and "gProcessedSignal" values and "ProbeName" were recorded.
5. Western blot stainings were visualized using ChemiDoc MP (Bio-Rad) and ImageLab software (version 6.1.0 build 7).
6. AlphaFold structures (https://colab.research.google.com/github/deepmind/alphafold/blob/main/notebooks/AlphaFold.ipynb#scrollTo=rowN0bVYLe9n), Jumper et al., 2021 (version 2.1.0)
7. Flow cytometry data was acquired using BD LSR Fortessa with BD FACSDiva Software (version 6.1.3).

Data analysis

1.BEAST/BEAUTi/TreeAnnotator (version 1.10.3) and FigTree (version 1.4.2), https://beast.community, Suchard et al., 2018
2.MrBayes, version 3.2.7a, https://nbisweden.github.io/MrBayes/index.html, Huelsenbeck and Ronquist, 2001
3.CellProfiler, version 4.2.1, https://cellprofiler.org, Stirling et al., 2021
4.NIA Array Analysis Tool, https://lgsun.grc.nia.nih.gov/ANOVA/, Sharov et al., 2005
5.Morpheus, https://software.broadinstitute.org/morpheus
6.PANTHER Classification System, version 16, http://pantherdb.org, Mi et al., 2020
7.ShinyGO, version 0.61, http://bioinformatics.sdstate.edu/go, Ge et al., 2019
8.FCS Express, version 3.0, https://denovosoftware.com, DeNovo Software
9.Fiji ImageJ, version 2.3.0/1.53f, https://imagej.net/Fiji, Schindelin et al., 2012
10.Excel for qRT-PCR analysis, version 16.61.1
11.Prism GraphPad for statistics and data visualization, version 9.1.1 (223)

12.Phenix, version 1.19.2-4158, https://phenix-online.org/download/, Liebschner et al., 2019, for structural validation
13.PyMol (The PyMOL Molecular Graphics System, Schrödinger, LLC.) for structural visualization and analysis, version 2.5.1, https://pymol.org/2/
14.UCSF ChimeraX, version 1.2 (2021-04-27), https://www.rbvi.ucsf.edu/chimerax/, Pettersen et al., 2021, for structural visualization and analysis

For manuscripts utilizing custom algorithms or software that are central to the research but not yet described in published literature, software must be made available to editors/reviewers. We strongly encourage code deposition in a community repository (e.g. GitHub). See the Nature Research guidelines for submitting code & software for further information.

Data

Policy information about availability of data

All manuscripts must include a data availability statement. This statement should provide the following information, where applicable:
- Accession codes, unique identifiers, or web links for publicly available datasets
- A list of figures that have associated raw data
- A description of any restrictions on data availability

Source data for POU5 and other syntenic genes for phylogenetic analysis and evolutionary rate analysis are provided in Supplementary Data 1-2. Other published resources including Squalomix database ((https://transcriptome.riken.jp/squalomix/blast/), SkateBase (http://skatebase.org/), GenomeArk (https://vgp.github.io/genomeark/Amblyraja_radiata/), Stowers Institute (https://genomes.stowers.org/organism/Petromyzon/marinus) were used to obtain Chondrichthyes and cyclostome POU5 gene sequences. Global transcriptome data found in Supplementary Data 3 have been deposited in GSE148167 (DNA microarray data of LcPOU5F1, LcPOU5F3 and mOct4-rescued ESCs, https://www.ncbi.nlm.nih.gov/geo/query/acc.cgi?acc=GSE148167) and GSE183049 (DNA microarray data of X91 SKM iPSCs, X25 SKM iPSCs and mOct4 SKM iPSCs, https://www.ncbi.nlm.nih.gov/geo/query/acc.cgi?acc=GSE183049). AlphaFold2-generated structural models of POU5 proteins are available from the corresponding author upon request. The authors declare that all other data supporting the findings of this study are available within the paper and its supplementary information files.

Field-specific reporting

Please select the one below that is the best fit for your research. If you are not sure, read the appropriate sections before making your selection.

☒ Life sciences ☐ Behavioural & social sciences ☐ Ecological, evolutionary & environmental sciences

For a reference copy of the document with all sections, see nature.com/documents/nr-reporting-summary-flat.pdf

Life sciences study design

All studies must disclose on these points even when the disclosure is negative.

Sample size	1.The sample size of species examined in this study was sufficient based on these criteria: (1) species carrying both POU5 forms and (2) availability of full-length cDNA sequences of POU5 paralogues.<br><br>2.For POU5-rescued ESC assay, rescue indices were calculated from at least three independent culture plates in both tetracycline+puromycin (-Oct4) and only puromycin (+Oct4) conditions. The sample size of independent experiment (n=3) was sufficient based on 1) the reproducibility of results (alkaline phosphatase staining) of the positive and negative controls (mOct4 and empty vector) and some comparisons of recent results (X91 and X25) to our previous data (Livigni et al., 2013; Brickman and Morrison 2006) and 2) low variance of positive control (mOct4, closely to 1) and negative control (empty vector, always 0) and 3) all independent experiments providing consistency of results.<br><br>3.For qRT-PCR and Western blot analysis of POU5-rescued ESCs, at least three biological clones were compared and visualized with bar charts showing mean and error bars indicate standard deviation of values from three biologically independent clones. With positive control (E14 Tg2A ESCs and ZHBTc4) and negative control (ZHBTc4+Tetracycline), three biological independent data were sufficient to detect differences between positive and negative control data.<br><br>4.For iPSC generation, experiments with mouse Oct4 condition (as a positive control) could be replicated at least three times with similar results. The reprogramming efficiency was based on the number of Nanog-eGFP expressing colonies obtained from independent infections was consistent across three experiments. Based on this, we concluded that three biologically independent iPSC inductions were sufficient to distinguish the differences in reprogramming capacity from different POU5 homologues. The induction was performed from different infections using the same batch of virus production.
Data exclusions	Rescue analysis of an initial Lamprey POU5 was performed but later excluded due to unavailability of complete full-length sequence.
Replication	1.All POU5-rescued ESC experimental data was replicated in at least in three independent experiments. At least three clones (three biological replicates) from a POU5-rescue ESC experiment were used for qRT-PCR and Western blot analysis. Results from frog and mouse POU5-rescued ESCs in this study reproduced data previously reported by our lab (Morrison and Brickman 2006; Livigni et al., 2013). We can confirm that replications of the rescue experiments were successful.<br><br>2.For iPSC generation, at least three independent iPSC inductions (different infection from the same batch of virus production) were performed. We can confirm replications of iPSC generation were successful based on our homemade retrovirus production. At least four iPSC clonal lines from different independent iPSC induction were analysed.

Randomization	Randomization:<br>1.Rescued ESC Colony picking: At least 10 colonies from each POUV-rescued ESC assay were randomly picked and expanded.<br>2. Healthy expanding ESC lines were chosen at random from clonal expansion for further analysis. Given the nature of stem cell biology, only expandable ESC clones can be used for further analysis including qRT-PCR, western blot, immunofluorescence and DNA microarray.<br>2.iPSC colony picking: Colonies were picked at random and expanded. Given the nature of stem cell biology, only Nanog-eGFP positive iPSC colonies were chosen for further analysis. Nanog-eGFP negative colonies have been excluded due to partially reprogrammed state and incapacity to expand.
Blinding	Given the nature of developmental and stem cell biology and the collection of material, blinding is not relevant.

Reporting for specific materials, systems and methods

We require information from authors about some types of materials, experimental systems and methods used in many studies. Here, indicate whether each material, system or method listed is relevant to your study. If you are not sure if a list item applies to your research, read the appropriate section before selecting a response.

Materials & experimental systems

n/a	Involved in the study
☐	☒ Antibodies
☐	☒ Eukaryotic cell lines
☒	☐ Palaeontology
☐	☒ Animals and other organisms
☒	☐ Human research participants
☒	☐ Clinical data

Methods

n/a	Involved in the study
☒	☐ ChIP-seq
☐	☒ Flow cytometry
☒	☐ MRI-based neuroimaging

Antibodies

Antibodies used	For immunofluorescence and flow cytometry<br>primary antibodies including<br>1.Goat polyclonal anti-mouse Klf4 (R&D Systems, AF3158), Dilution 1:200<br>2.Goat polyclonal anti-human Gata6 (R&D Systems, AF1700), Dilution 1:200<br>3.Goat polyclonal anti-human/mouse E-cadherin (R&D Systems, AF748), Dilution 1:200<br>4.Rabbit polyclonal anti-mouse/human Oct4 (Abcam, Ab19857), Dilution 1:1000<br>5.Mouse monoclonal anti-mouse/rat/human Oct-3/4 (Santa Cruz, Sc-5279), Dilution 1:200, clone name: Oct-3/4 (C-10)<br>6.Mouse monoclonal anti-Flag (Sigma-Aldrich, Merck, F3165), Dilution 1:1000, clone name: M2<br>7.Mouse monoclonal anti-mouse Cdx2 (BioGenex, MU392A-UC), Dilution 1:100, clone name: CDX2-88<br>8.Mouse monoclonal anti-mouse p120 catenin (BD Transduction Laboratories, BD Biosciences, 610134), Dilution 1:250, clone name: 98/pp120<br>9.Mouse monoclonal anti-mouse Histone H3 (Abcam, Ab10799), Dilution 1:2000, clone name: mAbcam 10799<br>10.Rat monoclonal anti-mouse CD31 (PECAM-1) conjugated with APC (BD Pharmingen, BD Biosciences, 551262), Dilution 1:100, clone name: MEC13.3 (RUO)<br>11.Rat monoclonal anti-Mouse c-KIT (CD117) conjugated with APC (BD Pharmingen, BD Biosciences, 561074), Dilution 1:500, clone name: 2B8 (RUO)<br>12.Mouse anti-Mouse SSEA1 conjugated with Alexa Fluor 647 (BD Pharmingen, BD Biosciences, 560120), Dilution 1:50, clone name: MC480 (RUO)<br><br>Secondary antibodies including<br>1.Donkey anti-Goat IgG (H+L) Secondary Antibody-Alexa Fluor 488 (ThermoFisher, A11055), Dilution 1:800<br>2.Donkey anti-Goat IgG (H+L) Secondary Antibody-Alexa Fluor 568 (ThermoFisher, A11057), Dilution 1:800<br>3.Donkey anti-Goat IgG (H+L) Secondary Antibody-Alexa Fluor 647 (ThermoFisher, A21447), Dilution 1:800<br>4.Donkey anti-Mouse IgG (H+L) Secondary Antibody-Alexa Fluor 488 (ThermoFisher, A21202), Dilution 1:800<br>5.Donkey anti-Mouse IgG (H+L) Secondary Antibody-Alexa Fluor 568 (ThermoFisher, A10037), Dilution 1:800
Validation	Primary and secondary antibodies were verified by the companies.<br>Primary antibodies including<br>1.Goat polyclonal anti-mouse Klf4 (R&D Systems, AF3158), https://www.rndsystems.com/products/mouse-klf4-antibody_af3158<br>2.Goat polyclonal anti-human Gata6 (R&D Systems, AF1700), https://www.rndsystems.com/products/human-gata-6-antibody_af1700<br>3.Goat polyclonal anti-human/mouse E-cadherin (R&D Systems, AF748), https://www.rndsystems.com/products/human-mouse-e-cadherin-antibody_af748<br>4.Rabbit polyclonal anti-mouse/human Oct4 (Abcam, Ab19857), https://www.abcam.com/oct4-antibody-ab19857.html<br>5.Mouse monoclonal anti-mouse/rat/human Oct-3/4 (Santa Cruz, Sc-5279), https://www.scbt.com/p/oct-3-4-antibody-c-10<br>6.Mouse monoclonal anti-Flag (Sigma-Aldrich, Merck, F3165), https://www.sigmaaldrich.com/TH/en/product/sigma/f3165<br>7.Mouse monoclonal anti-mouse Cdx2 (BioGenex, MU392A-UC), http://store.biogenex.com/us/applications/ihc/controls/

controls/anti-cdx-2-clone-cdx2-88.html
8.Mouse monoclonal anti-mouse p120 catenin (BD Transduction Laboratories, BD Biosciences, 610134), https://www.bdbiosciences.com/en-nz/products/reagents/microscopy-imaging-reagents/immunofluorescence-reagents/purified-mouse-anti-p120-catenin.610133
9.Mouse monoclonal anti-mouse Histone H3 (Abcam, Ab10799), https://www.abcam.com/histone-h3-antibody-mabcam-10799-chip-grade-ab10799.html
10.Rat monoclonal anti-mouse CD31 (PECAM-1) conjugated with APC (BD Pharmingen, BD Biosciences, 551262), https://www.bdbiosciences.com/en-us/products/reagents/flow-cytometry-reagents/research-reagents/single-color-antibodies-ruo/apc-rat-anti-mouse-cd31.551262
11.Rat monoclonal anti-Mouse c-KIT (CD117) conjugated with APC (BD Pharmingen, BD Biosciences, 561074), https://www.bdbiosciences.com/en-us/products/reagents/flow-cytometry-reagents/research-reagents/single-color-antibodies-ruo/apc-rat-anti-mouse-cd117.561074
12.Mouse anti-Mouse SSEA1 conjugated with Alexa Fluor® 647 (BD Pharmingen, BD Biosciences, 560120), https://www.bdbiosciences.com/en-us/products/reagents/flow-cytometry-reagents/research-reagents/single-color-antibodies-ruo/alexa-fluor-647-mouse-anti-ssea-1.560120

Secondary antibodies including
1.Donkey anti-Goat IgG (H+L) Cross-Absorbed Secondary Antibody-Alexa Fluor 488 (ThermoFisher, A11055), https://www.thermofisher.com/antibody/product/Donkey-anti-Goat-IgG-H-L-Cross-Adsorbed-Secondary-Antibody-Polyclonal/A-11055
2.Donkey anti-Goat IgG (H+L) Secondary Antibody-Alexa Fluor 568 (ThermoFisher, A11057), https://www.thermofisher.com/antibody/product/Donkey-anti-Goat-IgG-H-L-Cross-Adsorbed-Secondary-Antibody-Polyclonal/A-11057
3.Donkey anti-Goat IgG (H+L) Secondary Antibody-Alexa Fluor 647 (ThermoFisher, A21447), https://www.thermofisher.com/antibody/product/Donkey-anti-Goat-IgG-H-L-Cross-Adsorbed-Secondary-Antibody-Polyclonal/A-21447
4.Donkey anti-Mouse IgG (H+L) Secondary Antibody-Alexa Fluor 488 (ThermoFisher, A21202), https://www.thermofisher.com/antibody/product/Donkey-anti-Mouse-IgG-H-L-Highly-Cross-Adsorbed-Secondary-Antibody-Polyclonal/A-21202
5.Donkey anti-Mouse IgG (H+L) Secondary Antibody-Alexa Fluor 568 (ThermoFisher, A10037), https://www.thermofisher.com/antibody/product/Donkey-anti-Mouse-IgG-H-L-Highly-Cross-Adsorbed-Secondary-Antibody-Polyclonal/A10037

The above antibodies are also used routinely in our laboratory and have been tested on numerous protocols over multiple previous studies (Brickman and Morrison, 2006; Livigni et al., 2013) and shown to be specific.

Eukaryotic cell lines

Policy information about cell lines

Cell line source(s)	1.ZHBTc4 ESCs, Oct4 null mouse embryonic stem cells carrying a tetracycline (Tc)-suppressable Oct4 transgene (Niwa et al., 2000) and E14Tg2A or E14Ju (Control murine ES cell lines, Smith and Hooper, 1987 and derived in house at the Institute for Stem Cell Research, University of Edinburgh respectively). ZHBTc4 ESC cell line was gifted by Hitoshi Niwa (Institute of Molecular Embryology and Genetics, Kumamoto University).<br>2.Nanog-GFP MEFs were derived from Nanog-eGFP mouse embryos at embryonic stage 13.5. Nanog-eGFP mice were obtained from Ian Chambers, University of Edinburgh (See "Laboratory animals" below)
Authentication	1.ZHBTc4 ESCs were routinely checked for the maintenance of self-renewal and pluripotency properties using alkaline phosphatase (AP) staining, immunofluorescence, qRT-PCR and flow cytometry.<br>2.iPSC lines were karyotyped before further analysis.
Mycoplasma contamination	ES cells/iPSCs were routinely tested for mycoplasma contamination by PCR. All cell lines were tested negative for mycoplasma.
Commonly misidentified lines<br>(See ICLAC register)	No commonly misidentified cell lines were used in this study.

Animals and other organisms

Policy information about studies involving animals; ARRIVE guidelines recommended for reporting animal research

Laboratory animals	The embryos were from the cross of male Nanog-eGFP mice (Ian Chambers, University of Edinburgh) (age 6-10 months old) with female 129S2/ScPasCrl (Charles Reiver) (8 weeks old). Embryos at embryonic stage 13.5 were collected for MEF derivation. Mice were kept in rooms at a temperature of 22C (±2C), with a humidity of 55% (±10%), air in the room was changed 8–10 times/hour, according to Danish animal experiments regulations.
Wild animals	Catshark females were purchased from local fishermen, transported to the Banyuls sur Mer Oceanological Observatory in oxygenated sea water at 16°C (transport authorisation n°66082) and housed in the Observatory dedicated infrastructures during the spawning season (agreement n°A6601602). They were then released in the wild by their site of collection.
Field-collected samples	No field collected samples were used in the study.
Ethics oversight	1.Mice were maintained, bred, and manipulated at University of Copenhagen, SUND transgenic core facility authorized by the Danish National Animal Experiments Inspectorate (Dyreforsøgstilsynet, license nos. 2012-15-2934-00142 and 2013-15-2934-00935). Animal work in the Brickman lab was also authorized by the Danish National Animal Experiments Inspectorate (Dyreforsøgstilsynet, license no. 2018-15-0201-01520) and performed according to national guidelines.

2.In the catshark, experimental work consisted of fixation of euthanised catshark (non-mammalian) embryos prior to hatching and prior to central nervous system differentiation, which does not require ethic agreement according to national regulations (license number A6601601).

Note that full information on the approval of the study protocol must also be provided in the manuscript.

Flow Cytometry

Plots

Confirm that:

- [x] The axis labels state the marker and fluorochrome used (e.g. CD4-FITC).
- [x] The axis scales are clearly visible. Include numbers along axes only for bottom left plot of group (a 'group' is an analysis of identical markers).
- [x] All plots are contour plots with outliers or pseudocolor plots.
- [x] A numerical value for number of cells or percentage (with statistics) is provided.

Methodology

Sample preparation	TNG ES/iPSC cells were stained with appropriated dilution of fluorescence-conjugated antibodies in FACS buffer (10% FBS in PBS) with 15-minute incubation at 4 C (Dark). The cells were washed three times with FACS buffer and re-suspended in cold FACS buffer containing DAPI (100 ng/mL). DAPI was used in all flow cytometry analysis to exclude dead cells. All experiments included unstained E14Tg2A ESCs as non-fluorescent control required for the gating process.
Instrument	Acquisition of the data was done using a BD LSR Fortessa with 5 spatially-separated laser excitation lines (488nm, 405nm, 355nm, 630nm, 561nm).
Software	BD FACSDiva Software version 6.1.3 from BD BioSciences was used for data acquisition, while FCS Express v.6 from DeNovo Software was used to analyze the acquired data.
Cell population abundance	The average % of the population that was single cells sorted ranged between 1.2%-37.5% based on the samples and nature of iPSC cell lines derived from different Oct4 homologues.
Gating strategy	FSC-A/SSC-A gates were set to select cell population of interest and exclude cell debris. FSC-H/FSC-W gates were set to select single cell population. DAPI-A/FSC-A gates were used to select only live cells and exclude dead cells (high DAPI). This DAPI low/negative gates were then used to determine cell populations with/without c-KIT-APC and Nanog-eGFP. The boundary between positive and negative cell populations was set using unstained E14Tg2A ESCs.

- [x] Tick this box to confirm that a figure exemplifying the gating strategy is provided in the Supplementary Information.
